# Oncogenic role and potential regulatory mechanism of fatty acid binding protein 5 based on a pan-cancer analysis

**DOI:** 10.1038/s41598-023-30695-9

**Published:** 2023-03-11

**Authors:** Jinhua Wang, Siqi Zhao, Jian Sun, Xiaobo Wang, Mingze Guan, Jiajun Yin, Bo Tang

**Affiliations:** 1 Department of Hematology, Second Affiliated Hospital of Dalian Medical University, Dalian, 116044 China; 2 Department of General Surgery, Affiliated Zhongshan Hospital of Dalian University, Dalian, 116300 China

**Keywords:** Cancer, Computational biology and bioinformatics

## Abstract

As one member of fatty acid binding proteins (FABPs), FABP5 makes a contribution in the occurrence and development of several tumor types, but existing analysis about FABP5 and FABP5-related molecular mechanism remains limited. Meanwhile, some tumor patients showed limited response rates to current immunotherapy, and more potential targets need to be explored for the improvement of immunotherapy. In this study, we made a pan-cancer analysis of FABP5 based on the clinical data from The Cancer Genome Atlas database for the first time. FABP5 overexpression was observed in many tumor types, and was statistically associated with poor prognosis of several tumor types. Additionally, we further explored FABP5-related miRNAs and corresponding lncRNAs. Then, miR-577-FABP5 regulatory network in kidney renal clear cell carcinoma as well as CD27-AS1/GUSBP11/SNHG16/TTC28-AS1-miR-22-3p-FABP5 competing endogenous RNA regulatory network in liver hepatocellular carcinoma were constructed. Meanwhile, Western Blot and reverse transcription quantitative real-time polymerase chain reaction (RT-qPCR) analysis were used to verify miR-22-3p-FABP5 relationship in LIHC cell lines. Moreover, the potential relationships of FABP5 with immune infiltration and six immune checkpoints (CD274, CTLA4, HAVCR2, LAG3, PDCD1 and TIGIT) were discovered. Our work not only deepens the understanding of FABP5’s functions in multiple tumors and supplements existing FABP5-related mechanisms, but also provides more possibilities for immunotherapy.

## Introduction

Fatty acid binding proteins (FABPs), serving as noncatalytic binding proteins with complex mechanism and functional diversity, have been considered to play an important role in the modulation of lipid fluxes, signal transduction as well as metabolism^1,2^. As one member of this family, FABP5 presents unique characteristics in various specific cells and tissues including adipocytes, macrophages and tumor cells^2^. Jing et al*.* first identified FABP5 as a tumor-promoting gene^3,4^, and other researchers have also confirmed its role in several tumor types^5–8^. Studies have reported that FABP5 promotes cell proliferation, progression and invasion of tumors via fatty acid uptake oxidation and several signaling such as PPAR β/δ and HIF-1α^2,9,10^. Additionally, the prognostic value of FABP5 in several tumor types has also been revealed by several studies. For example, FABP5 is associated with poor prognosis in clear cell renal cell carcinoma, gastric cancer and triple-negative breast cancer^11–13^.

In addition to FABP5, tumor microenvironment (TME), which is composed of stromal cells, immune cells and tumor cells, also makes great contributions in the tumorigenesis and progression^14,15^. Tumor cells and other nonmalignant cells interact with each other, building a microenvironment which is conducive to tumor growth^16^. As a part of stromal cells, cancer associated fibroblasts (CAFs) promote tumor growth and make tumor cells more carcinogenic^17^. Among those immune cells in TME, effector T cells, dendritic cells (DCs), M1 macrophages and natural killer cells serve as antineoplastic factors, while immunosuppressive cells, including regulatory T cells (Tregs) and M2 macrophages, are tumor-promoting factors^15,18^. Tumor immune escape is one of vital mechanisms in tumor occurrence and progression. One of main factors in this mechanism is that the above-mentioned immunosuppressive cells influence the immune tolerance of tumor cells^19^. Another factor is that the regulation of immune checkpoints’ expression inhibits effector T cells’ activation^19^. Targeting immune checkpoints has been a hot direction in the current clinical research and considered as one of effective antitumor therapies. Up to date, three immune checkpoints, namely programmed cell death 1 (PD-1/PDCD1), programmed cell death ligand 1 (PD-L1/CD274) and cytotoxic T lymphocyte-associated antigen 4 (CTLA4) have been frequently targeted. Nevertheless, patients under the therapy targeting above immune checkpoints have limited response rates. Therefore, the exploration of potential targets is needed to obtain higher response rates and improve the antitumor therapeutic effectiveness. There have been several novel immune checkpoints, including T cell immunoreceptor with Ig and ITIM domains (TIGIT), T cell immunoglobulin domain and mucin domain 3 (TIM-3/HAVCR2) and lymphocyte activation gene 3 (LAG3) under clinical trials. LAG3 and HAVCR2 have been reported to associate with T cell exhaustion, and TIGIT plays a vital role in the limitation of T cell inflammation^20–22^.

A report has defined a new regulatory network, namely competing endogenous RNA (ceRNA), constituted by the competition between mRNAs, long non-coding RNAs (lncRNAs), pseudogene transcripts and circRNAs for the binding of microRNA (miRNA)^23^. This novel regulatory network has been confirmed to have a great role in tumor occurrence and development^24–26^. Among different kinds of ceRNA networks currently discovered, a ceRNA network targeting FABP5, namely circ-ABCB10/miR-620/FABP5, have been proved to participate in the occurrence and development of glioma^27^. Although the potential oncogenic role of FABP5 has been confirmed in multiple tumor types, there is a lack of more comprehensive analyses about FABP5 and corresponding molecular mechanism. For further understanding of FABP5-related molecular mechanism in multiple tumor types, more ceRNA regulatory networks need to be explored.

Taken together, our study made a pan-cancer analysis of FABP5 on the basis of the clinical data from The Cancer Genome Atlas (TCGA) database for the first time, and observed novel FABP5-related regulatory networks in kidney renal clear cell carcinoma (KIRC) and liver hepatocellular carcinoma (LIHC). Meanwhile, miR-22-3p-FABP5 relationship was verified in LIHC cell lines for the first time. Besides, we evaluated the association between FABP5 and immune infiltration, and further analyzed the association between FABP5 and six immune checkpoints. Our work mainly aimed to supplement and improve existing FABP5-related molecular and immunological mechanisms, and provide more possibilities for immunotherapy.

## Results

### Expression analysis of FABP5

**Figure 1:**
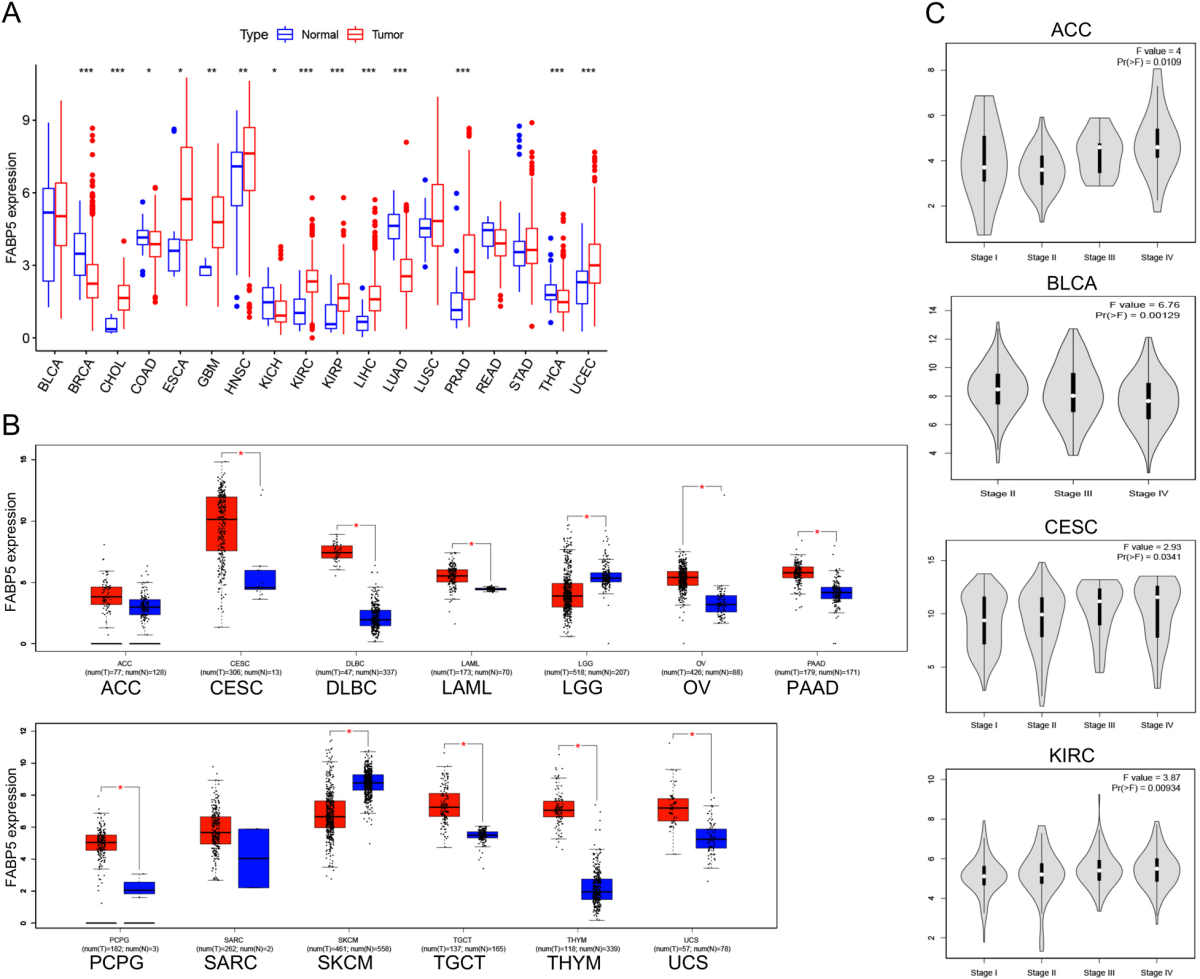
(**A**) Expression analysis of FABP5 based on TCGA database. *p < 0.05; **p < 0.01; ***p < 0.001. (**B**) Expression analysis of FABP5 based on TCGA and GTEx database. *p < 0.05. (**C**) Correlation between FABP5 expression and pathological stages of tumors.

We conducted expression analysis of FABP5 in multiple tumor types with sufficient normal tissues (more than five cases) in TCGA project. Compared with normal tissues, we observed the up-regulated expression of FABP5 in the tumor tissues of cholangiocarcinoma (CHOL), esophageal carcinoma (ESCA), glioblastoma multiforme (GBM), head and neck squamous cell carcinoma (HNSC), KIRC, kidney renal papillary cell carcinoma (KIRP), LIHC, prostate adenocarcinoma (PRAD) and uterine corpus endometrial carcinoma (UCEC) (ESCA *p* < 0.05, GBM and HNSC *p* < 0.01, and other tumor types *p* < 0.001), but down-regulated expression in tissues of breast invasive carcinoma (BRCA), colon adenocarcinoma (COAD), kidney chromophobe (KICH), lung adenocarcinoma (LUAD) and thyroid carcinoma (THCA) (COAD and KICH *p* < 0.05, and other tumor types *p* < 0.001) (Fig. 1A).

Then we added normal tissues from GTEx database as controls, and further analyzed the difference of FABP5 expression between the normal and tumor tissues in other thirteen tumor types (Fig. 1B). Compared with normal tissues, we found that FABP5 expression was up-regulated in the tumor tissues of cervical squamous cell carcinoma and endocervical adenocarcinoma (CESC), lymphoid neoplasm diffuse large B-cell lymphoma (DLBC), acute myeloid leukemia (LAML), ovarian serous cystadenocarcinoma (OV), pancreatic adenocarcinoma (PAAD), pheochromocytoma and paraganglioma (PCPG), testicular germ cell tumors (TGCT), Thymoma (THYM) and uterine carcinosarcoma (UCS), but down-regulated in brain lower grade glioma (LGG) and skin cutaneous melanoma (SKCM) (all *p* < 0.05).

Furthermore, we investigated the potential association between FABP5 expression and pathological stages of tumors. FABP5 expression was associated with pathological stages of Adrenocortical carcinoma (ACC), BLCA, CESC and KIRC (ACC *p* = 0.011, BLCA *p* = 0.001, CESC *p* = 0.034 and KIRC *p* = 0.009, Fig. 1C). As the tumor progresses, FABP5 expression increased in the cases of CESC and KIRC, but decreased in BLCA.

### Survival analysis of FABP5

**Figure 2:**
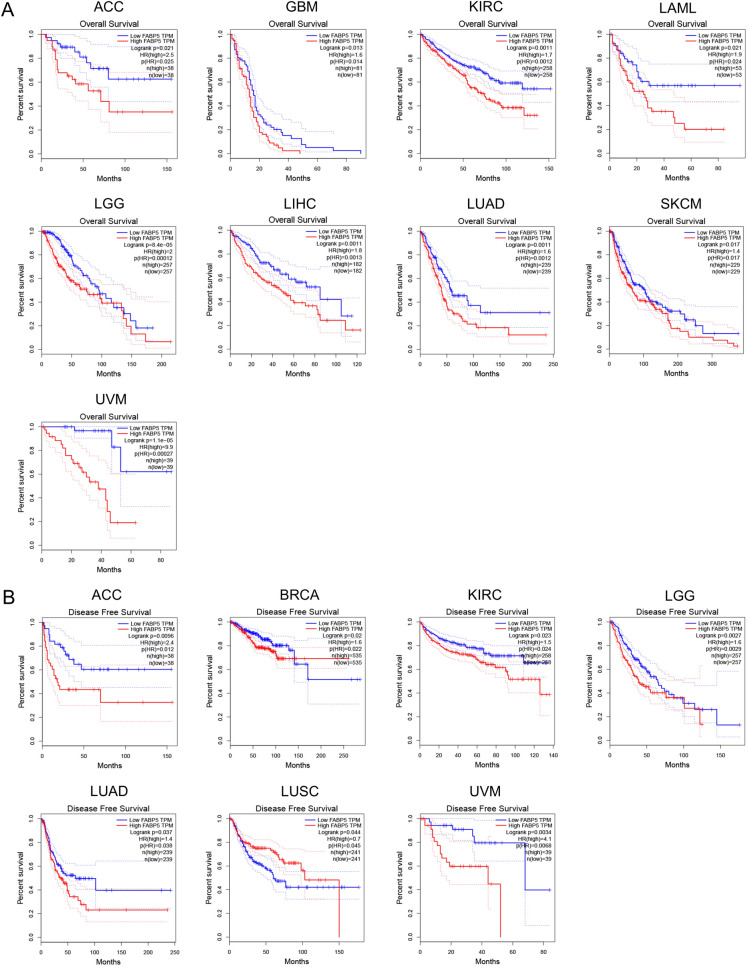
Survival analysis of FABP5. (**A**) Overall survival analysis of FABP5. (**B**) Disease-free survival analysis of FABP5.

Survival analysis was conducted by GEPIA to investigate the prognostic value of FABP5. High expression of FABP5 was associated with poor overall survival (OS) in ACC, GBM, KIRC, LAML, LGG, LIHC, LUAD, SKCM and uveal melanoma (UVM) (ACC *p* = 0.025, GBM *p* = 0.014, KIRC *p* = 0.001, LAML *p* = 0.024, LIHC *p* = 0.001, LUAD *p* = 0.001, SKCM *p* = 0.017, and other tumor types *p* < 0.001, Fig. 2A). Additionally, the results of disease-free survival (DFS) analysis showed that high FABP5 expression was statistically associated with poor prognosis in ACC, BRCA, KIRC, LGG, LUAD and UVM, but favorable prognosis in lung squamous cell carcinoma (LUSC) (ACC *p* = 0.012, BRCA *p* = 0.022, KIRC *p* = 0.024, LGG *p* = 0.003, LUAD *p* = 0.038, LUSC *p* = 0.045, UVM *p* = 0.007, Fig. 2B).

Survival analysis was also conducted by the Kaplan–Meier survival curve and Log-rank test in those tumor types with FABP5 overexpression. We found that high FABP5 expression was associated with poor OS prognosis by the Kaplan–Meier survival curve and Log-rank test simultaneously in only three tumor types, namely GBM, KIRC and LIHC (Table 1).

**Table 1:** Survival analysis by the Kaplan–Meier survival curve and Log-rank test in GBM, KIRC and LIHC.

Tumor types	KM	HR	HR.95L	HR.95H	coxPvalue
GBM	0.047745	1.158051	1.029553	1.302586	0.014473
KIRC	0.000124	1.557255	1.298163	1.868056	1.84E-06
LIHC	0.001774	1.369035	1.151159	1.628148	0.000383

### Prediction and analysis of upstream miRNAs

**Figure 3:**
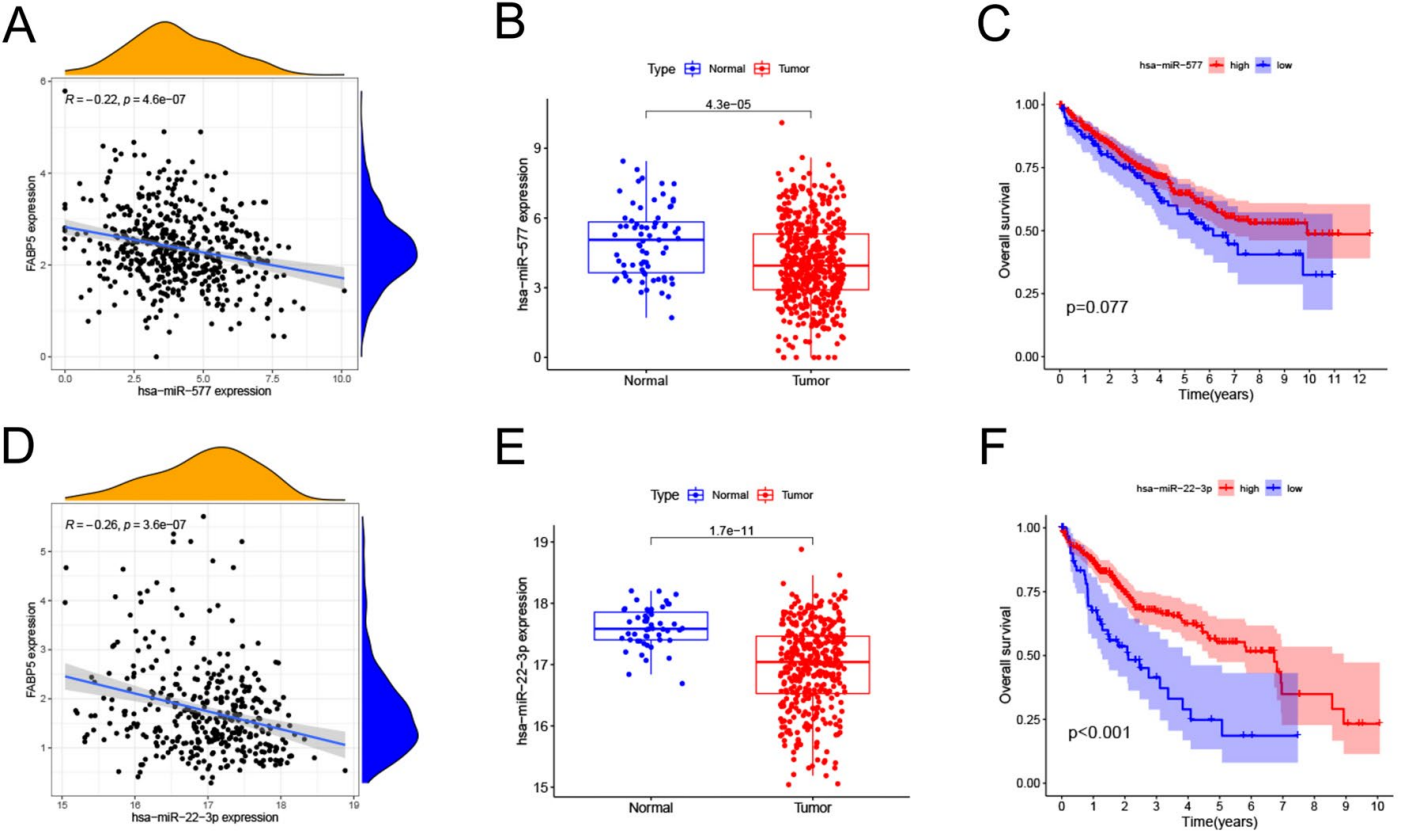
(**A**) Correlation analysis between FABP5 and miR-577 in KIRC. (**B**) Expression analysis of miR-577 in KIRC. (**C**) Overall survival analysis of miR-577 in KIRC. (**D**) Correlation analysis of FABP5 with miR-22-3p in LIHC. (**E**) Expression analysis of miR-22-3p in LIHC. (**F**) Overall survival analysis of miR-22-3p in LIHC.

Considering that there is no corresponding miRNA transcriptome data of GBM available in the TCGA database, we narrowed our study to two tumor types, namely KIRC and LIHC. Using ENCORI approach, we predicted potential upstream miRNAs, all of which have already been confirmed by CLIP-Seq experiment to have interactions with FABP5. Seventeen predicted miRNAs were shown in Table 2. In KIRC, there were only one miRNA, namely miR-577, significantly negatively correlated with FABP5 (R = − 0.22, *p* < 0.001, Fig. 3A). Down-regulated miR-577 expression was observed in KIRC tissues compared with normal tissues (*p* < 0.001, Fig. 3B). Besides, KIRC patients with low miR-577 expression had poor OS (*p* = 0.077, Fig. 3C). In LIHC, we also found only one miRNA, namely miR-22-3p, having significantly negative correlation with FABP5 (R = − 0.26, *p* < 0.001, Fig. 3D). MicroRNA-22-3p expression was down-regulated in tumor tissues compared with normal tissues (*p* < 0.001, Fig. 3E), and low miR-22-3p expression was associated with poor OS in LIHC (*p* < 0.001, Fig. 3F).

**Table 2:** Predicted upstream miRNAs of FABP5.

Gene	Upstream miRNAs
FABP5	hsa-miR-17-5p; hsa-miR-20a-5p; hsa-miR-22-3p; hsa-miR-93-5p; hsa-miR-106a-5p; hsa-miR-142-5p; hsa-miR-106b-5p; hsa-miR-302a-3p; hsa-miR-302b-3p; hsa-miR-302c-3p; hsa-miR-302d-3p; hsa-miR-380-3p; hsa-miR-20b-5p; hsa-miR-329-3p; hsa-miR-409-3p; hsa-miR-577; hsa-miR-362-3p

### Primary validation of the predicted association between miR-22-3p and FABP5 in LIHC

**Figure 4:**
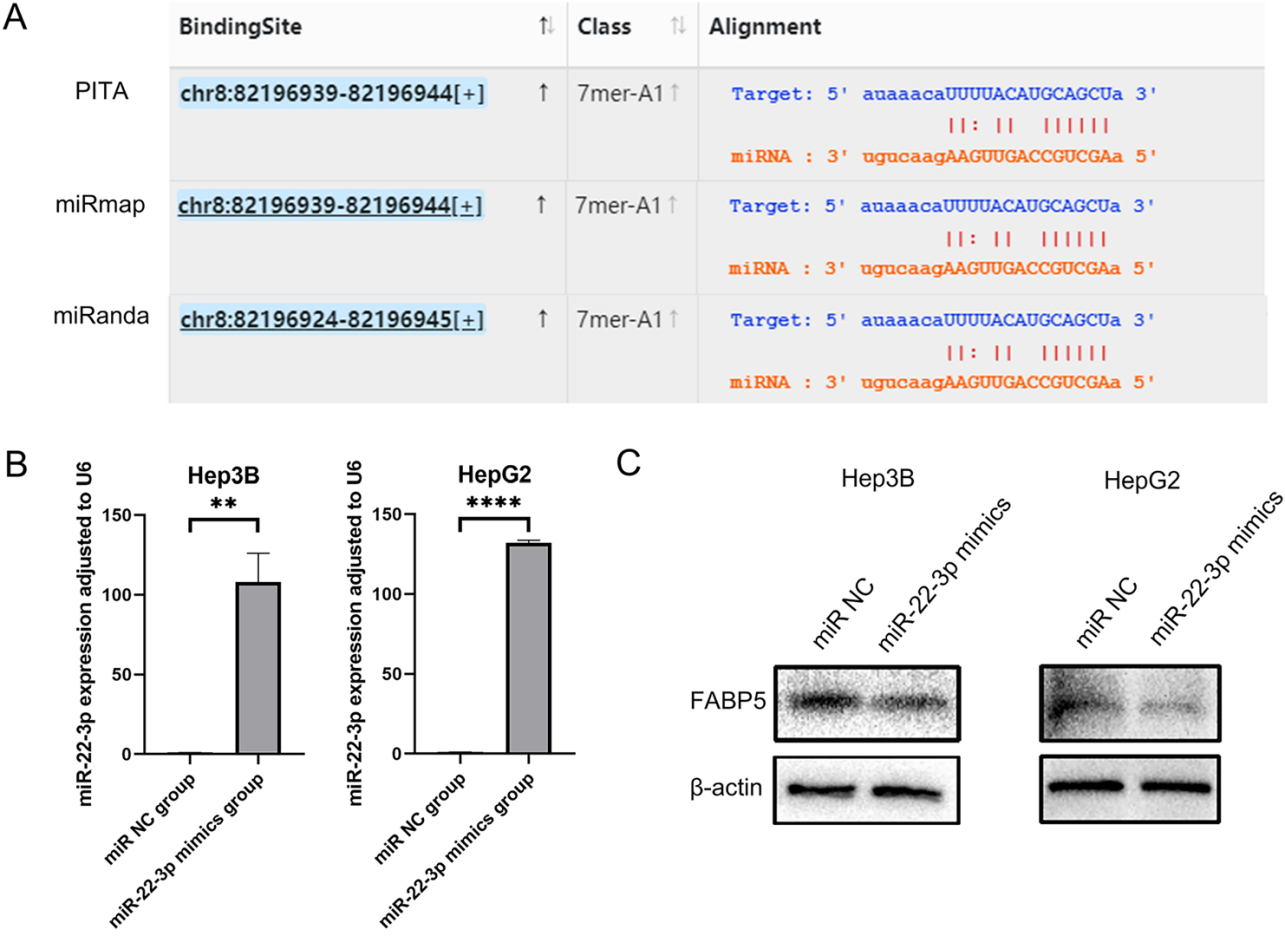
(**A**) Binding sites of miR-22-3p on FABP5 predicted by PITA, miRmap and miRanda. (**B**) MicroRNA-22-3p expression measured in Hep3B and HepG2 cell lines via RT-qPCR. (**C**) The protein expression of FABP5 detected in Hep3B and HepG2 cell lines via Western Blot. **p < 0.01; ****p < 0.0001.

Binding sites of miR-22-3p on FABP5 predicted by PITA, miRmap and miRanda were shown in Fig. 4A. To test the accuracy of the miRNA-mRNA interactions predicted via ENCORI approach and ensure the innovation of the study, we chose LIHC cell lines for the validation of the potential miR-22-3p-FABP5 association that has not been reported in any tumor type yet. Our study transfected miR-22-3p mimics into two different LIHC cell lines (Hep3B and HepG2). The results of reverse transcription quantitative real-time polymerase chain reaction (RT-qPCR) presented higher miR-22-3p expression in miR-22-3p mimics group compared with miR negative control (NC) group in both Hep3B and HepG2 cell lines (Fig. 4B), reflecting the transfection effectiveness of miR-22-3p. Then we detected the FABP5 protein expression in each group, and found lower FABP5 protein expression in miR-22-3p mimics group compared with miR NC group (Fig. 4C). Combining the results of RT-qPCR and Western Blot, we discovered that miR-22-3p reduced FABP5 protein expression in both two LIHC cell lines.

### Prediction and analysis of upstream lncRNAs

**Figure 5:**
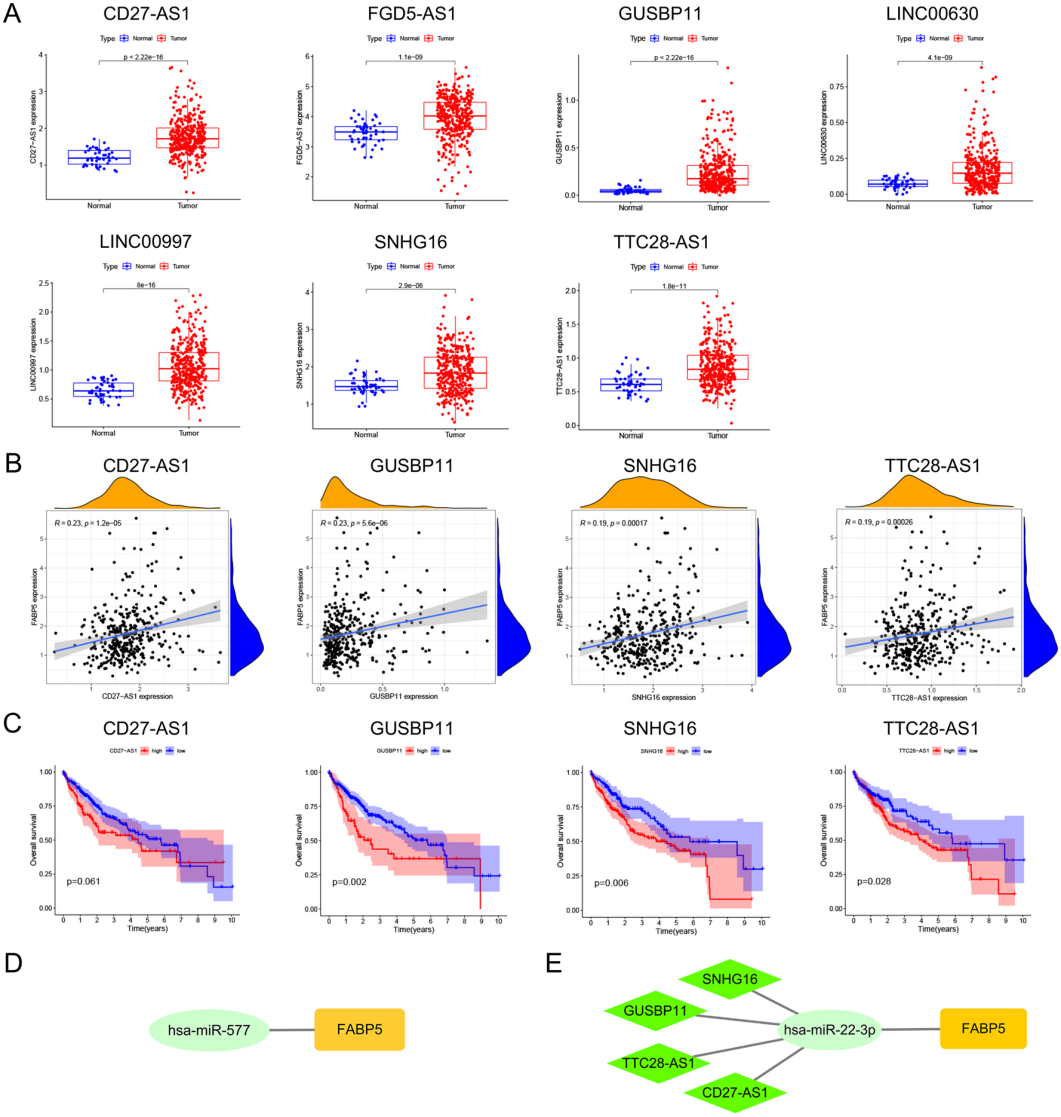
(**A**) Expression analysis of CD27-AS1, FGD5-AS1, GUSBP11, LINC00630, LINC00997, SNHG16 and TTC28-AS1 in LIHC. (**B**) Correlation analysis of FABP5 with CD27-AS1, GUSBP11, SNHG16 and TTC28-AS1 in LIHC. (**C**) Overall survival analysis of CD27-AS1, GUSBP11, SNHG16 and TTC28-AS1 in LIHC. (**D**) FABP5-related regulatory network in KIRC. (**E**) FABP5-related ceRNA regulatory network in LIHC.

To further investigate the molecular biological mechanisms of FABP5 in KIRC and LIHC, we explored the upstream lncRNAs of miR-577 and miR-22-3p, and then made corresponding analysis. In KIRC, although several lncRNAs had negative associations with miR-577, all of those associations were not significant (− 0.2 < cor < 0, Table 3). In LIHC, SNHG16, GUSBP11, FGD5-AS1, TTC28-AS1, LINC00630, CD27-AS1, LINC02381, H19 and LINC00997 presented notably negative correlations with miR-22-3p (Table 4). Among those lncRNAs, only seven lncRNAs, namely CD27-AS1, FGD5-AS1, GUSBP11, LINC00630, LINC00997, SNHG16 and TTC28-AS1, showed higher expression in LIHC tissues compared with normal tissues (all *p* < 0.001, Fig. 5A). Then we conducted correlation analysis of FABP5 with those seven lncRNAs. The results showed that only four lncRNAs, including CD27-AS1 (R = 0.23), GUSBP11 (R = 0.23), SNHG16 (R = 0.19) and TTC28-AS1 (R = 0.19), were positively associated with FABP5 (all *p* < 0.001, Fig. 5B). Survival analysis of above four lncRNAs (Fig. 5C) showed that high expression of GUSBP11 (*p* = 0.002), SNHG16 (*p* = 0.006) and TTC28-AS1 (*p* = 0.028) was statistically related to poor prognosis of LIHC patients.

**Table 3:** Upstream lncRNAs negatively associated with miRNA-577 in KIRC.

lncRNA	miRNA	cor	p value	logFC	diffPval
DUXAP8	hsa-miR-577	− 0.15923	0.000278	0.207812	1.86E − 27
SNHG3	hsa-miR-577	− 0.14239	0.001169	0.483696	1.71E − 08
GASAL1	hsa-miR-577	− 0.11553	0.008554	0.085325	0.000269
NIFK-AS1	hsa-miR-577	− 0.11356	0.00976	− 0.21328	2.00E − 05
LINC00652	hsa-miR-577	− 0.09631	0.028555	− 0.21525	6.52E − 35

**Table 4:** Upstream lncRNAs significantly negatively associated with miR-22-3p in LIHC.

lncRNA	miRNA	cor	p value	logFC	diffPval
SNHG16	hsa-miR-22-3p	− 0.3967	2.14E − 15	0.373458	2.89E − 06
GUSBP11	hsa-miR-22-3p	− 0.36983	1.95E − 13	0.195542	1.50E − 22
FGD5-AS1	hsa-miR-22-3p	− 0.31769	4.03E − 10	0.510452	1.13E − 09
TTC28-AS1	hsa-miR-22-3p	− 0.28953	1.41E − 08	0.26424	1.82E − 11
LINC00630	hsa-miR-22-3p	− 0.2842	2.65E − 08	0.101263	4.06E − 09
CD27-AS1	hsa-miR-22-3p	− 0.24503	1.84E − 06	0.553462	1.89E − 17
LINC02381	hsa-miR-22-3p	− 0.23506	4.87E − 06	0.375609	0.993627
H19	hsa-miR-22-3p	− 0.2198	1.99E − 05	− 1.44865	2.16E − 07
LINC00997	hsa-miR-22-3p	− 0.21738	2.47E − 05	0.414374	8.01E − 16

Combining those findings, we obtained two novel regulatory networks of FABP5, namely miR-577-FABP5 regulatory network in KIRC (Fig. 5D) and CD27-AS1/GUSBP11/SNHG16/TTC28-AS1-miR-22-3p-FABP5 ceRNA regulatory network in LIHC (Fig. 5E).

### Correlation analysis of FABP5 with immune infiltration

**Figure 6:**
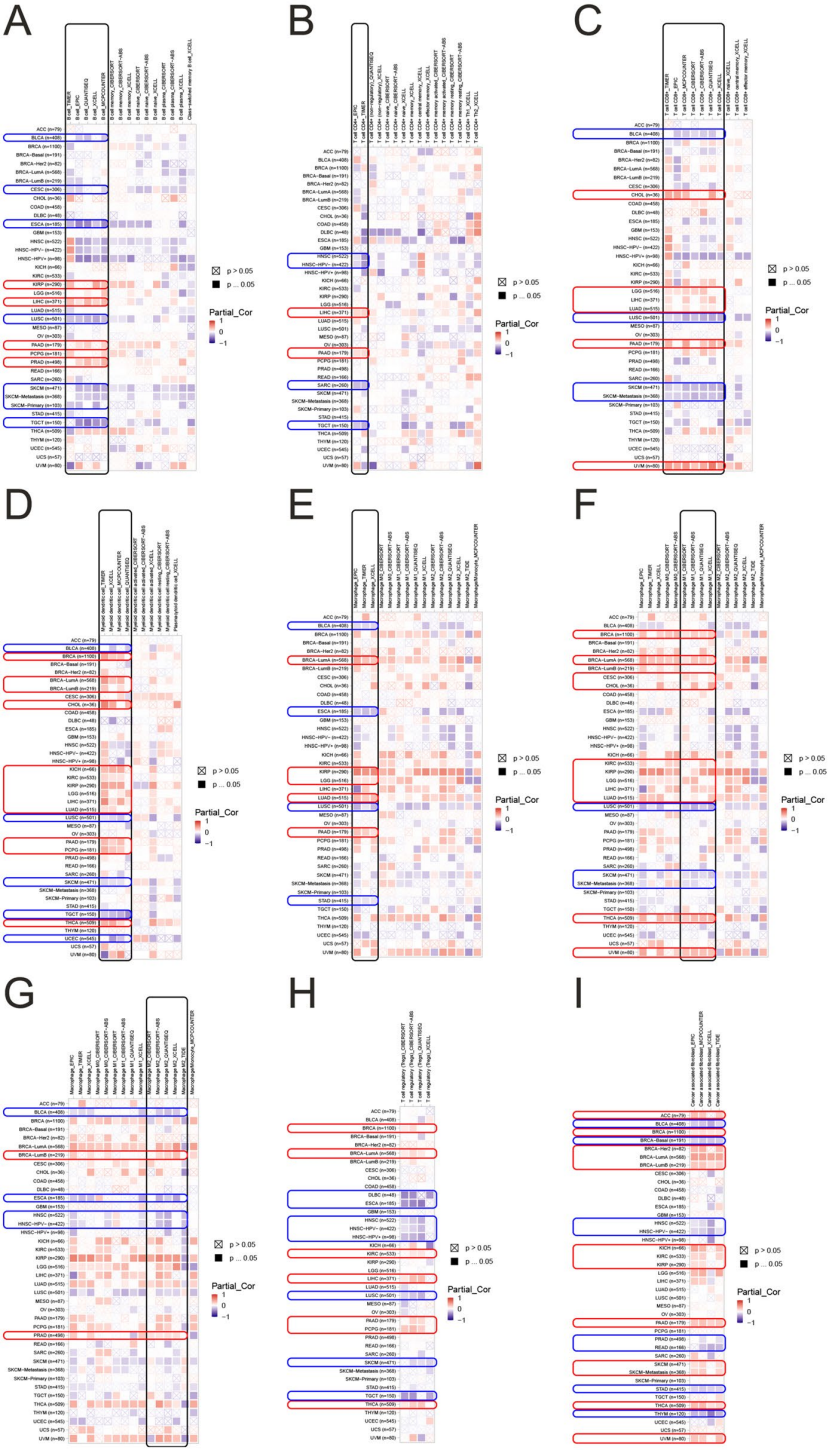
(**A**) Correlation between FABP5 and B cell infiltration. (**B**) Correlation between FABP5 and CD4+ T cell infiltration. (**C**) Correlation between FABP5 and CD8+ T cell infiltration. (**D**) Correlation between FABP5 and DC infiltration. (**E**) Correlation between FABP5 and macrophage infiltration. (**F**) Correlation between FABP5 and M1 macrophage infiltration. (**G**) Correlation between FABP5 and M2 macrophage infiltration. (**H**) Correlation between FABP5 and Treg infiltration. (**I**) Correlation between FABP5 and CAF infiltration.

Using TIMER2 approach, we explored the potential relationship between FABP5 expression and immune infiltrations in different tumor types. Most of algorithms showed a positive association between B cell infiltration and FABP5 expression in KIRP, LIHC, PAAD and PRAD, but a negative relationship in BLCA, CESC, ESCA, LUSC, SKCM, SKCM-Metastasis, SKCM-Primary and TGCT (Fig. 6A). As for CD4+ T-cells, there was a positive correlation between the infiltration and FABP5 expression in LIHC and PAAD, but a negative correlation in HNSC, HNSC-HPV−, Sarcoma (SARC) and TGCT based on EPIC and TIMER (Fig. 6B). CD8+ T cell infiltration showed a positive association with FABP5 expression in CHOL, LGG, LIHC, LUAD, PAAD and UVM, but negative association in BLCA, LUSC, SKCM and SKCM-Metastasis based on most of algorithms (Fig. 6C). As shown in Fig. 6D, DCs’ infiltration was positively associated with FABP5 in BRCA, BRCA-LumA, BRCA-LumB, CHOL, KICH, KIRC, KIRP, LGG, LIHC, LUAD, PAAD, PCPG and THCA, but negatively in BLCA, LUSC, SKCM, TGCT and UCEC. Based on EPIC, TIMER and XCELL algorithms, infiltration of macrophages presented a positive relationship with FABP5 expression in BRCA-LumA, KIRP, LGG, LUAD and PAAD, but a negative relationship in BLCA, ESCA, LUSC and STAD (Fig. 6E). Among different types of macrophages, infiltration of M1 macrophages was positively associated with FABP5 expression in BRCA, BRCA-LumA, CESC, CHOL, KIRC, KIRP, LGG, LIHC, LUAD, THCA and UVM, but negatively in LUSC, SKCM and SKCM-Metastasis based on most of algorithms (Fig. 6F). As shown in Fig. 6G, M2 macrophage infiltration was positively related to FABP5 expression in BRCA-LumB and PRAD, but negatively in BLCA, ESCA, HNSC and HNSC-HPV− based on most of algorithms. Most of algorithms showed that infiltration of Tregs had a positive relationship with FABP5 expression in BRCA, BRCA-LumA, KIRC, LIHC, PAAD, PCPG and THCA, but a negative relationship in DLBC, ESCA, HNSC, HNSC-HPV−, HNSC-HPV+, LUSC, SKCM and TGCT (Fig. 6H). Moreover, based on most of algorithms, CAFs’ infiltration presented a positive relationship with FABP5 expression in ACC, BRCA, BRCA-Her2, BRCA-LumA, BRCA-LumB, KICH, KIRC, KIRP, PAAD, SKCM, SKCM-Metastasis, THCA and UVM, but a negative relationship in BLCA, BRCA-Basal, HNSC, HNSC-HPV−, PRAD, READ, STAD and THYM (Fig. 6I).

### Correlation analysis of FABP5 with immune checkpoints

**Figure 7:**
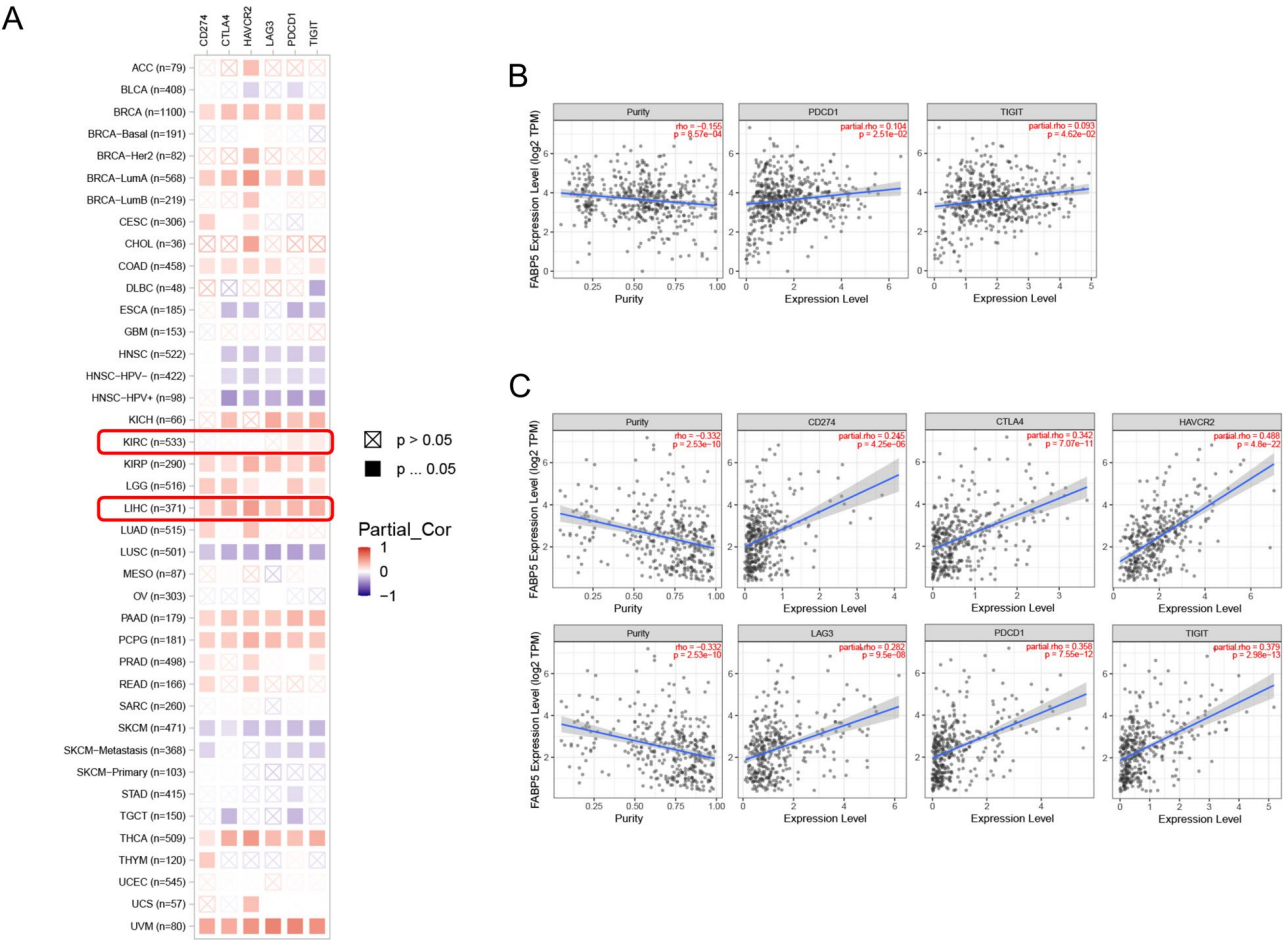
(**A**) Correlation between FABP5 and immune checkpoints in multiple tumor types. (**B**) Correlation between FABP5 and immune checkpoints in KIRC. (**C**) Correlation between FABP5 and immune checkpoints in LIHC.

We investigated the potential relationship between FABP5 expression and several immune checkpoints, including CD274, CTLA4, HAVCR2, LAG3, PDCD1 and TIGIT (Fig. 7A). In KIRC, FABP5 expression presented positive associations with the expression of PDCD1 (Rho = 0.104) and TIGIT (Rho = 0.093) (all *p* < 0.05, Fig. 7B). In LIHC, FABP5 expression was positively associated with the expression of CD274 (Rho = 0.245), CTLA4 (Rho = 0.342), HAVCR2 (Rho = 0.488), LAG3 (Rho = 0.282), PDCD1 (Rho = 0.358) and TIGIT (Rho = 0.379) (all *p* < 0.05, Fig. 7C). In addition to LIHC, FABP5 expression was positively correlated with expression of the above six immune checkpoints in BRCA, BRCA-LumA, KIRP, PAAD, PCPG, THCA and UVM.

## Discussion

FABP5 has been reported to participate in the occurrence and development of different tumors, including KIRC, LIHC, LUAD and cervical cancer^5,10,11,28^. However, there is no study analyzing the potential roles of FABP5 based on a pan-cancer perspective. Therefore, we conducted a pan-cancer analysis of FABP5 based on the data from TCGA and GTEx database. We verified FABP5 overexpression in many tumor types, and partial results obtained support by previous studies^10,29–32^. Moreover, survival analysis showed an association between high FABP5 expression and poor prognosis in several tumor types, suggesting potential prognostic values of FABP5 in those tumors. Some of those results were consistent with the conclusions made by previous studies. High expression level of FABP5 was confirmed to predict poor prognosis in KIRC and LUAD^28,31^. Besides, FABP5 expression was considered as a prognostic marker of LIHC and UVM^33,34^.

Existing knowledge about FABP5-related regulatory networks is still limited, so further exploration of corresponding regulatory networks is needed. Additionally, further exploration may contribute to the discovery of new specific diagnostic and prognostic markers. To date, many studies have reported that ceRNA networks play a great role in multiple tumor types, providing a direction for exploring potential FABP5-related molecular mechanism. In this study, we combined the results of expression and survival analysis, and observed a situation. Only in GBM, KIRC and LIHC, FABP5 was overexpressed, and the overexpression was associated with poor OS prognosis shown by both Kaplan–Meier survival curve and Log-rank test. However, there is no corresponding miRNA transcriptome data of GBM available in the TCGA database. Thus, we did not include this tumor type in the scope of this study for the time being, and the subsequent analyses for FABP5-related upstream regulatory networks mainly focused on the other two tumor types. MicroRNAs mainly function as negative regulatory factors of corresponding genes. In this study, FABP5 functioned as an oncogenic factor in KIRC and LIHC, so upstream miRNAs of FABP5 should be antineoplastic and have significantly negative association with FABP5.

In KIRC, several reports have demonstrated the carcinogenic role of FABP5^7,11,31^. However, we failed to find any articles about FABP5-related regulatory networks, so further exploration is really meaningful and necessary. Among those predicted miRNAs, only miR-577 was significantly negatively associated with FABP5 in KIRC. Although studies have confirmed miR-577’s antineoplastic role in many tumor types^35–38^, the certain role in KIRC has not been reported up to date. Based on our bioinformatics analysis, the expression of miR-577 was down-regulated, and KIRC patients with higher miR-577 expression had favorite outcomes. Meanwhile, we observed that as KIRC progresses, FABP5 expression increased. Therefore, down-regulated miR-577 may influence KIRC occurrence and progression via the regulation of FABP5 expression. Then we constructed miR-577-FABP5 axis and considered it as a regulator in KIRC occurrence and progression, but more experimental evidence is still needed to further improve the reliability of miR-577’s function and this axis’ regulatory effects in KIRC.

In LIHC, we found only one miRNA, namely miR-22-3p, significantly negatively associated with FABP5. Our bioinformatics analysis showed that miR-22-3p expression was down-regulated and lower expression was associated with poor prognosis in LIHC. Above results reflected the potential anti-tumor roles of miR-22-3p, which also obtained supports by the conclusion based on functional experiments in previous study that miR-22-3p suppressed tumor cell proliferation and arrested cell cycle in LIHC^39,40^. Combining those results, we constructed miR-22-3p-FABP5 axis, and considered it as one of potential regulatory networks in LIHC. Meanwhile, further experiment research was conducted to validate the potential miR-22-3p-FABP5 association in LIHC. MicroRNA-22-3p was verified to negatively regulate the expression of FABP5 via Western Blot. At present, there has been no report about the potential miR-22-3p-FABP5 relationship in any tumor type available. Our study verified the negative association between miR-22-3p and FABP5 in one tumor type for the first time, presenting a great novelty and providing a new direction for molecular mechanism exploration in other tumor types with high FABP5 expression. Moreover, it has been confirmed that down-regulated miR-22-3p expression promotes cell mobility and invasiveness of LIHC, and miR-22-3p overexpression has opposite effects^41^, suggesting the potential prognostic and therapeutic value of miR-22-3p. Therefore, we considered miR-22-3p-FABP5 axis as an important factor affecting the prognosis in LIHC, and further exploration of miR-22-3p-FABP5 axis benefits to the therapeutic and prognostic guidance of LIHC.

According to the definition of the ceRNA network, upstream lncRNAs are required to have negative associations with miR-22-3p, but positive associations with FABP5 in LIHC. Among those predicted lncRNAs of miR-22-3p, CD27-AS1, GUSBP11, SNHG16 and TTC28-AS1 met the above requirements, and all of those four lncRNAs were overexpressed in LIHC. Additionally, higher expression of GUSBP11, SNHG16 and TTC28-AS1 was statistically related to poor prognosis, and CD27-AS1 nearly reached statistical significance. A previous study has verified that CD27-AS1, GUSBP11 and SNHG16 serve as regulatory factors in LIHC through miR-22-3p-CCNA2 axis^42^. Our study came to a consistent conclusion that CD27-AS1, GUSBP11 and SNHG16 took miR-22-3p as a target to participate in the occurrence and progression of LIHC. Different from the previous study, we confirmed that FABP5 was another potential target of miR-22-3p in addition to CCNA2, reflecting that CD27-AS1/GUSBP11/SNHG16-miR-22-3p can affect the occurrence and development of LIHC via different pathways. Therefore, we further constructed CD27-AS1/GUSBP11/SNHG16-miR-22-3p-FABP5 ceRNA network, and considered it as a novel regulatory network in LIHC occurrence and progression. Furthermore, many studies have confirmed that SNHG16 promotes tumorigenesis, invasion and metastasis of LIHC by functioning as a sponge for multiple miRNAs^43–46^. The expression of SNHG16, an independent prognostic predictor, is associated with tumor size and sorafenib resistance of LIHC patients^47–49^. Therefore, SNHG16/miR-22-3p/FABP5 axis provides not only more possible prognostic indicators of LIHC, but also potential therapeutic targets for the improvement of chemotherapy’s effectiveness. TTC28-AS1 has been verified to have an association with epithelial–mesenchymal transition in bladder cancer^50^, but its role in LIHC progression remains uncertain. Thus, the view that TTC28-AS1 has carcinogenic effects in LIHC needs more experimental evidence to confirm.

In addition to FABP5 and FABP5-related regulatory networks, TME plays an important role in the occurrence and progression of tumors, and is involved in drug resistance^14,15,51^. Therefore, exploration of the potential relationship between FABP5 expression and immune infiltration provides possibilities for supplementing corresponding immunological mechanism and improving the therapeutic effects. Contrary to those antineoplastic immune cells including CD8+ T-cells, DCs and M1 macrophages, M2 macrophages and Tregs are considered to be tumor-promoting^15,18,51,52^. As for LIHC, we observed a positive relationship between FABP5 expression and the infiltration of Tregs. Due to FABP5 overexpression in LIHC tissues, high FABP5 expression may function as a carcinogenic factor by influencing Treg infiltration in LIHC. Tumor-promoting factors in TME also include CAFs in addition to M2 macrophages and Tregs^17,52^. In KIRC, FABP5 expression presented positive associations with the infiltration of Tregs and CAFs. Combining the results of expression analysis of FABP5, we considered a possibility that high FABP5 expression contributes to KIRC occurrence and progression by regulating the infiltration of Tregs and CAFs.

Moreover, our study conducted the evaluation of the potential relationship between FABP5 expression and six immune checkpoints. Among those checkpoints, PDCD1, CTLA4, LAG3 and HAVCR2 have been reported to show strong associations with each other, and affect the prognosis of tumors^53^. The positive relationship between FABP5 expression and all of those four immune checkpoints in BRCA, BRCA-LumA, KIRP, LIHC, PAAD, PCPG, THCA and UVM reflected that FABP5 may interact with those four immune checkpoints to participate in tumor occurrence and development. As for immunotherapy, antibodies targeting PD-1, PD-L1 and CTLA4 have been confirmed to make a significant improvement in the survival outcomes of patients with tumors, and the combination therapy targeting CTLA4 and PD-1 further improves the response rates and median survival of patients with many tumor types^54,55^. Besides, combination therapy inhibiting TIGIT and PD-1 made a significant improvement of OS in preclinical trials^56^. In this study, we observed that FABP5 expression was positively related to one of above-mentioned immune checkpoints in multiple tumor types, suggesting a possible synergy between FABP5 and one of above immune checkpoints. Further exploration of potential interactions between FABP5 and those immune checkpoints and corresponding experiments are needed to verify whether FABP5 can be a target for immunotherapy.

Taken together, we made a pan-cancer analysis of FABP5 based on TCGA and GTEx database for the first time, and confirmed FABP5 overexpression in most tumor types. We also explored the potential prognostic values of FABP5, and observed that high FABP5 expression was associated with poor outcomes of patients with most of tumor types. Additionally, we further investigated FABP5-related regulatory networks and constructed miR-577-FABP5 regulatory network in KIRC as well as CD27-AS1/GUSBP11/SNHG16/TTC28-AS1-miR-22-3p-FABP5 ceRNA regulatory network in LIHC. Among those FABP5-related regulatory networks, the association between miR-22-3p and FABP5 in LIHC got discovered and confirmed via miRNA-mRNA interaction prediction and experiments for the first time. Moreover, the potential relationships of FABP5 expression with immune cell infiltration and immune checkpoints in multiple tumor types were investigated. Our work not only helps to further understand functions of FABP5 and supplement existing FABP5-related mechanisms in tumors, but also provides more possibilities for immunotherapy.

## Methods

### Data source

TCGA (http://cancergenome.nih.gov) database is a cancer genomics program characterizing more than twenty thousand primary cancers and matching normal samples of 33 tumor types. Our study employed UCSC Xena (https://xenabrowser.net/), a browser interactively visualizing cancer genome datasets, to collect gene expression RNA sequence and clinical data across TCGA tumor types^57^. In several tumor types, less than five normal samples are included in TCGA database, so we analyzed the difference in FABP5 expression between tumor and normal tissues in these tumor types after adding normal samples from Genotype-Tissue Expression (GTEx, http://commonfund.nih.gov/GTEx/) database.

### Expression analysis

Perl script and R language were applied for data organization. Using R language, we made expression analysis of FABP5 in tumor types with at least five adjacent normal tissues in TCGA database. |log2FC| > 1 and the adjusted *p*-value < 0.05 were regarded as the cut-off criterion. As for those tumor types with less than five normal samples in TCGA database, we applied Gene Expression Profiling Interactive Analysis (GEPIA, http://gepia.cancer-pku.cn/) to compare FABP5 expression between tumor and normal tissues based on TCGA and GTEx database. Moreover, the “Stage Plot” module of GEPIA was applied to explore the correlations between the expression level of FABP5 and pathological stages of different tumors.

### Survival analysis

Using median FABP5 expression level as the cutoff, tumor samples were divided into the high-expression and low-expression groups. The “Survival Plots” module of GEPIA was applied to compare the OS and DFS between high-expression and low-expression group. Additionally, we used “survival” package in R to make survival analysis by the Kaplan–Meier survival curve and Log-rank test in tumor types that have at least five corresponding normal tissues in TCGA database. Only tumor types with statistically significant results of survival analysis by both Kaplan–Meier survival curve and Log-rank test will be chosen for further exploration of FABP5-related regulatory network. Moreover, we utilized “survival” and “survminer” packages in R to make survival analysis of predicted miRNAs and lncRNAs by Kaplan–Meier survival curve.

### Prediction of upstream miRNA and lncRNA

Encyclopedia of RNA Interactomes (ENCORI, http://starbase.sysu.edu.cn/), a database that integrates RNA interactions, was applied to predict miRNA-mRNA and lncRNA-miRNA interactions^58^. All interactions obtained by ENCORI have been supported by Ago CLIP-seq data. Based on the positive results of at least one miRNA-target predicting databases including microT, miRanda, miRmap, PicTar, PITA, RNA22 and TargetScan, FABP5-related miRNAs were identified. Besides, upstream lncRNAs of FABP5-related miRNAs were predicted by iRanda program.

### Analysis of immune infiltration and immune checkpoints

Tumor Immune Estimation Resource Version 2 (TIMER2.0, http://timer.comp-genomics.org/) provides comprehensive analyses of immune infiltrates and visualizes their functions in multiple tumor types^59^. In this study, TIMER2.0 was used for the evaluation of relationships between FABP5 expression and immune infiltration (B cells, CD4+ T-cells, CD8+ T-cells, DCs, macrophages, Tregs and CAFs) across TCGA tumor types. Besides, the “gene correlation” module of TIMER2.0 was used to explore associations between FABP5 and immune checkpoints (CD274, PDCD1, CTLA-4, LAG-3, HAVCR2 and TIGIT). P-values and partial rho values were obtained by purity-adjusted Spearman’s rank correlation test.

### Correlation analysis

R language was applied for evaluating the relationships between FABP5 and FABP5-bound miRNAs, FAPB5-bound miRNAs and corresponding lncRNAs. Spearman’s rank correlation test was used to obtain the p-values and partial rho values.

### Cell culture

Two different hepatocarcinoma cell lines (Hep3B and HepG2) were respectively cultured with their own special mediums (Procell, China) in a 37 ℃ incubator with 5% CO_2_. Preparing for the subsequent transfection assay, cells were plated into 24 mm six-well plates at a density of 3 × 10^5^ cell/ml and cultured for 24 h.

### Transfection assay

MicroRNA-22-3p mimics, nonspecific miR-NC oligo and the transfection reagent were obtained from GenePharma (Shanghai, China). Hepatocarcinoma cells in each six-well plate were divided into two groups, miR NC group and miR-22-3p mimics group. Using the transfection reagent, miR NC group got transfected with miR-NC, and miR-22-3p mimics group got transfected with miR-22-3p mimics. All cells after treatments were cultured at 37 ℃, 5% CO_2_ for 72 h, and then collected for further analysis. Specific sequences were as follows: miR-22-3p mimics sense: 5′-3′AAGCUGCCAGUUGAAGAACUGU and antisense: 5′-3′AGUUCUUCAACUGGCAGCUUUU. miR-NC sense: 5′-3′UUCUCCGAACGUGUCACGUTT and antisense: 5′-3′ACGUGACACGUUCGGAGAATT.

### RT-qPCR

Total RNA from cultured cells of different groups was obtained by Ezol reagent (GenePharma, China), and purified via column passing method. Reverse transcription and quantitative real-time PCR (qPCR) were conducted via Hairpin-it™ microRNA and U6 snRNA Normalization RT-PCR Quantitation Kit (GenePharma, China) according to the manufacturer's instructions. The primer sequences for RT-qPCR were as follows: HmiR-22-3p-FO-2: 5′-3′GCGGTCAAGCTGCCAGTT and HmiR-22-3p-RE-2: 5′-3′TATGGTTGTTCACGACTCCTTCAC. U6 forward: 5′-3′CGCTTCGGCAGCACATATAC and reverse: 5′-3′TTCACGAATTTGCGTGTCATC.

### Western blot

Total protein from cultured cells of different groups was extracted by using Whole Protein Extraction Kit (Solarbio Life Science, China) according to the manufacturer's instructions. Then, we measured the protein concentration by using the bicinchoninic acid (BCA) protein assay kit (Elabscience, China). According to the molecular weight of FABP5, 12.5% SDS-PAGE gels were prepared by using Omni-Easy™ One-Step PAGE Gel Fast Preparation Kit (Yamay, China) for the separation of proteins. After protein separation, we transferred proteins onto PVDF membranes, and blocked membranes with QuickBlock™ Blocking Buffer (Beyotime, China). Then, membranes got incubated with corresponding primary antibodies, including antibodies against FABP5 (1:1000, Biodragon, China) and β-actin (1:20,000, ABclonal, China), at 4 ℃ overnight. We further incubated membranes with secondary antibodies (1:1000, Beyotime, China) for 2 h after washing them. After treated by ECL chemiluminescence method, the bands of the membranes got revealed.

## Supplementary Information

Supplementary Information.

## Data Availability

Publicly available datasets were analyzed in this study, these can be found in UCSC Xena (https://xenabrowser.net/). The ID numbers included: TCGA-BLCA.htseq_fpkm.tsv, TCGA-BRCA. htseq_fpkm.tsv, TCGA-CHOL.htseq_fpkm.tsv, TCGA-COAD.htseq_fpkm.tsv, TCGA-ESCA.htseq_fpkm.tsv, TCGA-GBM.htseq_fpkm.tsv, TCGA-HNSC. htseq_fpkm.tsv, TCGA-KICH.htseq_fpkm.tsv, TCGA-KIRC.htseq_fpkm.tsv, TCGA-KIRP.htseq_fpkm.tsv, TCGA-LIHC.htseq_fpkm.tsv, TCGA-LUAD. htseq_fpkm.tsv, TCGA-LUSC.htseq_fpkm.tsv, TCGA-PRAD.htseq_fpkm.tsv, TCGA-READ.htseq_fpkm.tsv, TCGA-STAD.htseq_fpkm.tsv, TCGA-THCA. htseq_fpkm.tsv, TCGA-UCEC.htseq_fpkm.tsv. Some of the data were stored in the Supplementary Data. The authors confirm that the data supporting the findings of this study are available within the article and its Supplementary materials.
