# Supplementary figures and images for: Oncogenic role and potential regulatory mechanism of fatty acid binding protein 5 based on a pan-cancer analysis
Source: Sci Rep. 2023 Mar 11;13:4060. doi: 10.1038/s41598-023-30695-9 (PMC10008585; doi:10.1038/s41598-023-30695-9)

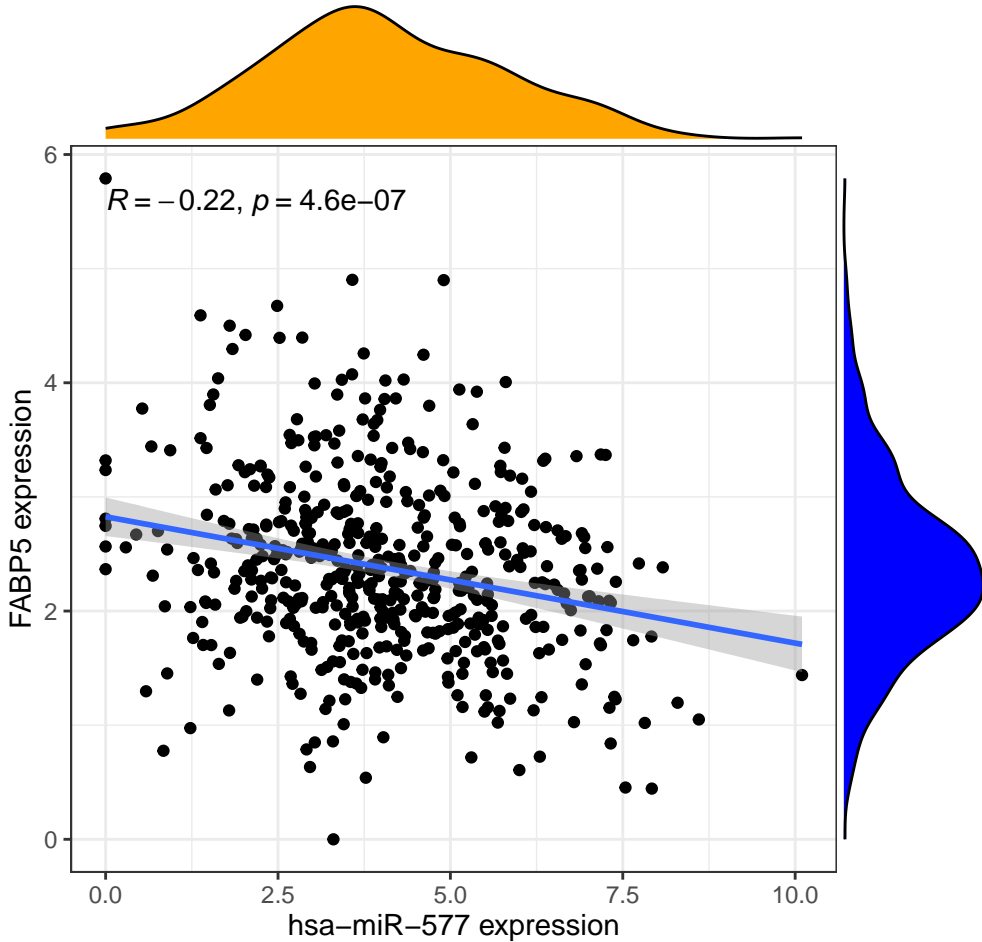

Supplement: Supplementary file 1 — Supplementary Information. [file 41598_2023_30695_MOESM1_ESM.zip › Supplementary Data/╩2╛▌/15 miRNA╧α╣╪╨╘/KIRC/cor.hsa-miR-577.pdf]

Type 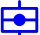 Normal 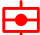 Tumor

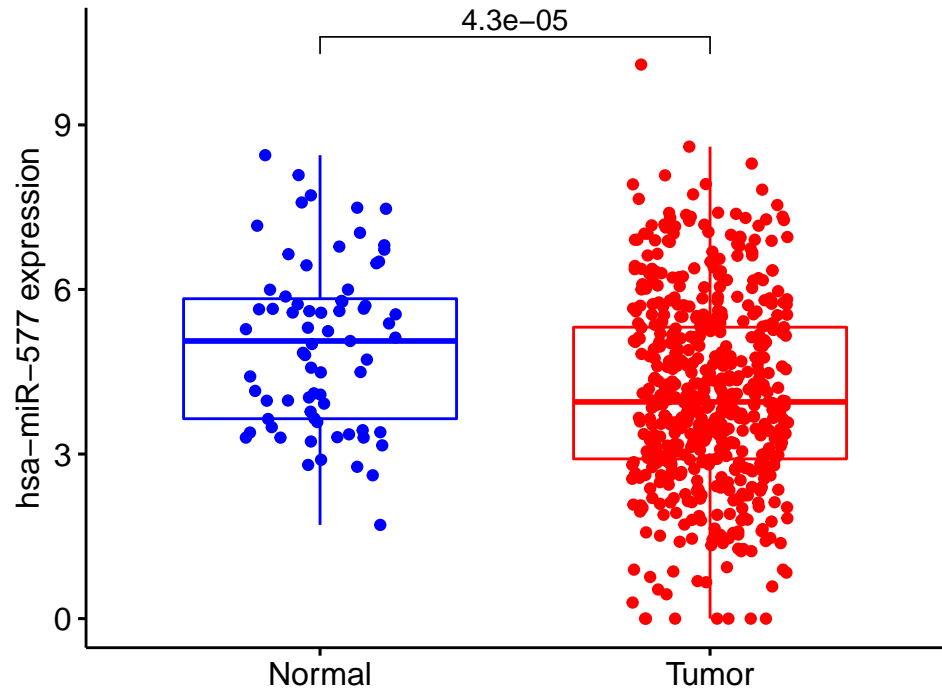

Supplement: Supplementary file 1 — Supplementary Information. [file 41598_2023_30695_MOESM1_ESM.zip › Supplementary Data/╩2╛▌/15 miRNA╧α╣╪╨╘/KIRC/diff.hsa-miR-577.pdf]

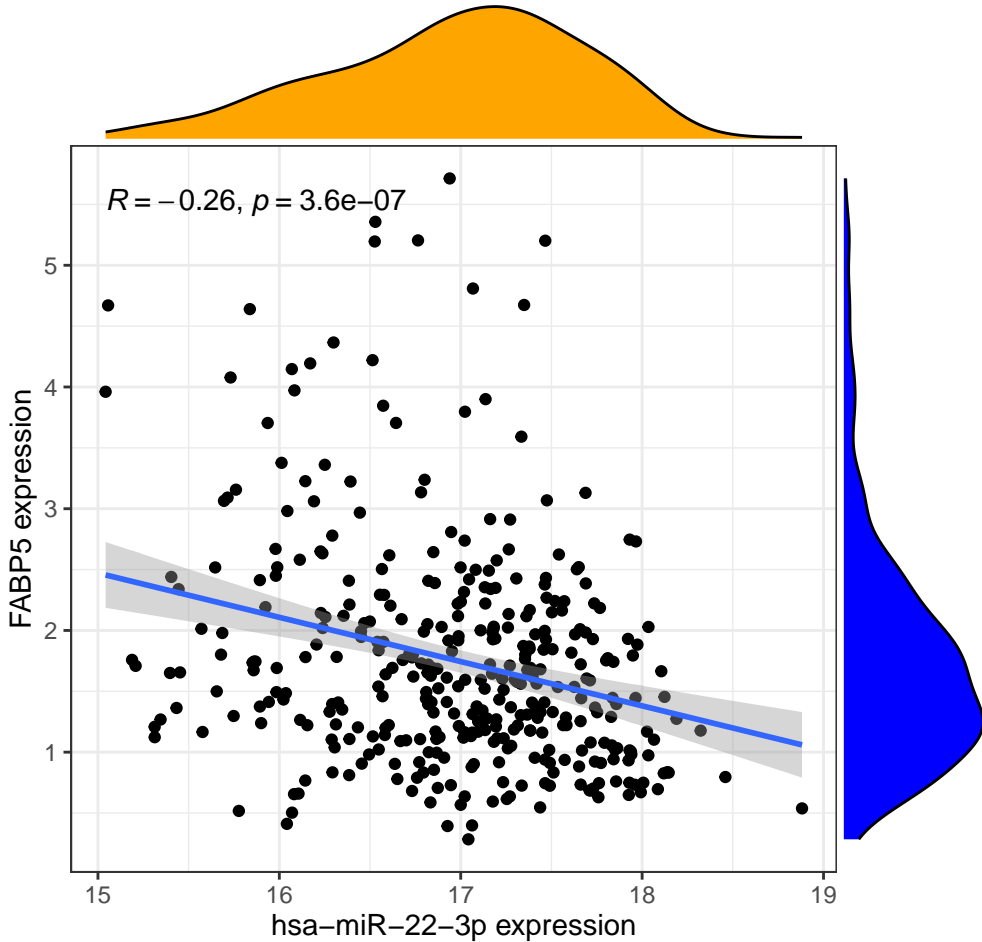

Supplement: Supplementary file 1 — Supplementary Information. [file 41598_2023_30695_MOESM1_ESM.zip › Supplementary Data/╩2╛▌/15 miRNA╧α╣╪╨╘/LIHC/cor.hsa-miR-22-3p.pdf]

Type 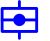 Normal 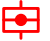 Tumor

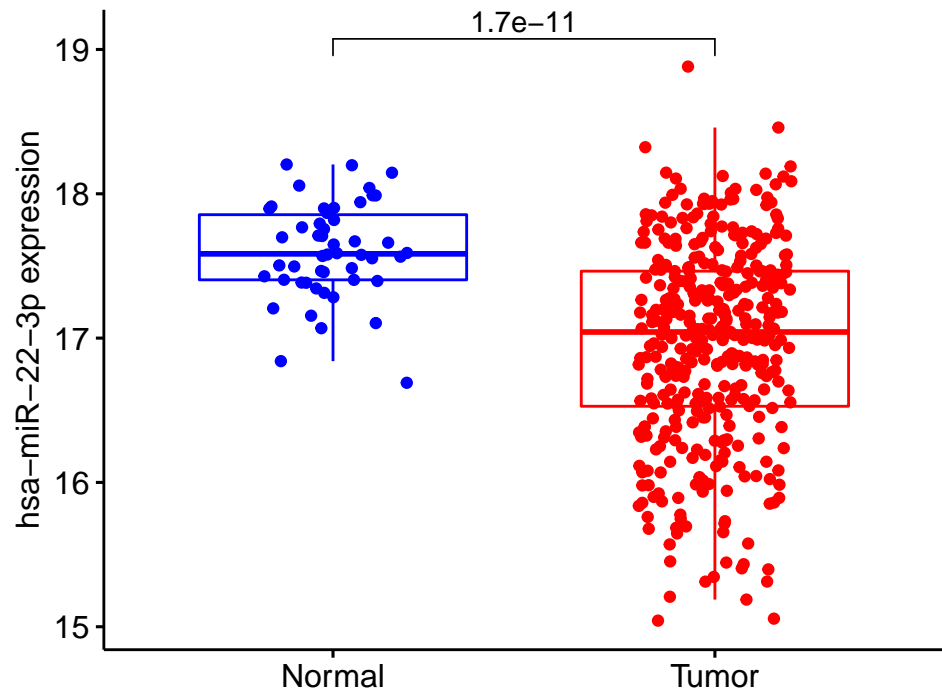

Supplement: Supplementary file 1 — Supplementary Information. [file 41598_2023_30695_MOESM1_ESM.zip › Supplementary Data/╩2╛▌/15 miRNA╧α╣╪╨╘/LIHC/diff.hsa-miR-22-3p.pdf]

hsa-miR-577    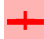 high    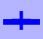 low

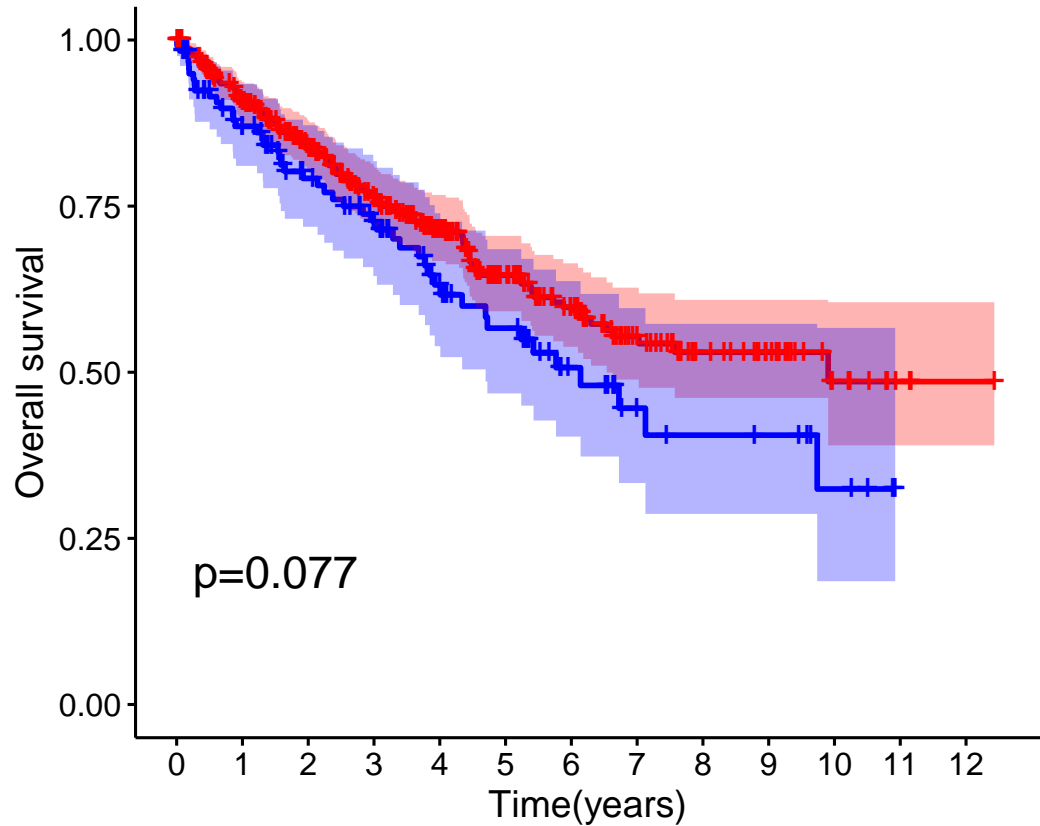

Supplement: Supplementary file 1 — Supplementary Information. [file 41598_2023_30695_MOESM1_ESM.zip › Supplementary Data/╩2╛▌/16 miRNA╔·┤μ╩▒╝Σ/KIRC/sur.hsa-miR-577.pdf]

hsa-miR-22-3p 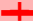 high 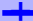 low

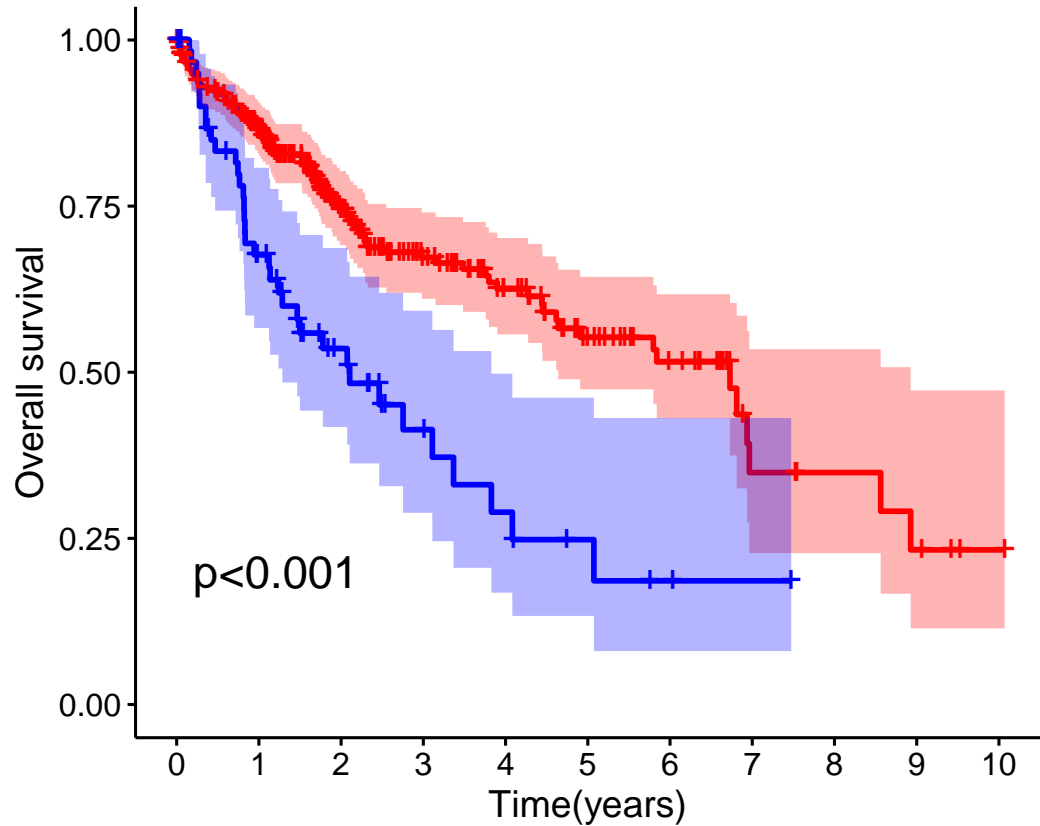

Supplement: Supplementary file 1 — Supplementary Information. [file 41598_2023_30695_MOESM1_ESM.zip › Supplementary Data/╩2╛▌/16 miRNA╔·┤μ╩▒╝Σ/LIHC/sur.hsa-miR-22-3p.pdf]

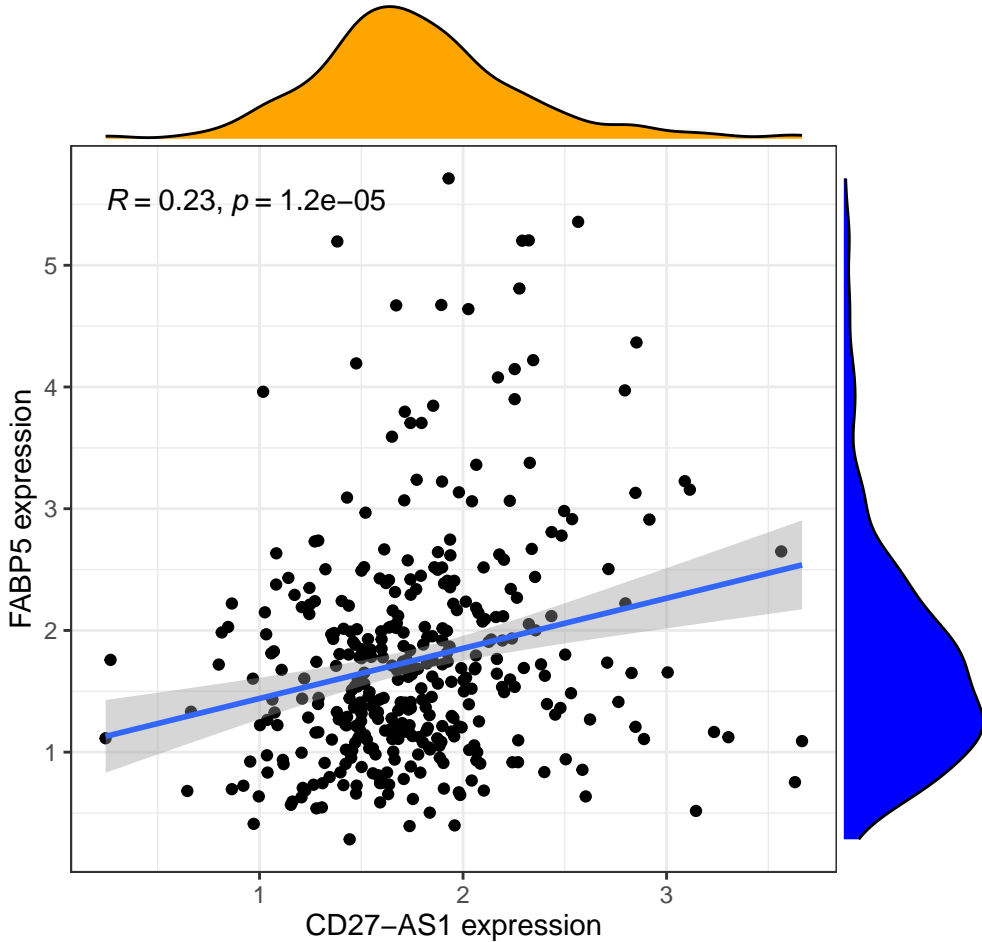

Supplement: Supplementary file 1 — Supplementary Information. [file 41598_2023_30695_MOESM1_ESM.zip › Supplementary Data/╩2╛▌/19 lncRNA╧α╣╪╨╘/LIHC_miR-22-3p/cor.FABP5_CD27-AS1.pdf]

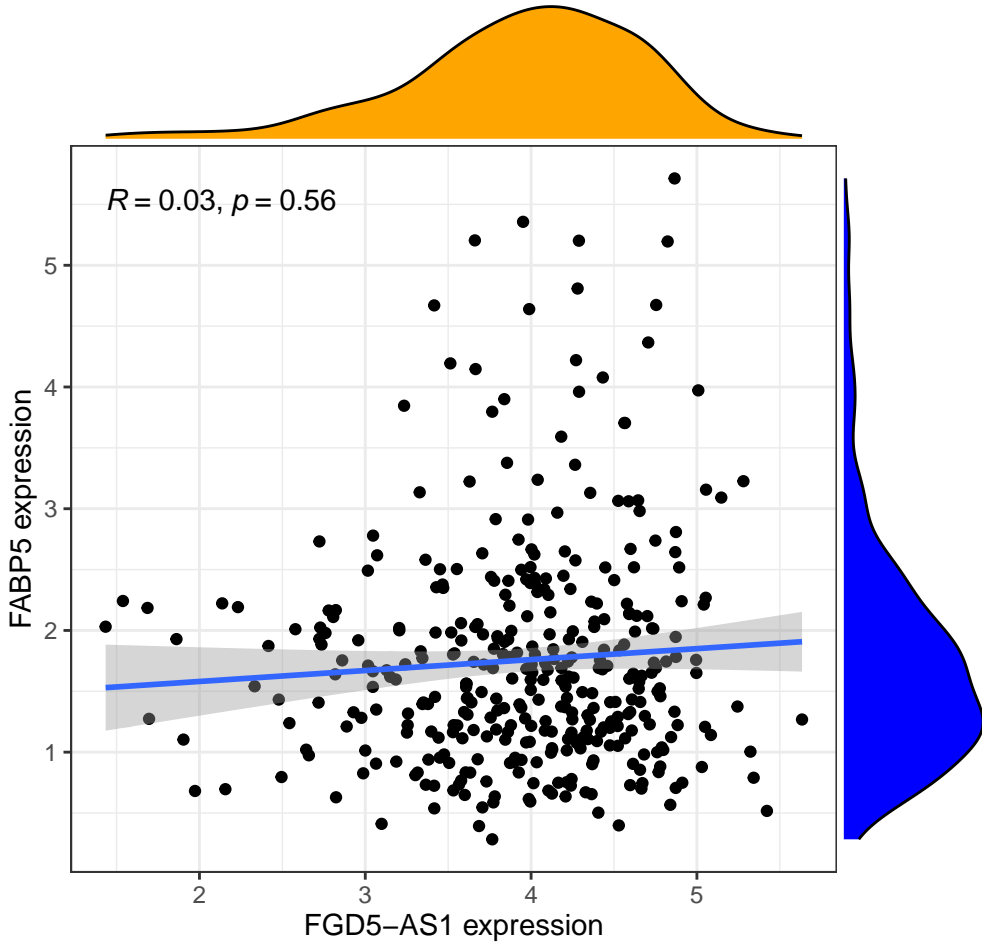

Supplement: Supplementary file 1 — Supplementary Information. [file 41598_2023_30695_MOESM1_ESM.zip › Supplementary Data/╩2╛▌/19 lncRNA╧α╣╪╨╘/LIHC_miR-22-3p/cor.FABP5_FGD5-AS1.pdf]

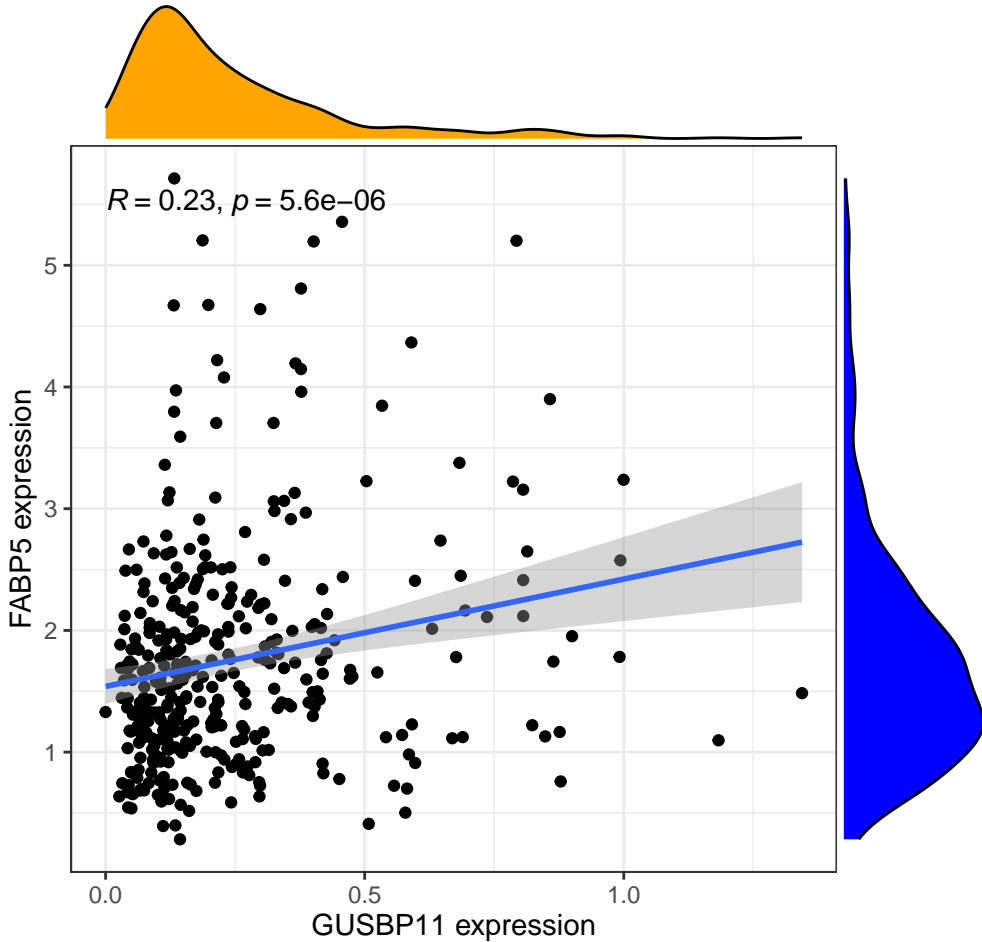

Supplement: Supplementary file 1 — Supplementary Information. [file 41598_2023_30695_MOESM1_ESM.zip › Supplementary Data/╩2╛▌/19 lncRNA╧α╣╪╨╘/LIHC_miR-22-3p/cor.FABP5_GUSBP11.pdf]

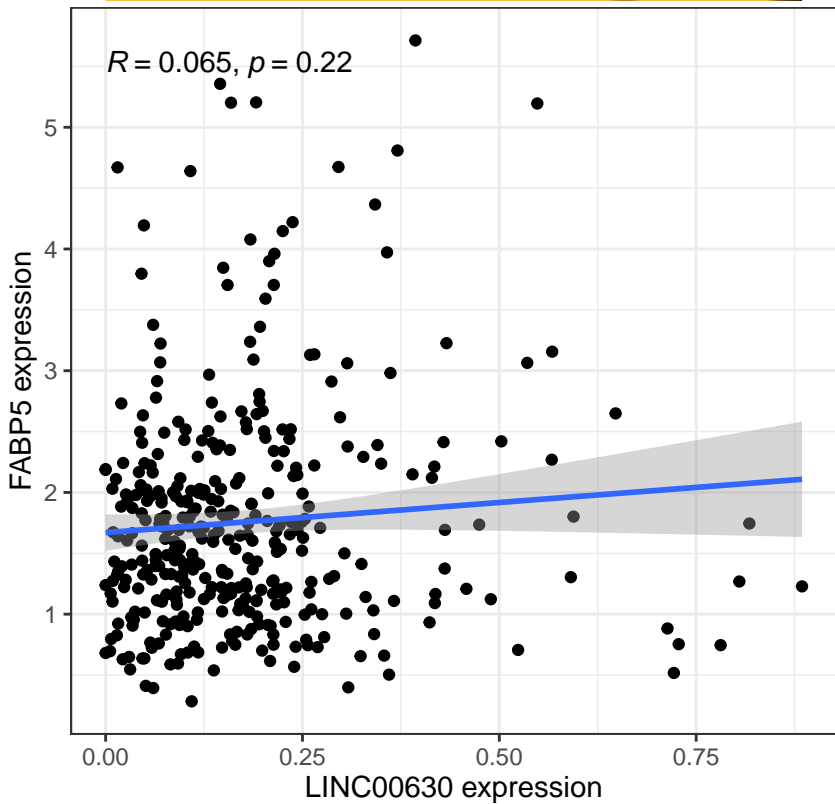

Supplement: Supplementary file 1 — Supplementary Information. [file 41598_2023_30695_MOESM1_ESM.zip › Supplementary Data/╩2╛▌/19 lncRNA╧α╣╪╨╘/LIHC_miR-22-3p/cor.FABP5_LINC00630.pdf]

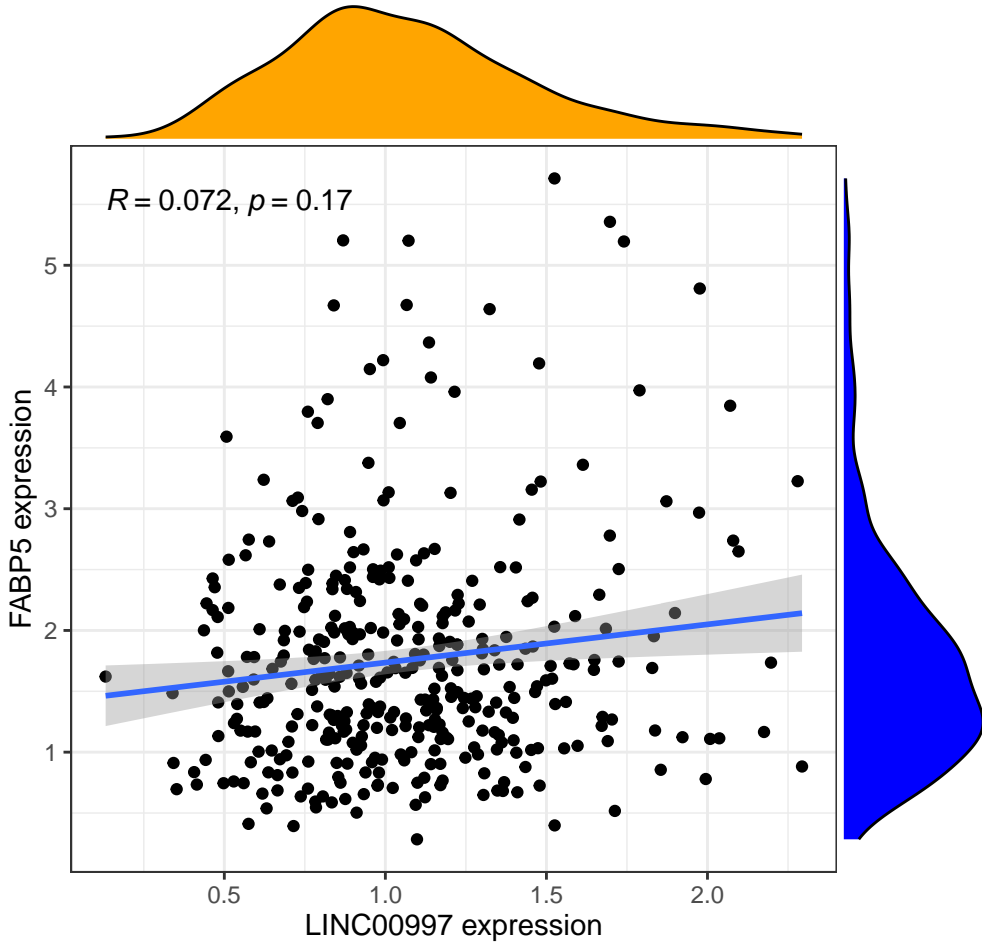

Supplement: Supplementary file 1 — Supplementary Information. [file 41598_2023_30695_MOESM1_ESM.zip › Supplementary Data/╩2╛▌/19 lncRNA╧α╣╪╨╘/LIHC_miR-22-3p/cor.FABP5_LINC00997.pdf]

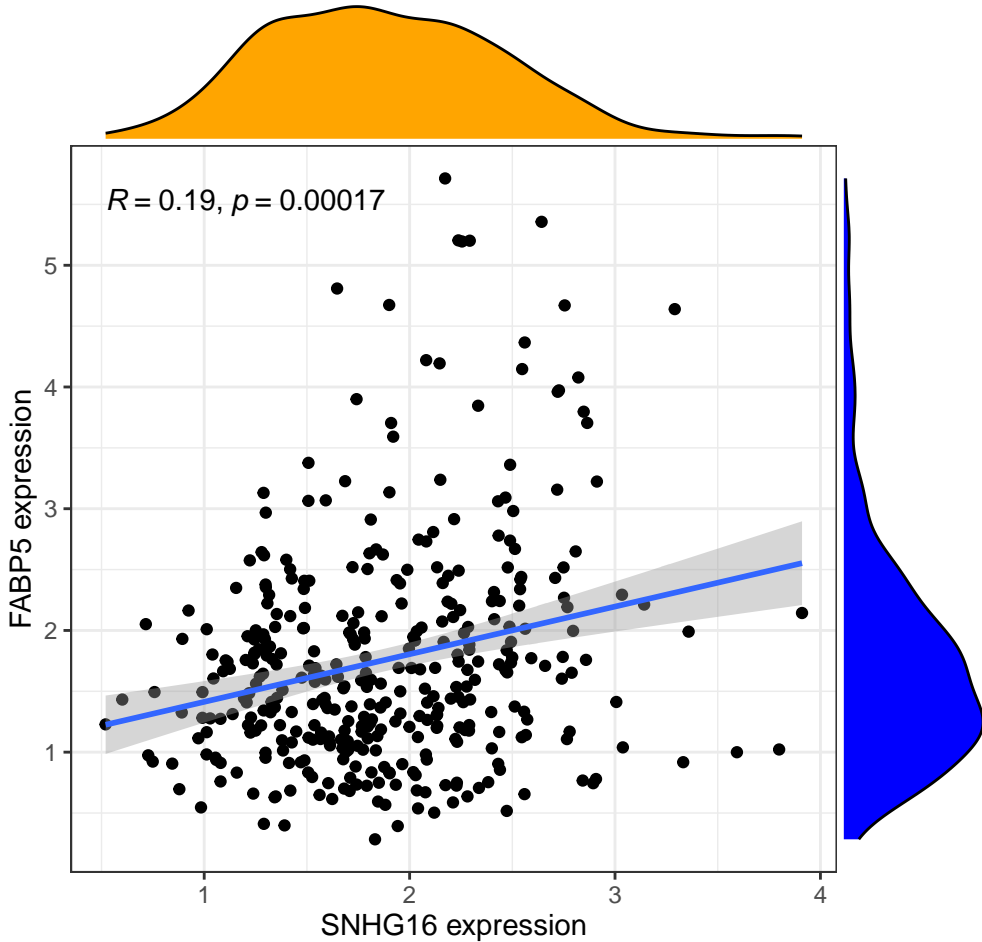

Supplement: Supplementary file 1 — Supplementary Information. [file 41598_2023_30695_MOESM1_ESM.zip › Supplementary Data/╩2╛▌/19 lncRNA╧α╣╪╨╘/LIHC_miR-22-3p/cor.FABP5_SNHG16.pdf]

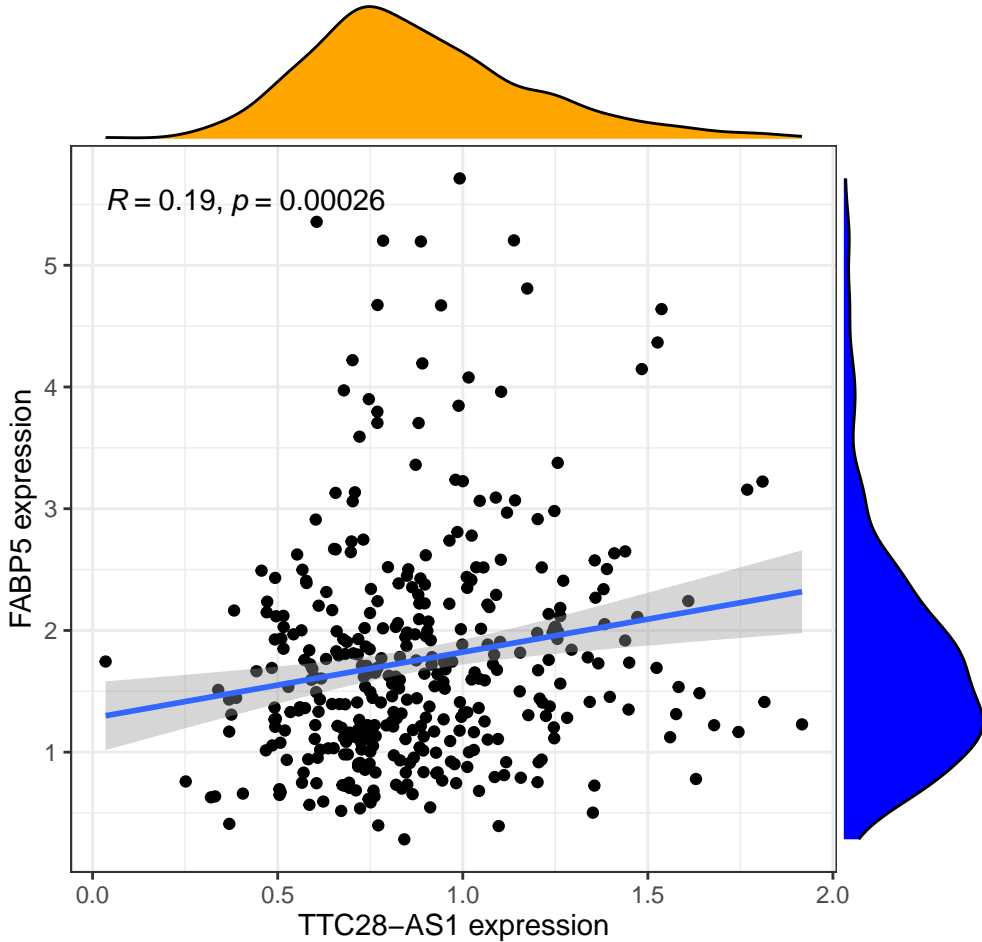

Supplement: Supplementary file 1 — Supplementary Information. [file 41598_2023_30695_MOESM1_ESM.zip › Supplementary Data/╩2╛▌/19 lncRNA╧α╣╪╨╘/LIHC_miR-22-3p/cor.FABP5_TTC28-AS1.pdf]

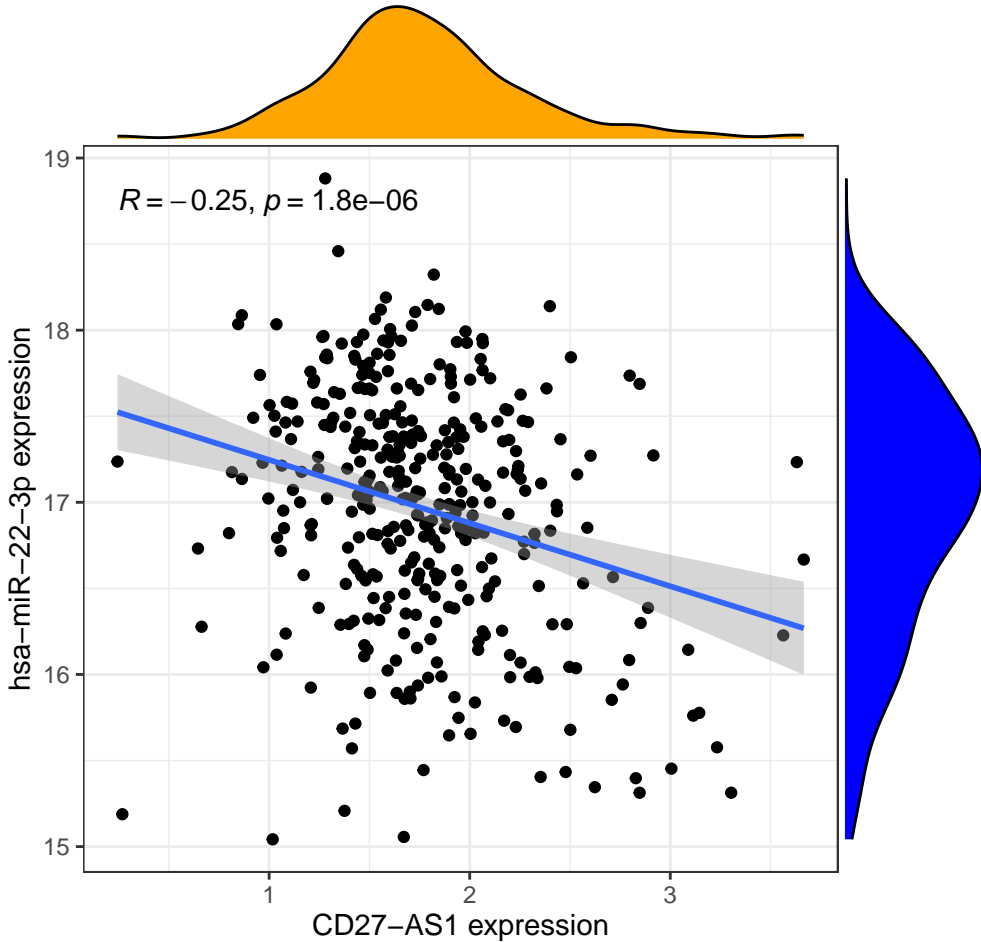

Supplement: Supplementary file 1 — Supplementary Information. [file 41598_2023_30695_MOESM1_ESM.zip › Supplementary Data/╩2╛▌/19 lncRNA╧α╣╪╨╘/LIHC_miR-22-3p/cor.hsa-miR-22-3p_CD27-AS1.pdf]

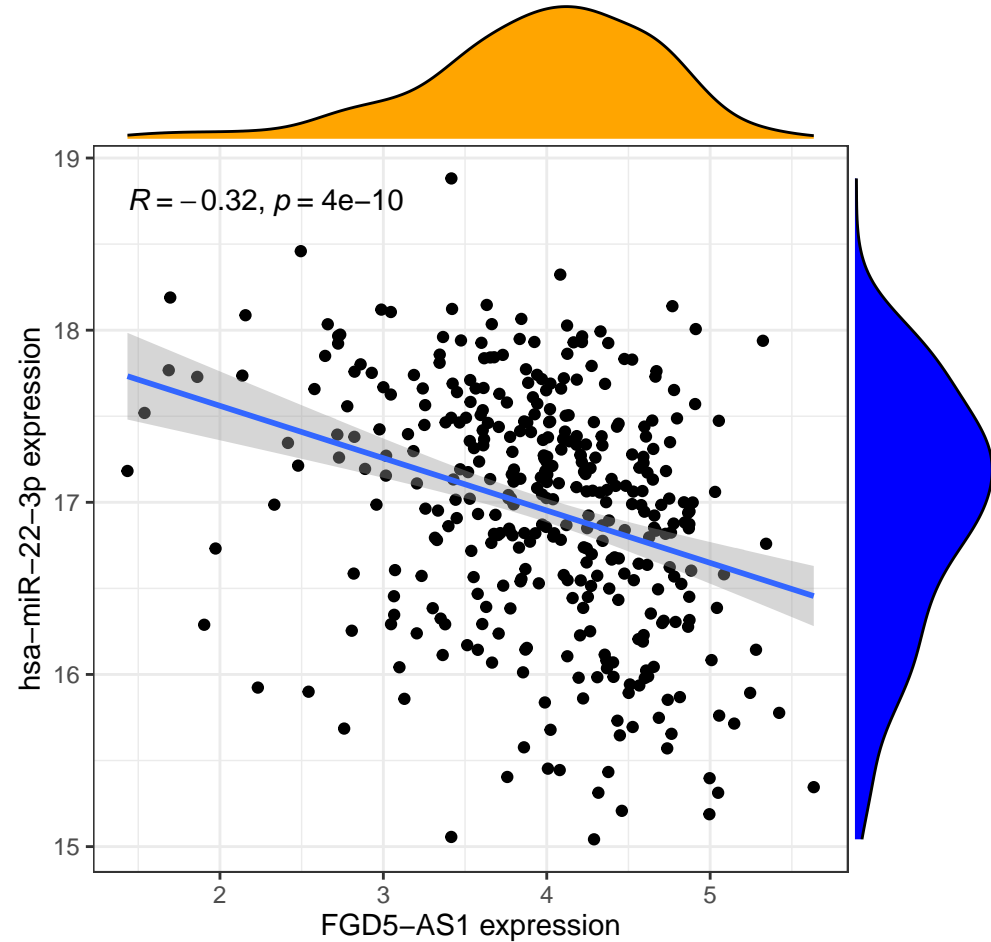

Supplement: Supplementary file 1 — Supplementary Information. [file 41598_2023_30695_MOESM1_ESM.zip › Supplementary Data/╩2╛▌/19 lncRNA╧α╣╪╨╘/LIHC_miR-22-3p/cor.hsa-miR-22-3p_FGD5-AS1.pdf]

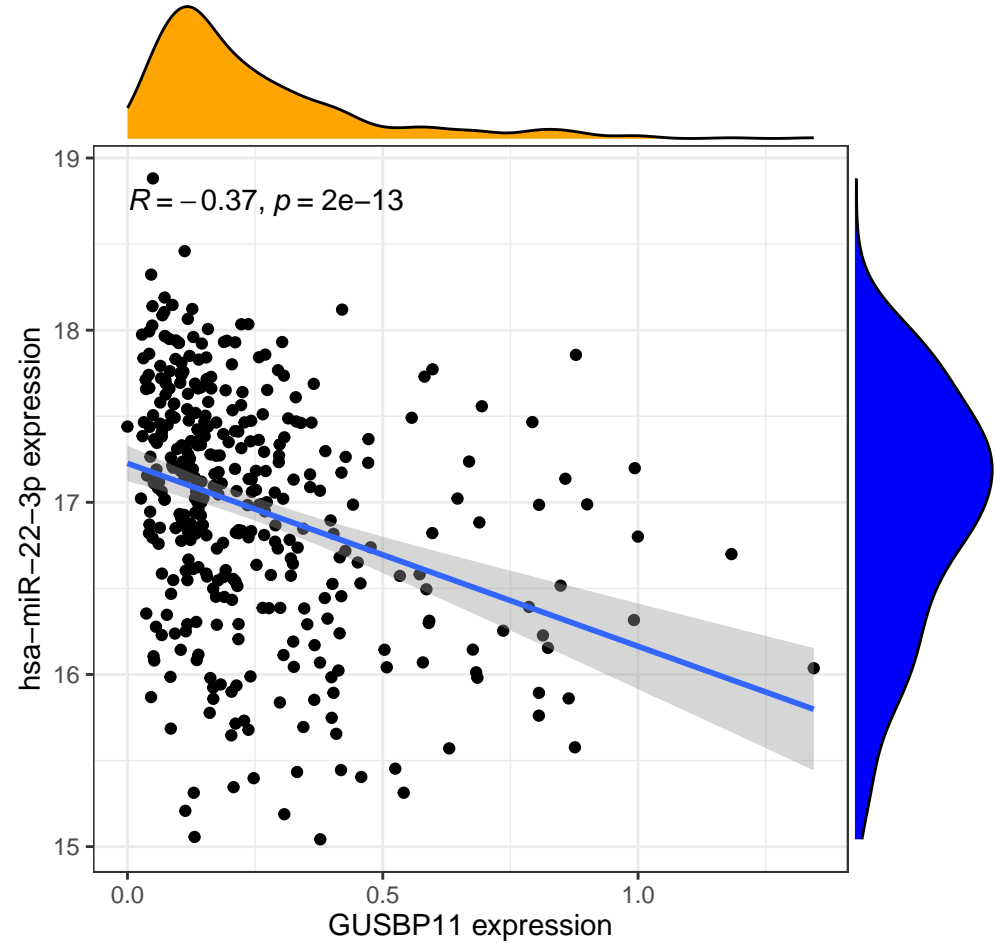

Supplement: Supplementary file 1 — Supplementary Information. [file 41598_2023_30695_MOESM1_ESM.zip › Supplementary Data/╩2╛▌/19 lncRNA╧α╣╪╨╘/LIHC_miR-22-3p/cor.hsa-miR-22-3p_GUSBP11.pdf]

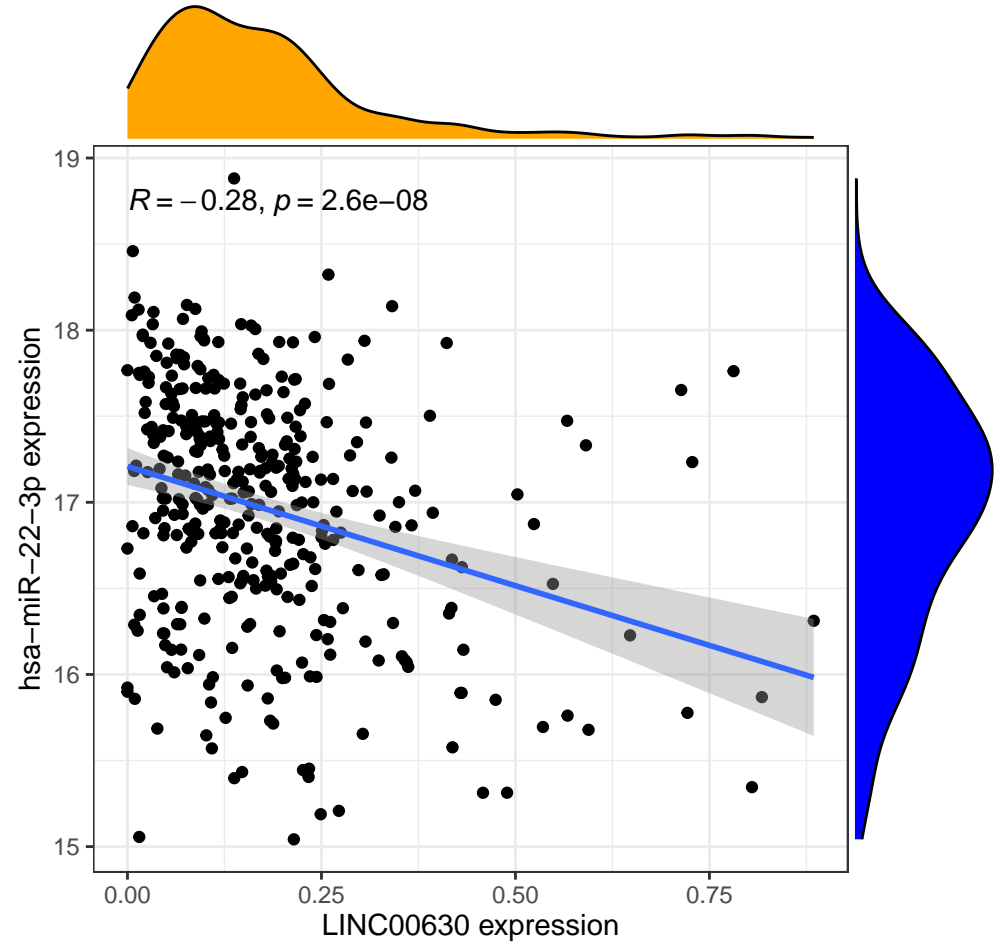

Supplement: Supplementary file 1 — Supplementary Information. [file 41598_2023_30695_MOESM1_ESM.zip › Supplementary Data/╩2╛▌/19 lncRNA╧α╣╪╨╘/LIHC_miR-22-3p/cor.hsa-miR-22-3p_LINC00630.pdf]

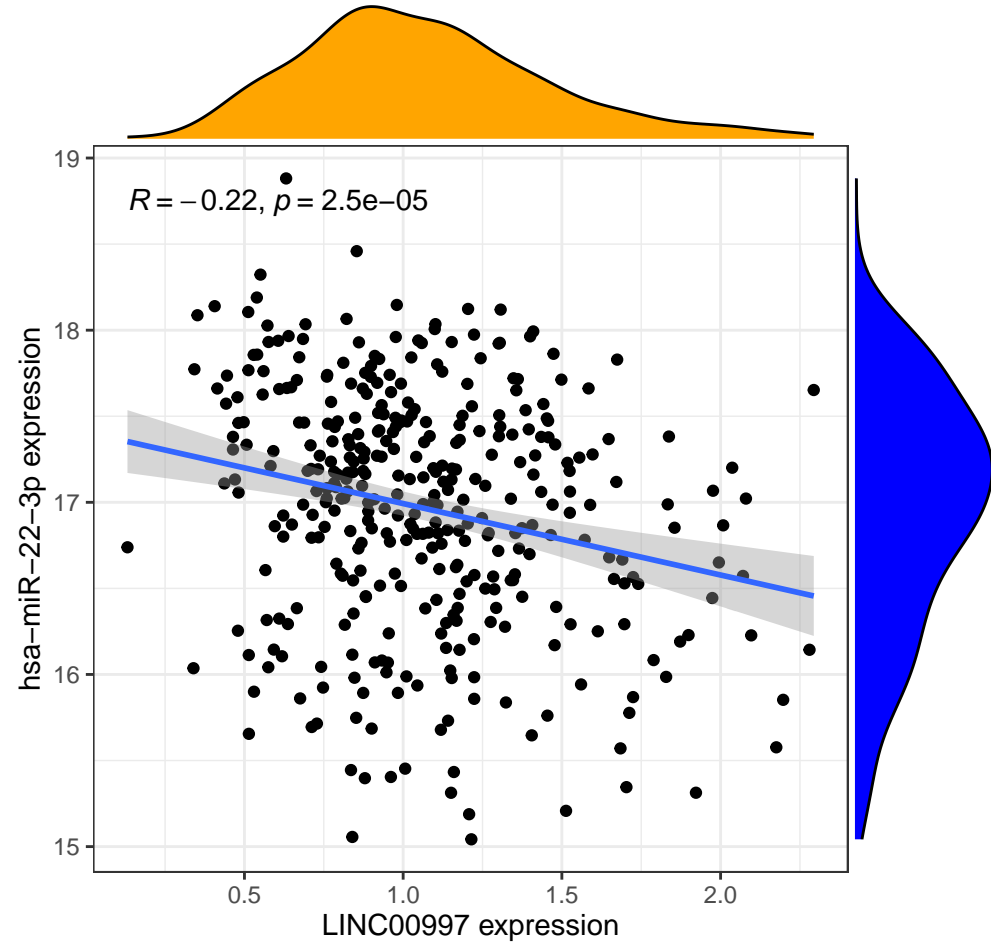

Supplement: Supplementary file 1 — Supplementary Information. [file 41598_2023_30695_MOESM1_ESM.zip › Supplementary Data/╩2╛▌/19 lncRNA╧α╣╪╨╘/LIHC_miR-22-3p/cor.hsa-miR-22-3p_LINC00997.pdf]

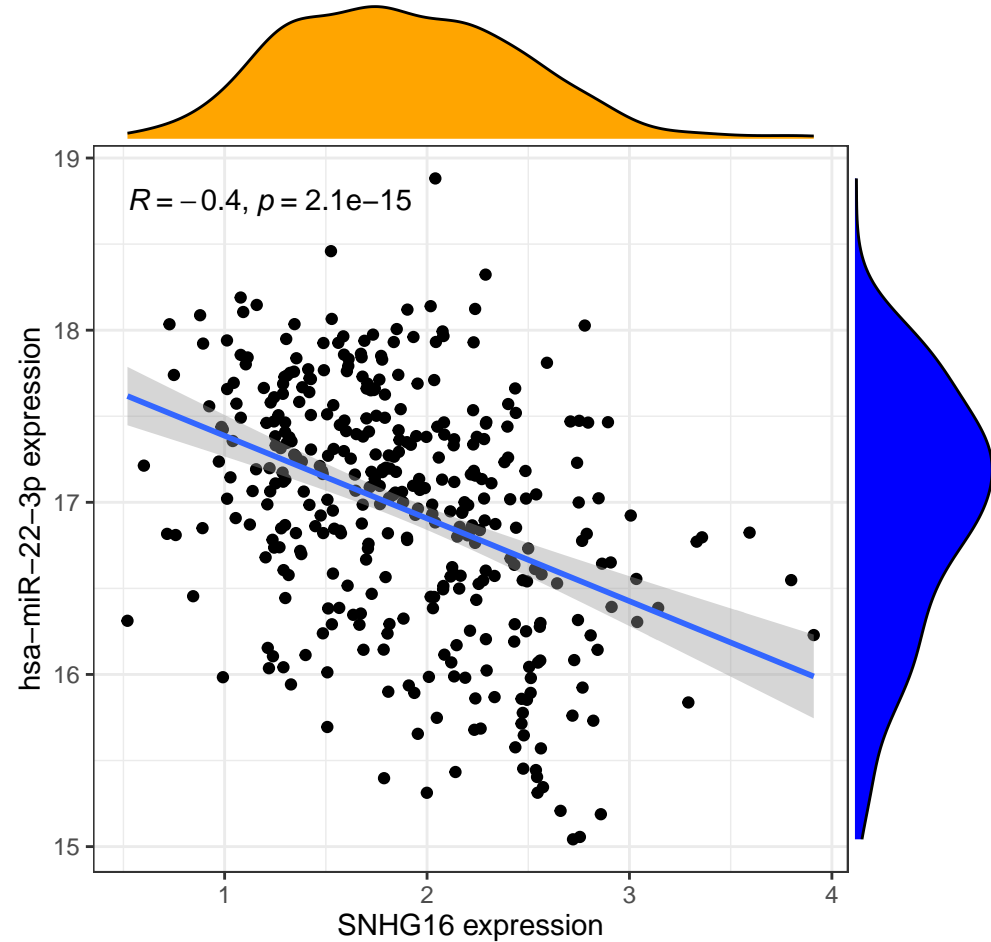

Supplement: Supplementary file 1 — Supplementary Information. [file 41598_2023_30695_MOESM1_ESM.zip › Supplementary Data/╩2╛▌/19 lncRNA╧α╣╪╨╘/LIHC_miR-22-3p/cor.hsa-miR-22-3p_SNHG16.pdf]

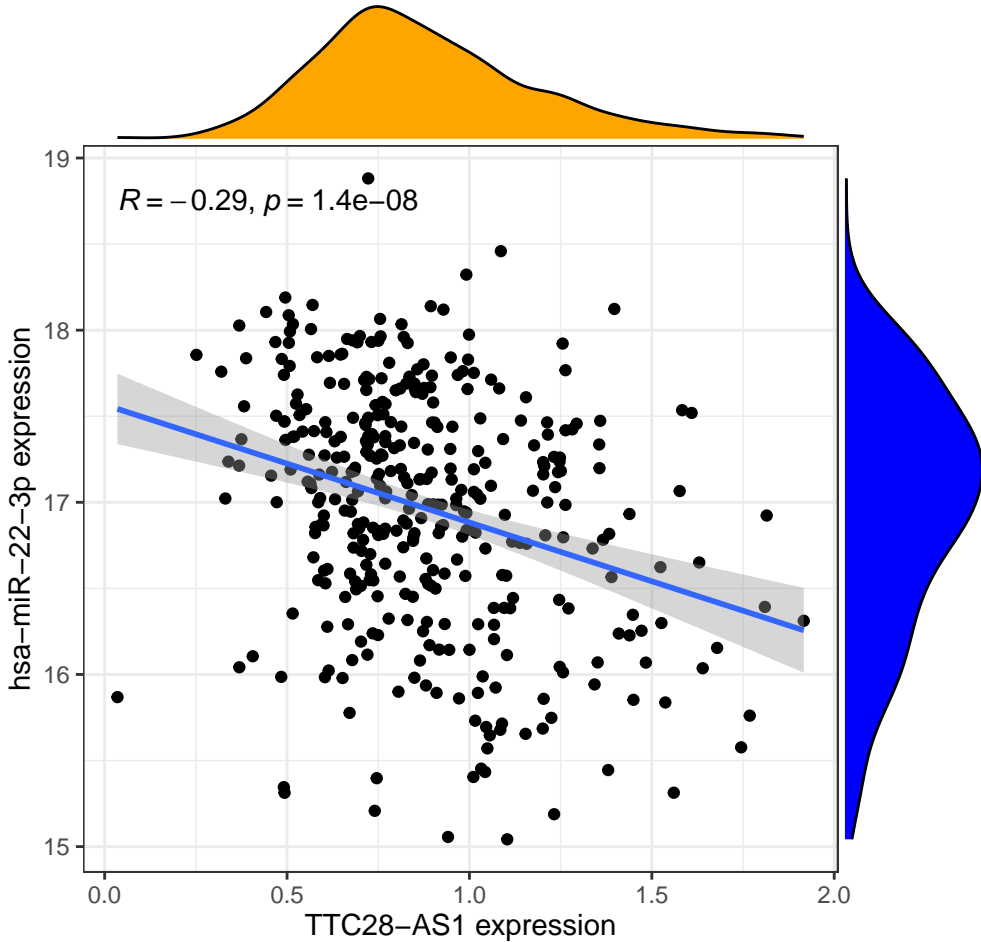

Supplement: Supplementary file 1 — Supplementary Information. [file 41598_2023_30695_MOESM1_ESM.zip › Supplementary Data/╩2╛▌/19 lncRNA╧α╣╪╨╘/LIHC_miR-22-3p/cor.hsa-miR-22-3p_TTC28-AS1.pdf]

Type 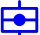 Normal 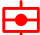 Tumor

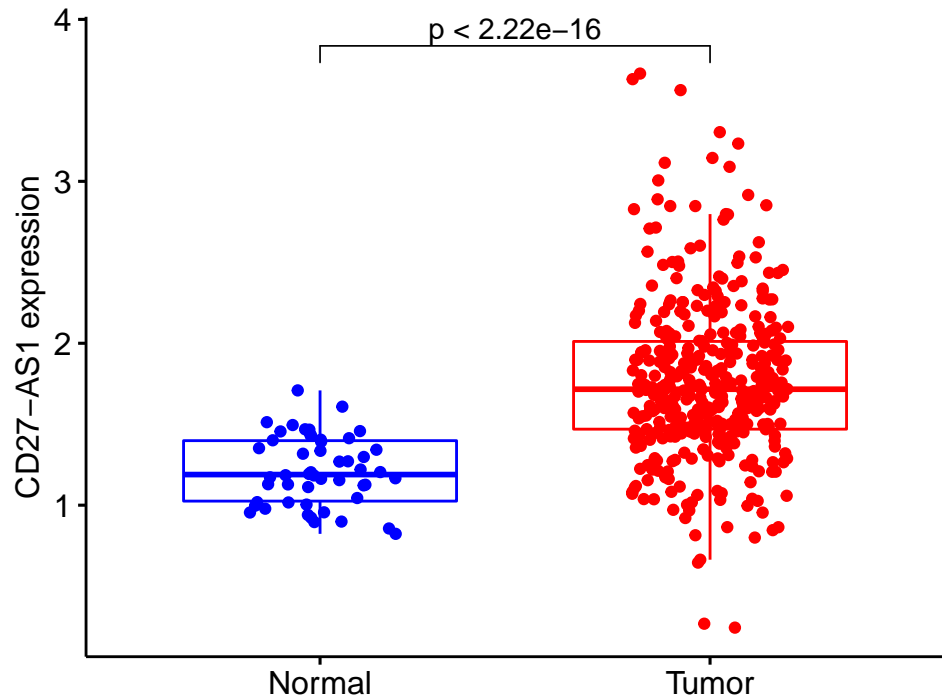

Supplement: Supplementary file 1 — Supplementary Information. [file 41598_2023_30695_MOESM1_ESM.zip › Supplementary Data/╩2╛▌/19 lncRNA╧α╣╪╨╘/LIHC_miR-22-3p/diff.CD27-AS1.pdf]

Type 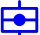 Normal 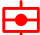 Tumor

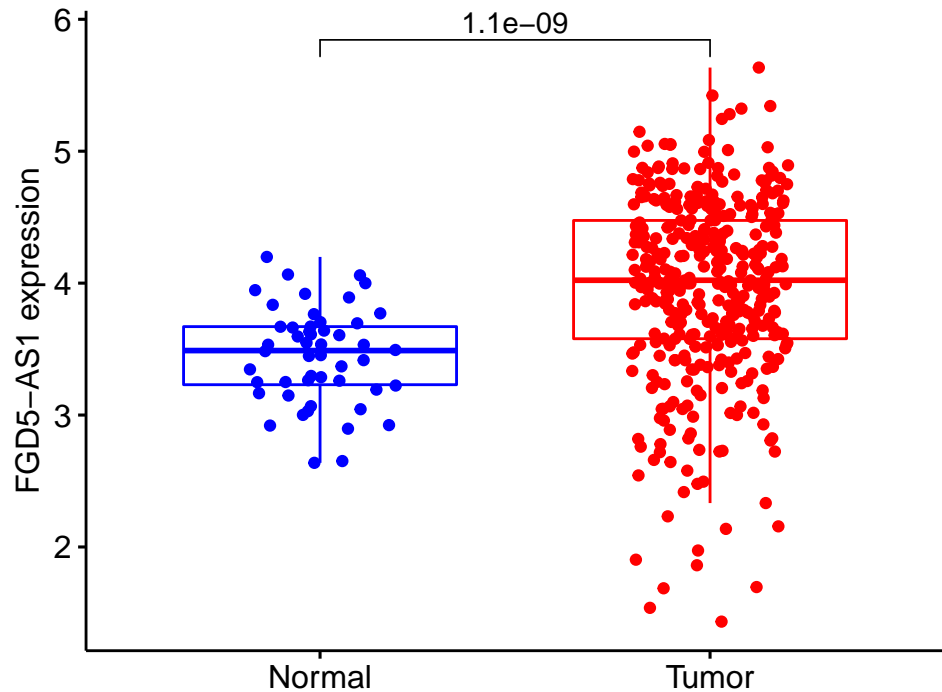

Supplement: Supplementary file 1 — Supplementary Information. [file 41598_2023_30695_MOESM1_ESM.zip › Supplementary Data/╩2╛▌/19 lncRNA╧α╣╪╨╘/LIHC_miR-22-3p/diff.FGD5-AS1.pdf]

Type 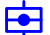 Normal 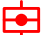 Tumor

$p < 2.22\text{e-}16$

GUSBP11 expression

0.0

0.5

1.0

Normal

Tumor

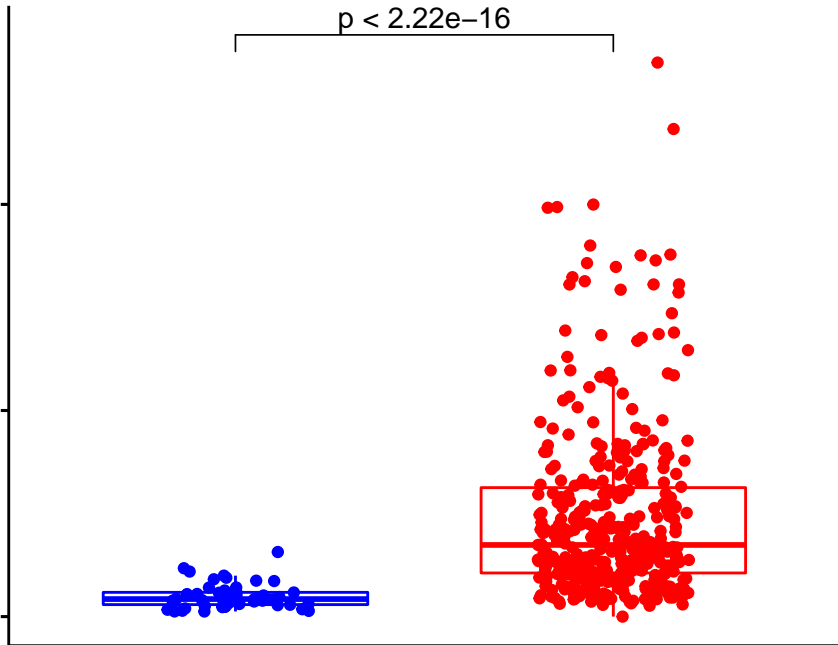

Supplement: Supplementary file 1 — Supplementary Information. [file 41598_2023_30695_MOESM1_ESM.zip › Supplementary Data/╩2╛▌/19 lncRNA╧α╣╪╨╘/LIHC_miR-22-3p/diff.GUSBP11.pdf]

Type 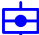 Normal 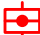 Tumor

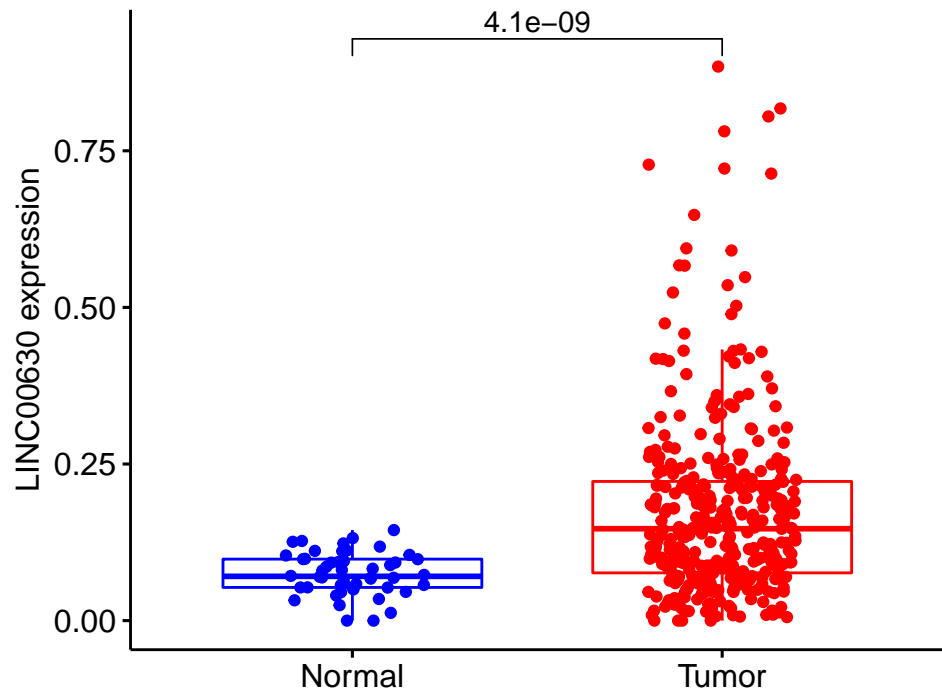

Supplement: Supplementary file 1 — Supplementary Information. [file 41598_2023_30695_MOESM1_ESM.zip › Supplementary Data/╩2╛▌/19 lncRNA╧α╣╪╨╘/LIHC_miR-22-3p/diff.LINC00630.pdf]

Type 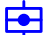 Normal 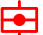 Tumor

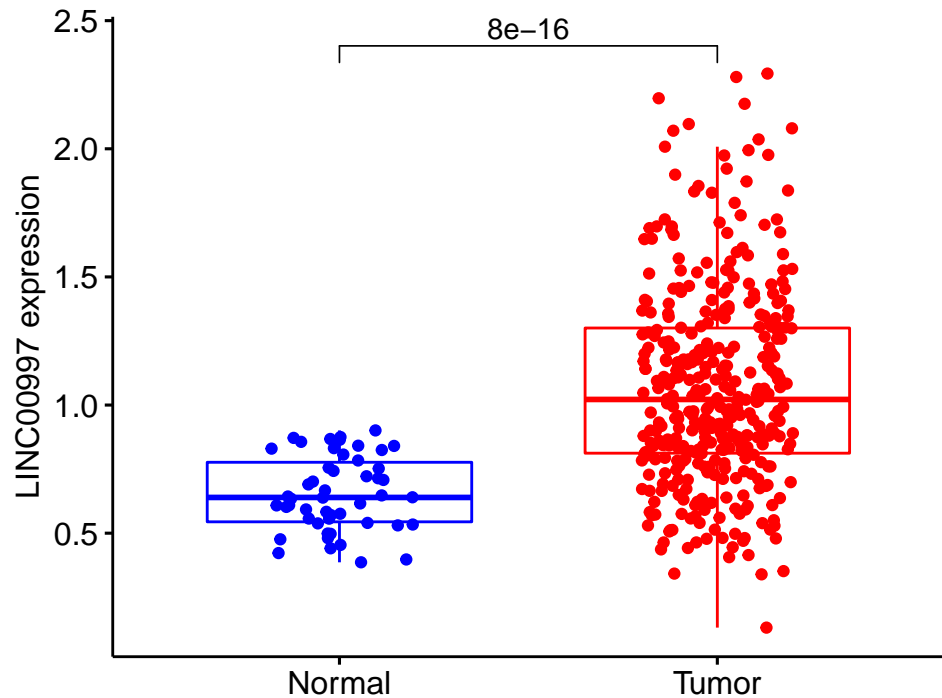

Supplement: Supplementary file 1 — Supplementary Information. [file 41598_2023_30695_MOESM1_ESM.zip › Supplementary Data/╩2╛▌/19 lncRNA╧α╣╪╨╘/LIHC_miR-22-3p/diff.LINC00997.pdf]

Type 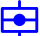 Normal 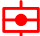 Tumor

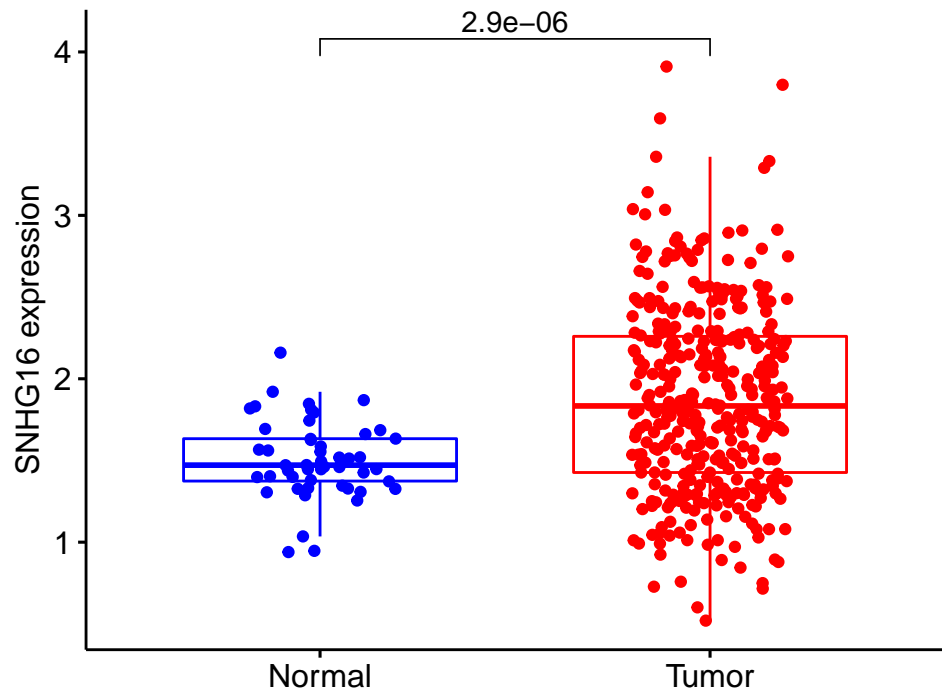

Supplement: Supplementary file 1 — Supplementary Information. [file 41598_2023_30695_MOESM1_ESM.zip › Supplementary Data/╩2╛▌/19 lncRNA╧α╣╪╨╘/LIHC_miR-22-3p/diff.SNHG16.pdf]

Type    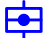 Normal    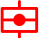 Tumor

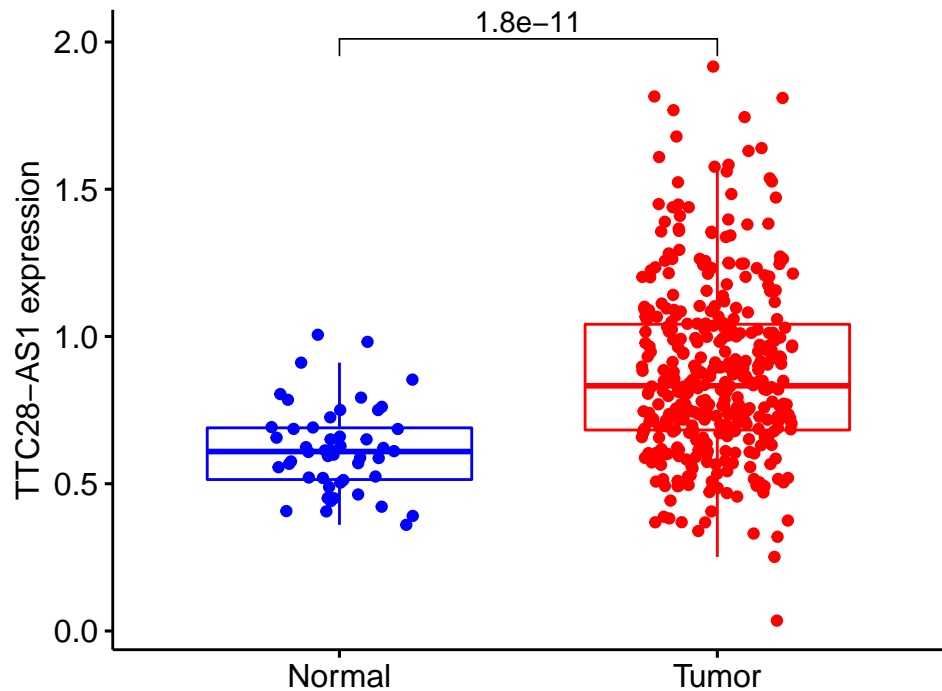

Supplement: Supplementary file 1 — Supplementary Information. [file 41598_2023_30695_MOESM1_ESM.zip › Supplementary Data/╩2╛▌/19 lncRNA╧α╣╪╨╘/LIHC_miR-22-3p/diff.TTC28-AS1.pdf]

CD27-AS1    high    low

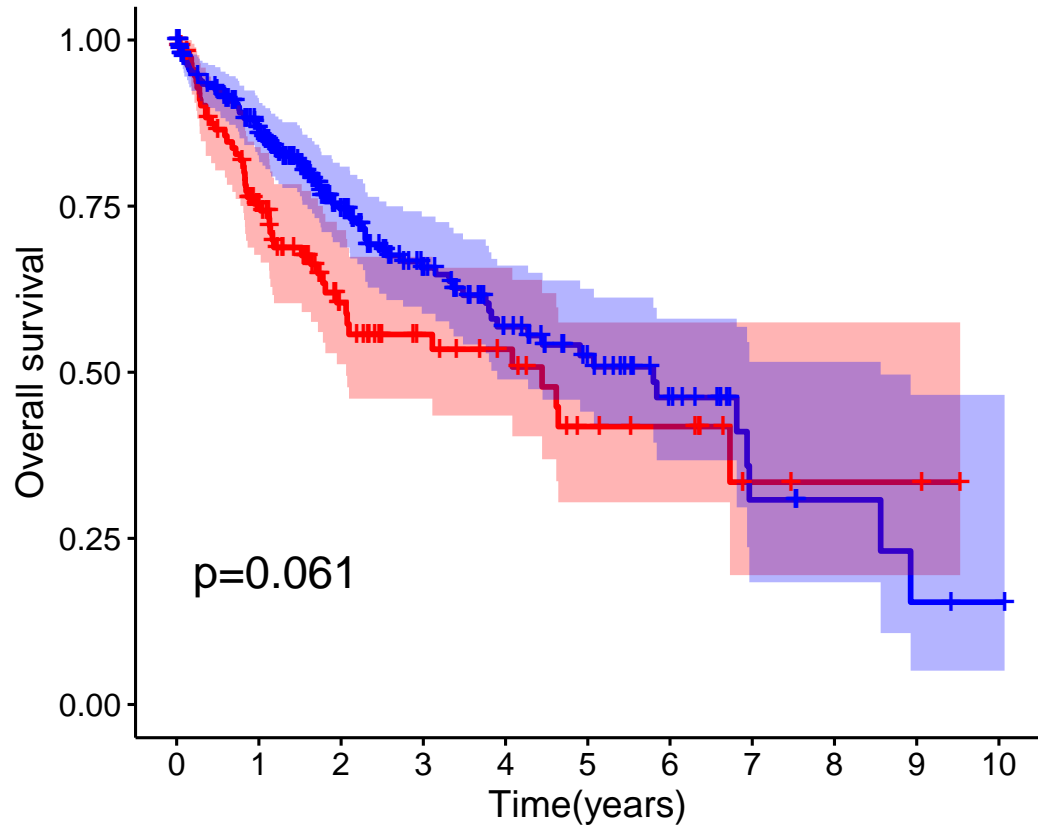

Supplement: Supplementary file 1 — Supplementary Information. [file 41598_2023_30695_MOESM1_ESM.zip › Supplementary Data/╩2╛▌/20 lncRNA╔·┤μ/sur.CD27-AS1 p=0.061.pdf]

FGD5-AS1 high low

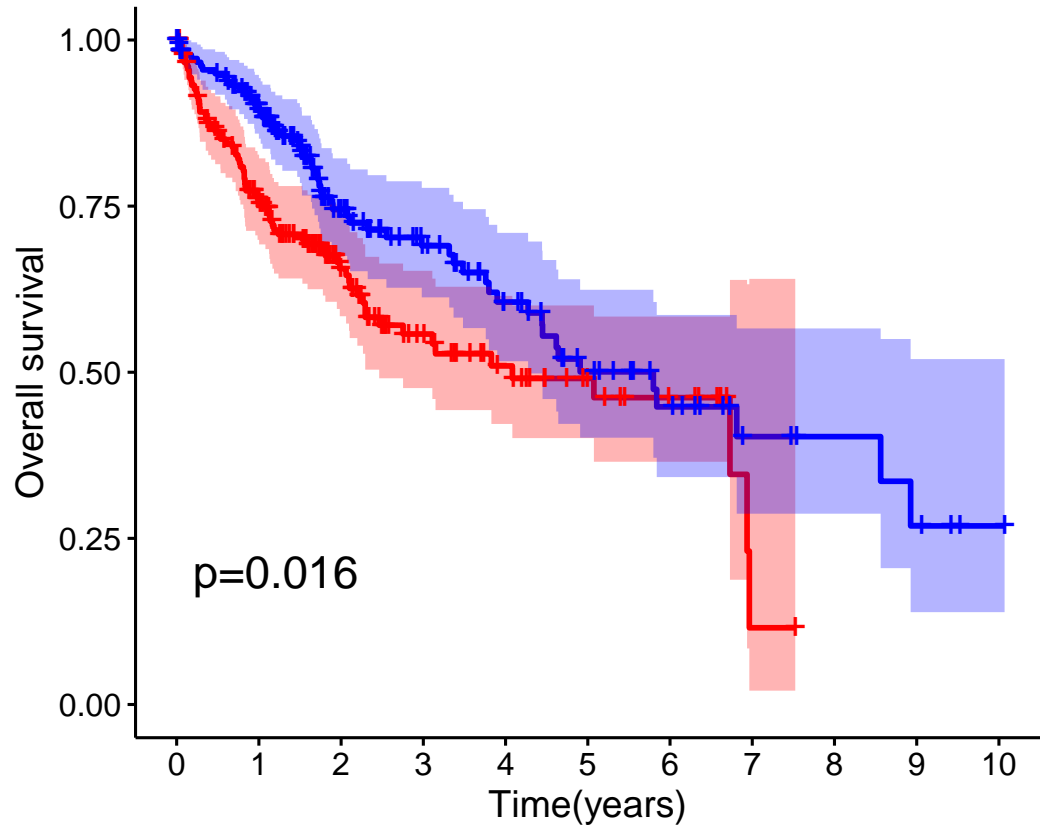

Supplement: Supplementary file 1 — Supplementary Information. [file 41598_2023_30695_MOESM1_ESM.zip › Supplementary Data/╩2╛▌/20 lncRNA╔·┤μ/sur.FGD5-AS1 p=0.016.pdf]

GUSBP11 high low

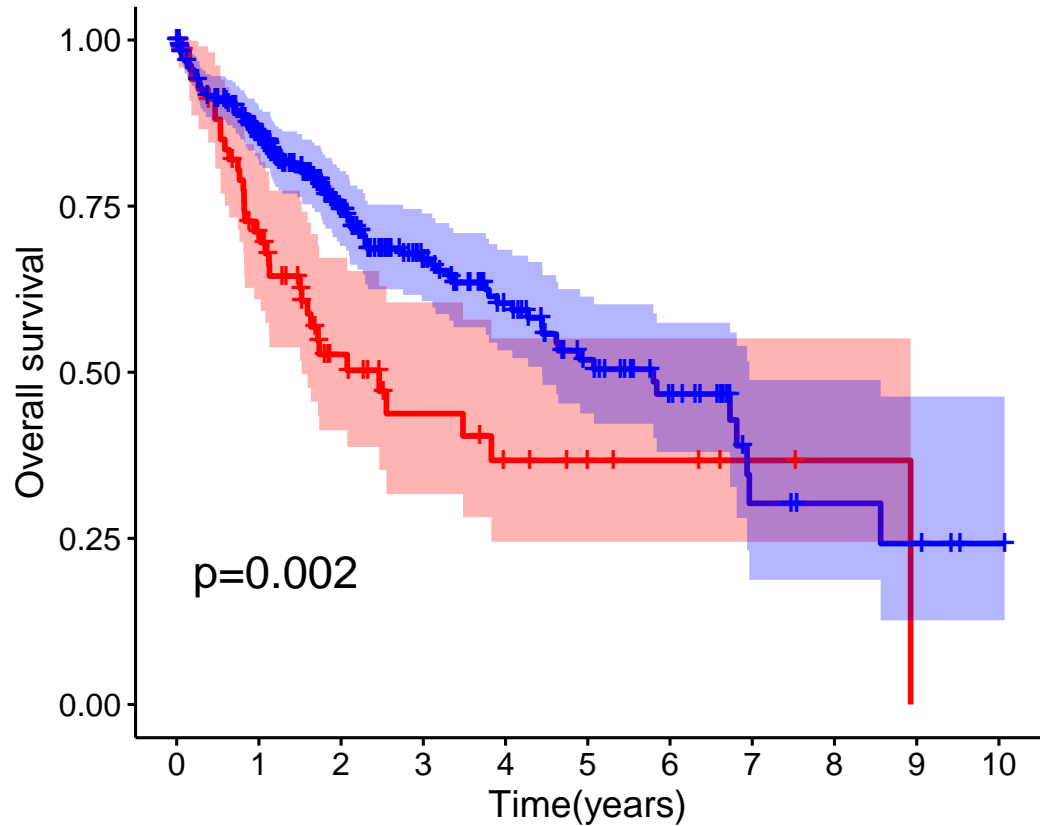

Supplement: Supplementary file 1 — Supplementary Information. [file 41598_2023_30695_MOESM1_ESM.zip › Supplementary Data/╩2╛▌/20 lncRNA╔·┤μ/sur.GUSBP11 p=0.002.pdf]

LINC00630 high low

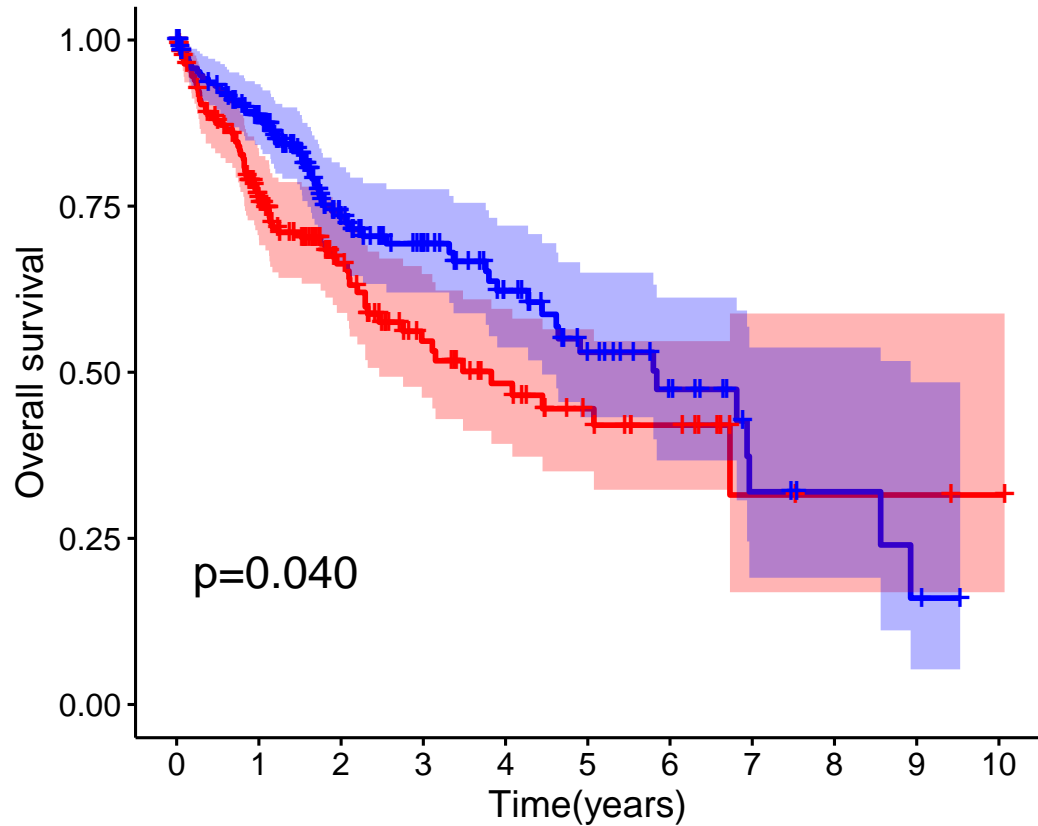

Supplement: Supplementary file 1 — Supplementary Information. [file 41598_2023_30695_MOESM1_ESM.zip › Supplementary Data/╩2╛▌/20 lncRNA╔·┤μ/sur.LINC00630 p=0.040.pdf]

LINC00997 high low

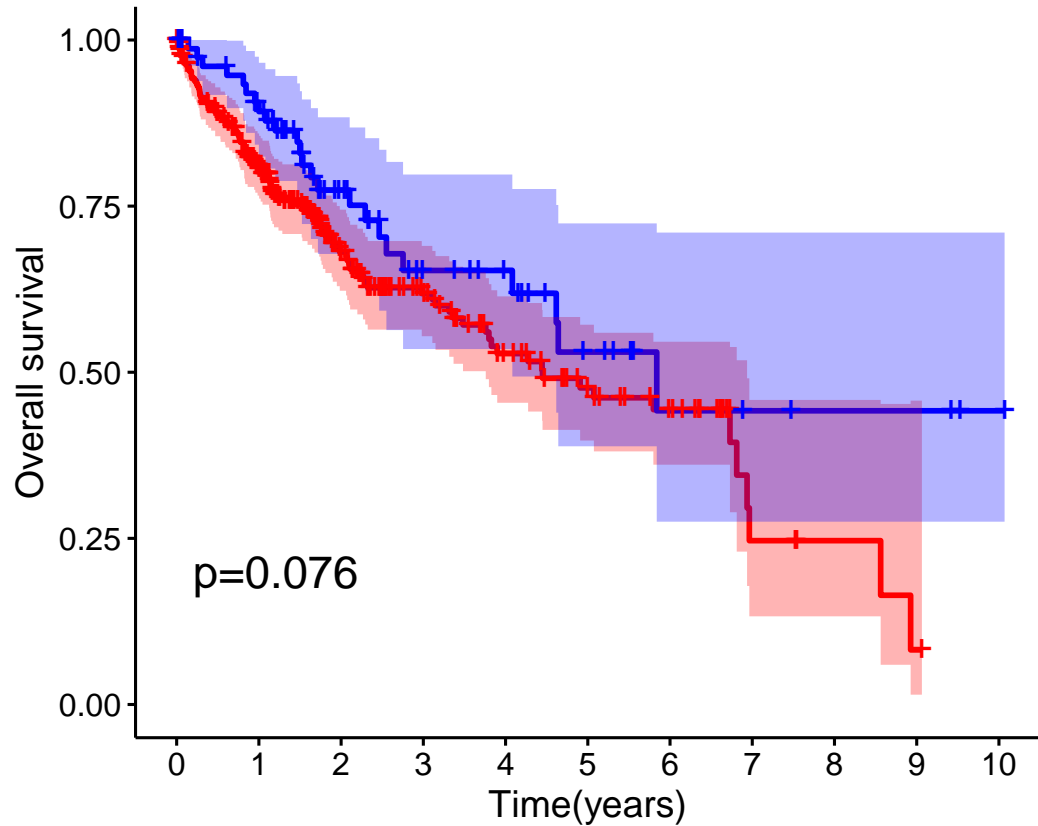

Supplement: Supplementary file 1 — Supplementary Information. [file 41598_2023_30695_MOESM1_ESM.zip › Supplementary Data/╩2╛▌/20 lncRNA╔·┤μ/sur.LINC00997 p=0.076.pdf]

SNHG16 + high + low

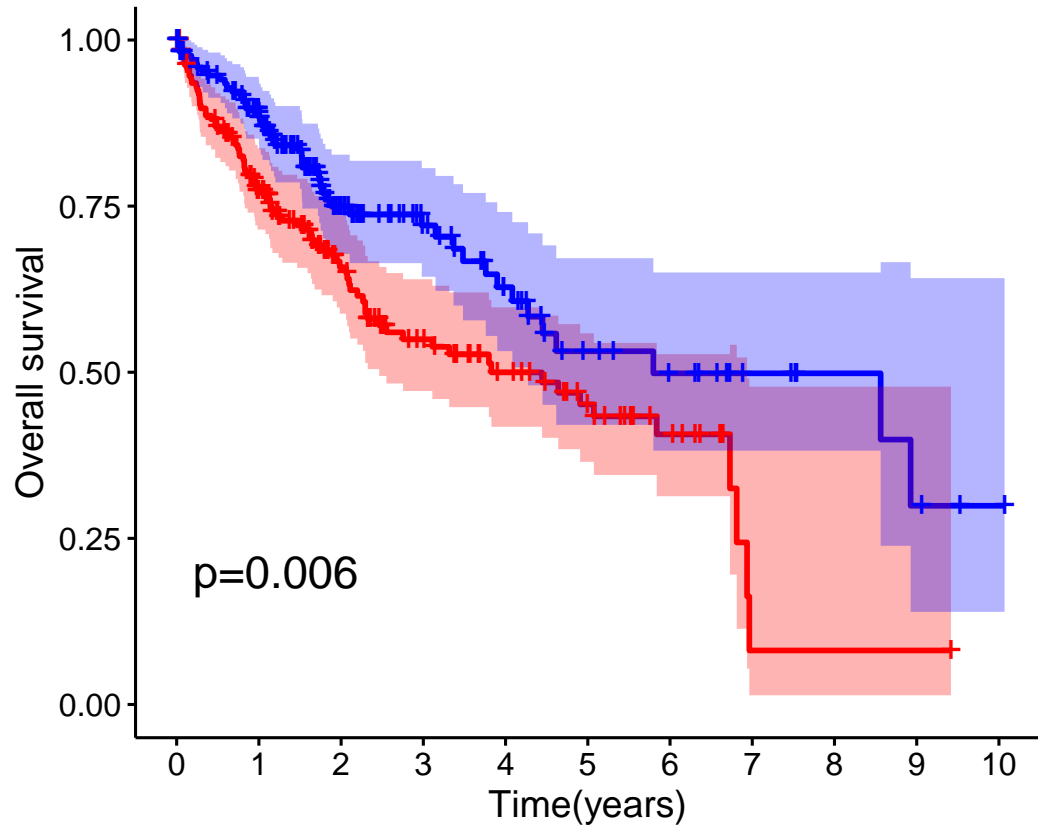

Supplement: Supplementary file 1 — Supplementary Information. [file 41598_2023_30695_MOESM1_ESM.zip › Supplementary Data/╩2╛▌/20 lncRNA╔·┤μ/sur.SNHG16 p=0.006.pdf]

TTC28-AS1 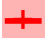 high 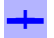 low

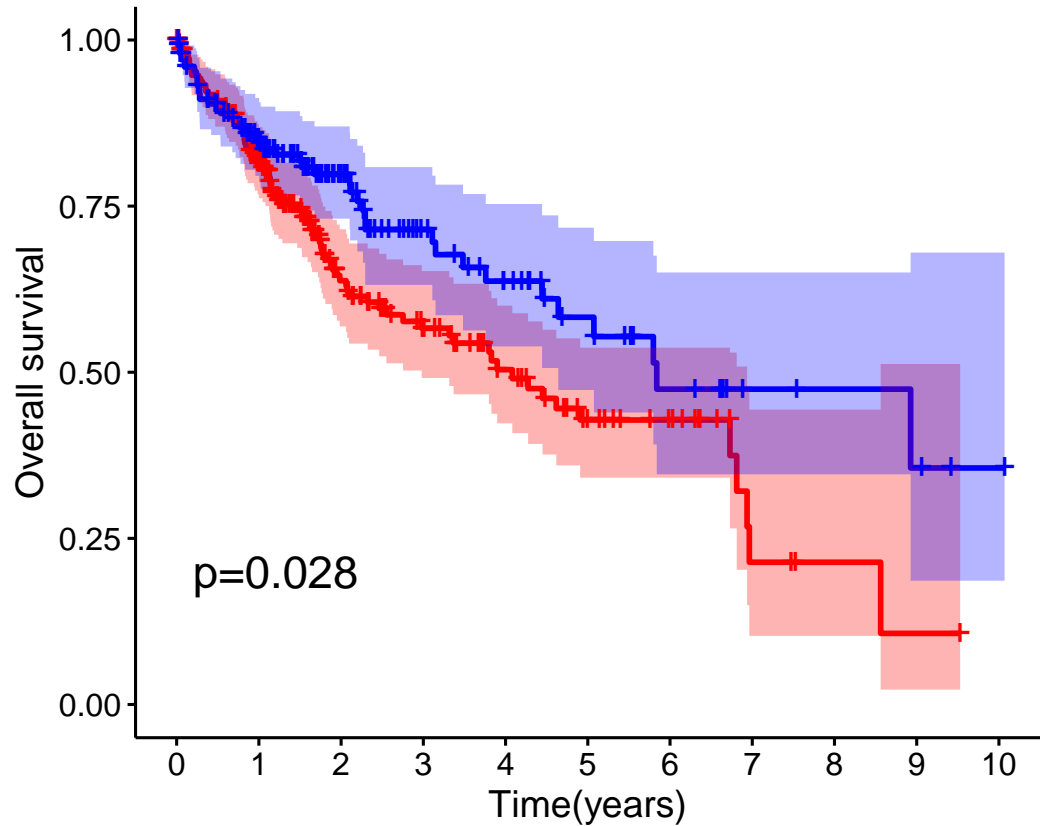

Supplement: Supplementary file 1 — Supplementary Information. [file 41598_2023_30695_MOESM1_ESM.zip › Supplementary Data/╩2╛▌/20 lncRNA╔·┤μ/sur.TTC28-AS1 p=0.028.pdf]

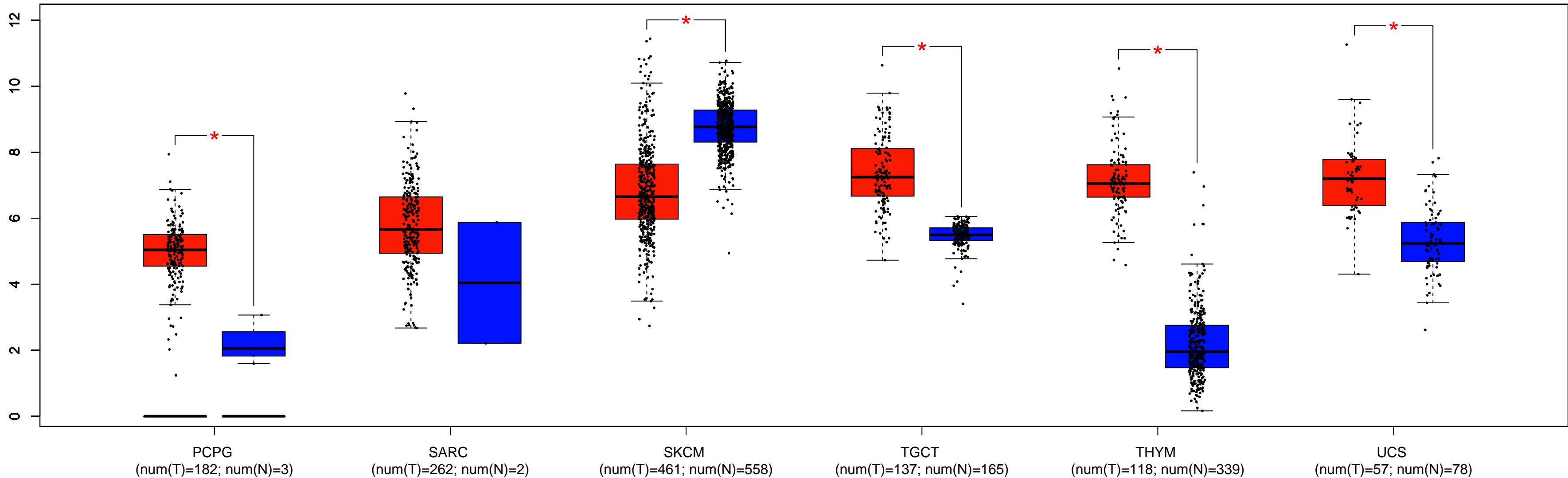

Supplement: Supplementary file 1 — Supplementary Information. [file 41598_2023_30695_MOESM1_ESM.zip › Supplementary Data/╩2╛▌/Expression analysis/FABP5_boxplot_7K4xA.pdf]

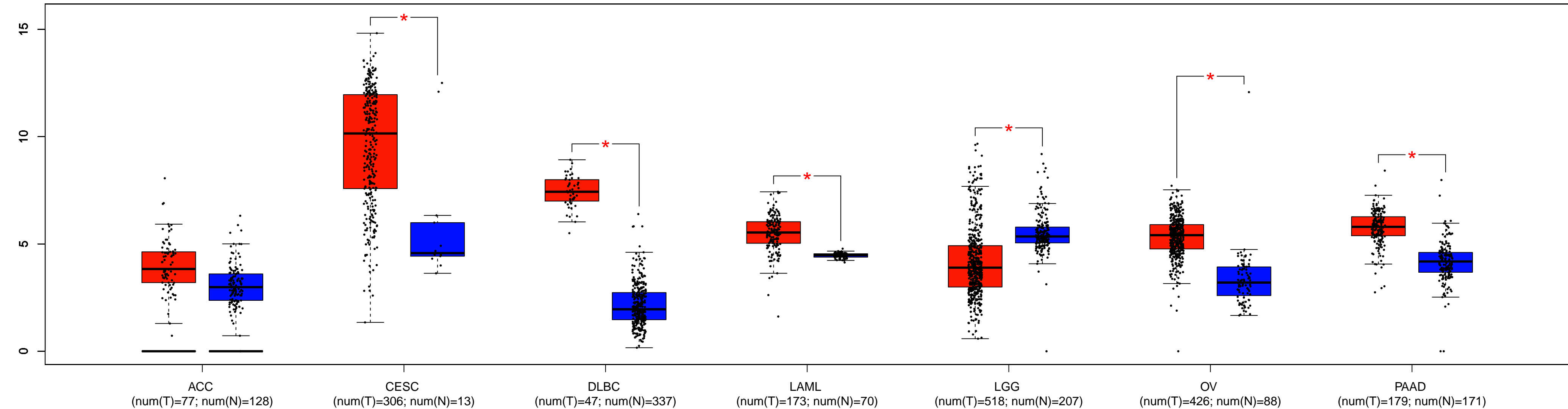

Supplement: Supplementary file 1 — Supplementary Information. [file 41598_2023_30695_MOESM1_ESM.zip › Supplementary Data/╩2╛▌/Expression analysis/FABP5_boxplot_bJEa3.pdf]

Type    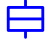 Normal    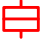 Tumor

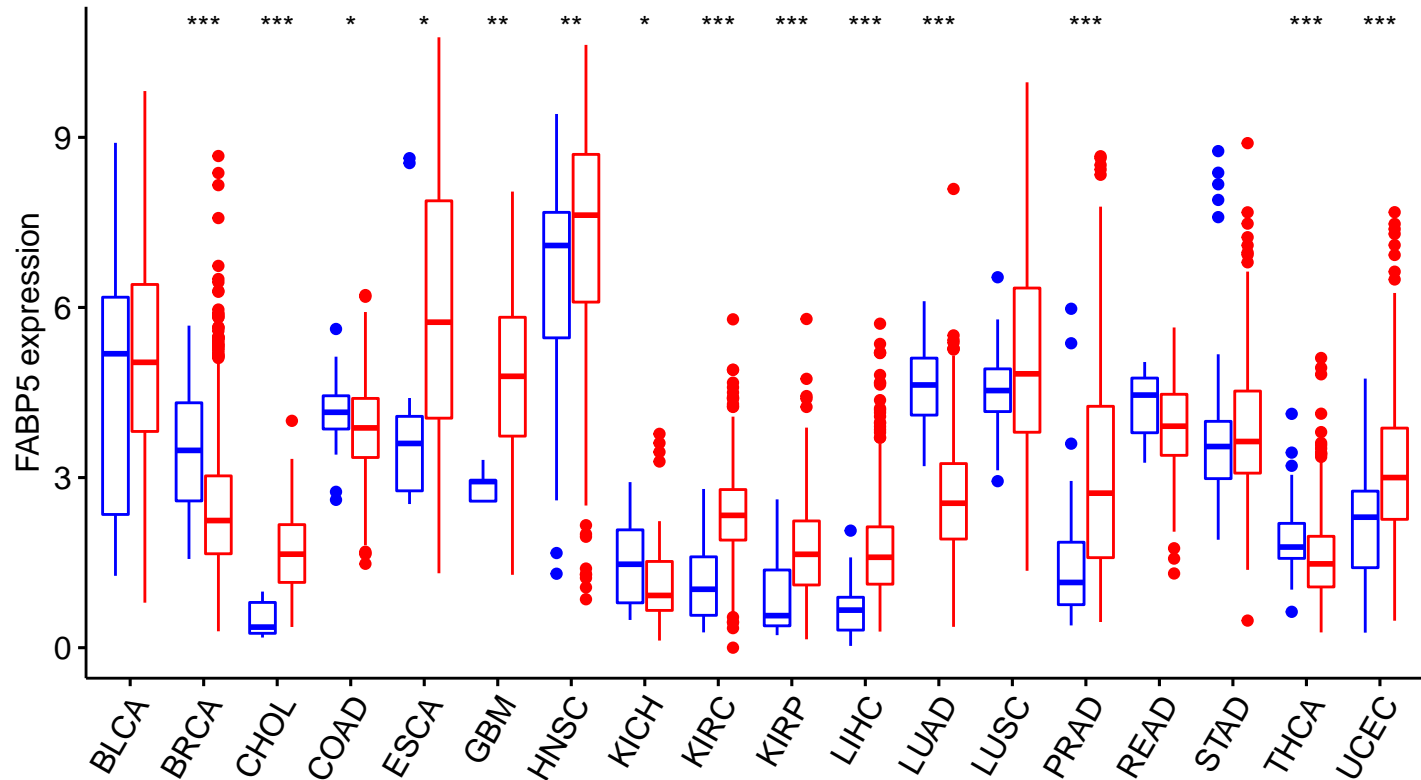

Supplement: Supplementary file 1 — Supplementary Information. [file 41598_2023_30695_MOESM1_ESM.zip › Supplementary Data/╩2╛▌/Expression analysis/boxplot.pdf]

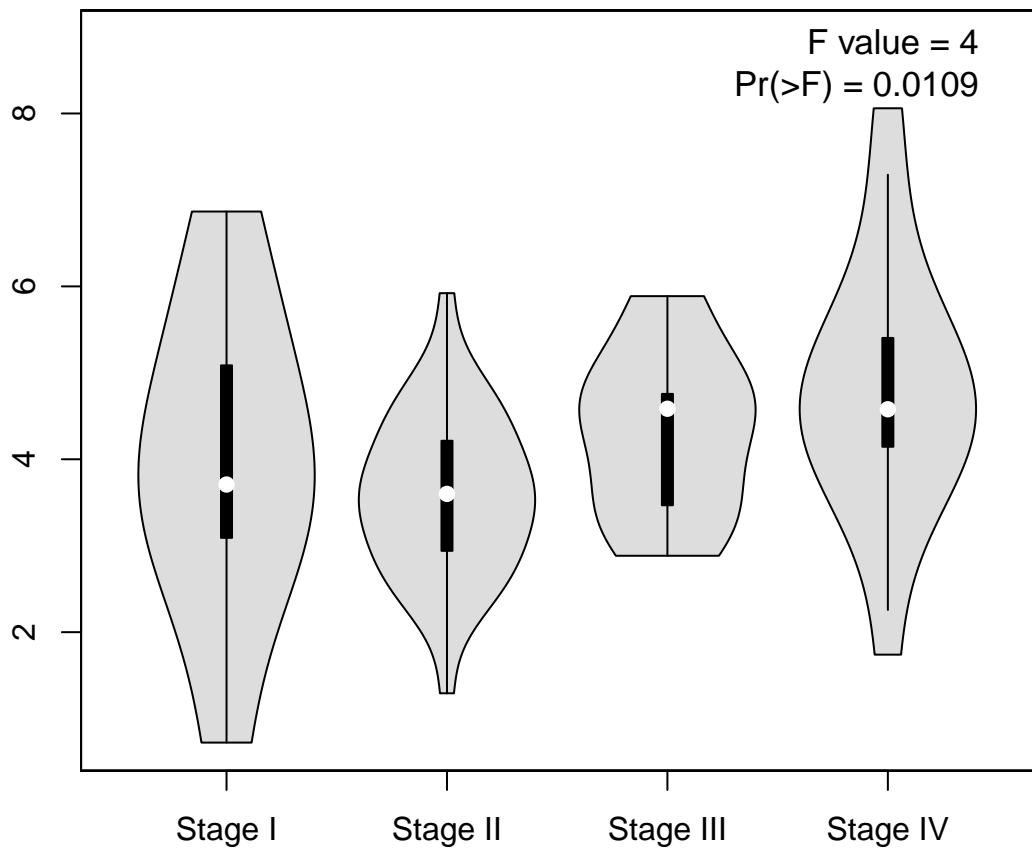

Supplement: Supplementary file 1 — Supplementary Information. [file 41598_2023_30695_MOESM1_ESM.zip › Supplementary Data/╩2╛▌/Expression analysis/stages/ACC0.011.pdf]

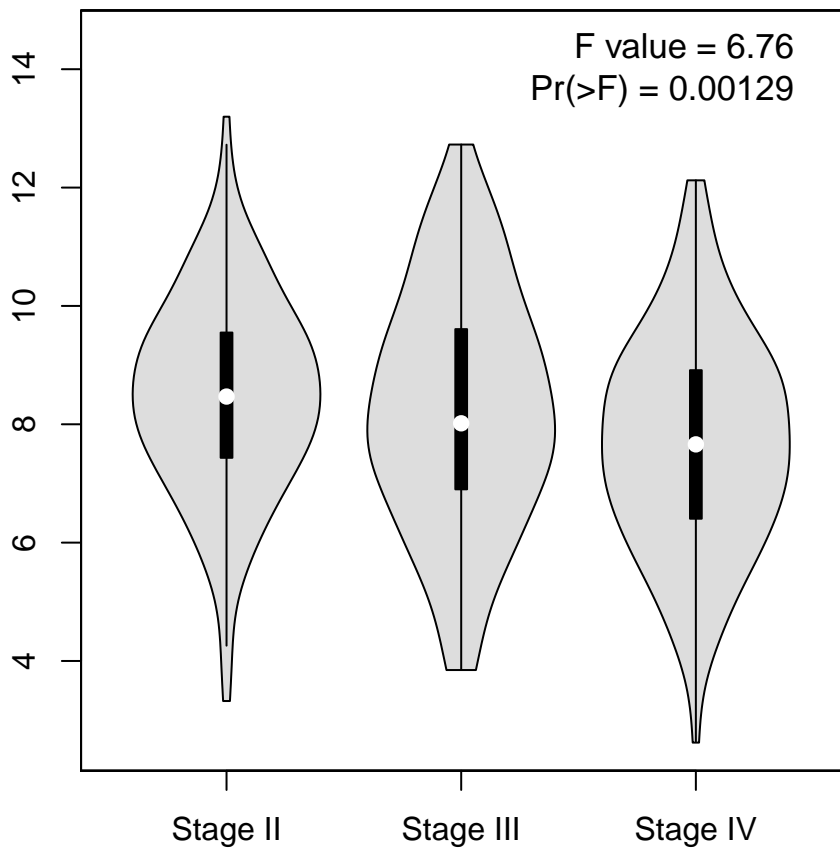

Supplement: Supplementary file 1 — Supplementary Information. [file 41598_2023_30695_MOESM1_ESM.zip › Supplementary Data/╩2╛▌/Expression analysis/stages/BLCA0.001.pdf]

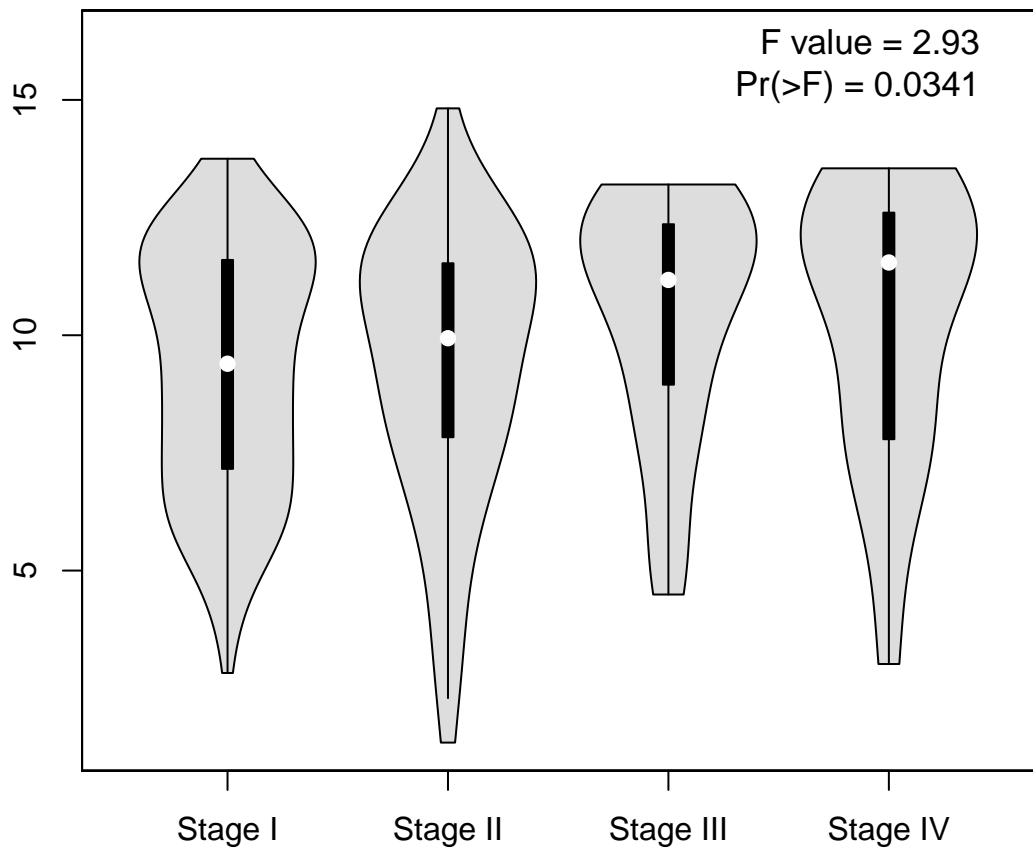

Supplement: Supplementary file 1 — Supplementary Information. [file 41598_2023_30695_MOESM1_ESM.zip › Supplementary Data/╩2╛▌/Expression analysis/stages/CESC0.034.pdf]

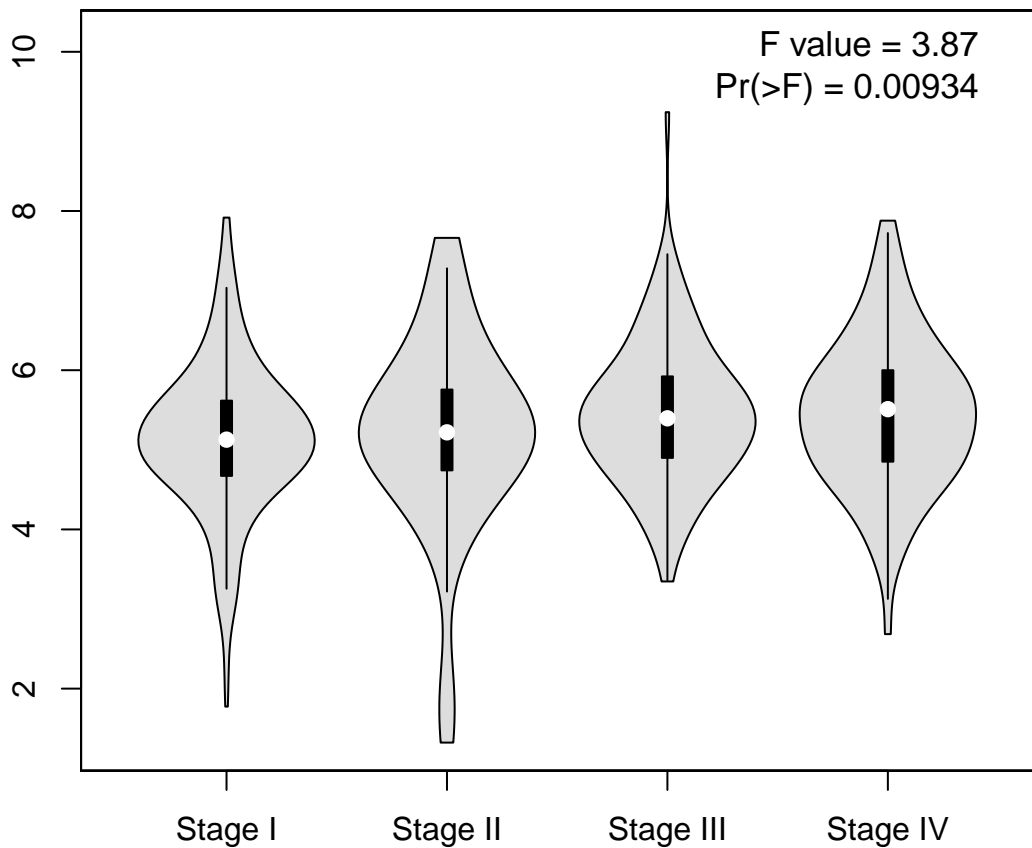

Supplement: Supplementary file 1 — Supplementary Information. [file 41598_2023_30695_MOESM1_ESM.zip › Supplementary Data/╩2╛▌/Expression analysis/stages/KIRC0.009.pdf]

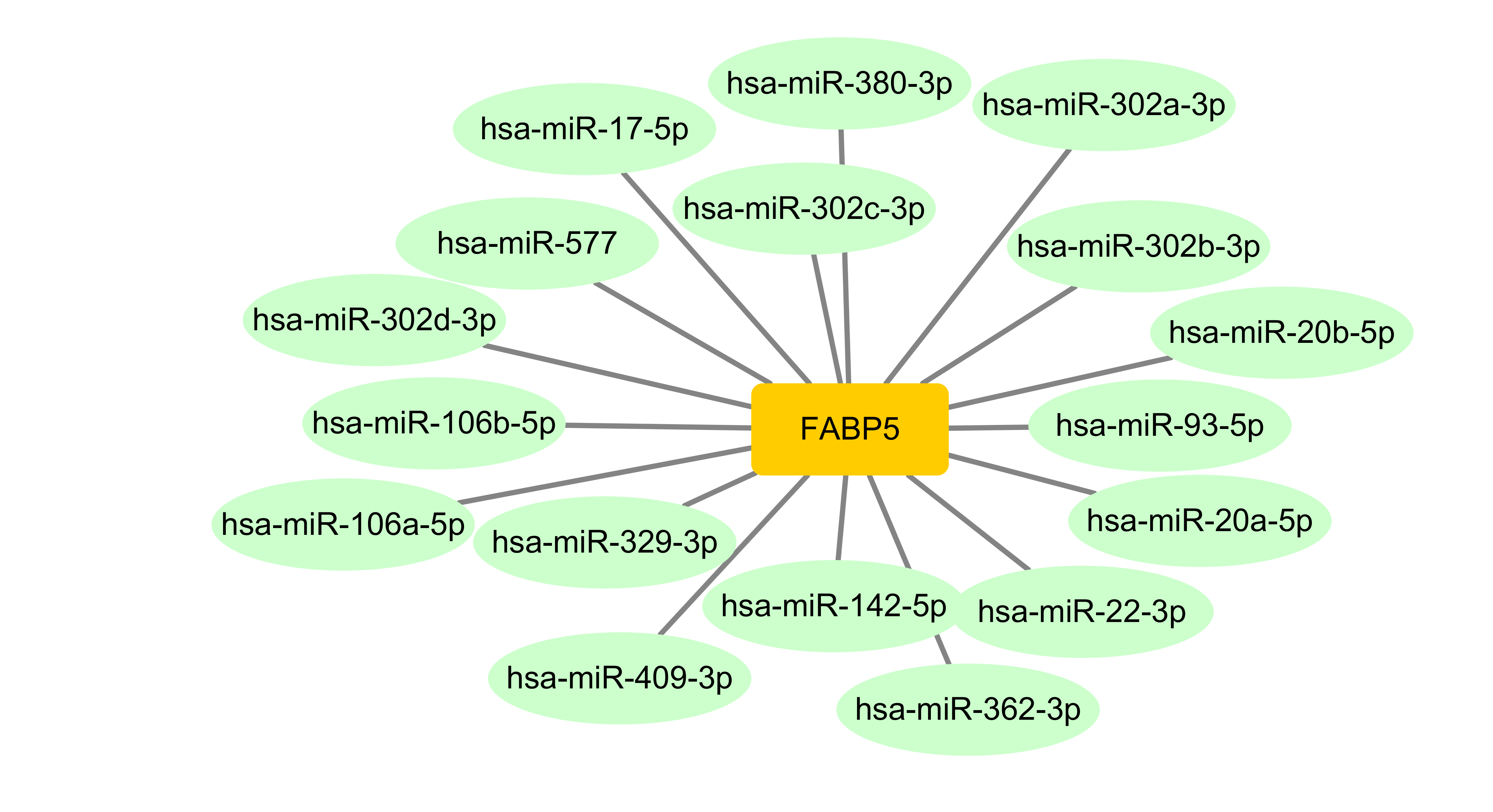

Supplement: Supplementary file 1 — Supplementary Information. [file 41598_2023_30695_MOESM1_ESM.zip › Supplementary Data/╩2╛▌/FABP5╜ß║╧╡─miRNA/miRNA.txt.png]

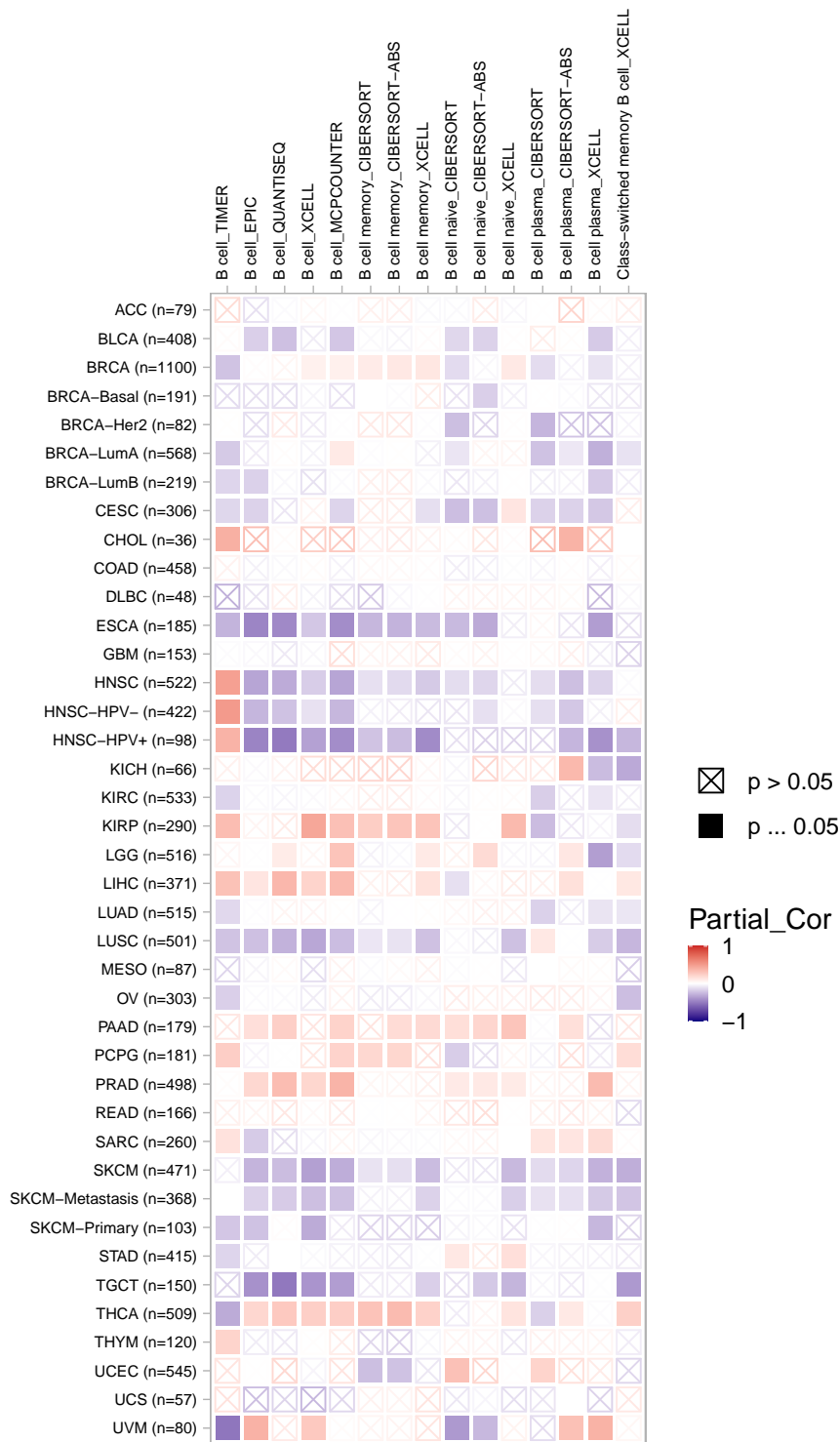

Supplement: Supplementary file 1 — Supplementary Information. [file 41598_2023_30695_MOESM1_ESM.zip › Supplementary Data/╩2╛▌/Immune/B cell.pdf]

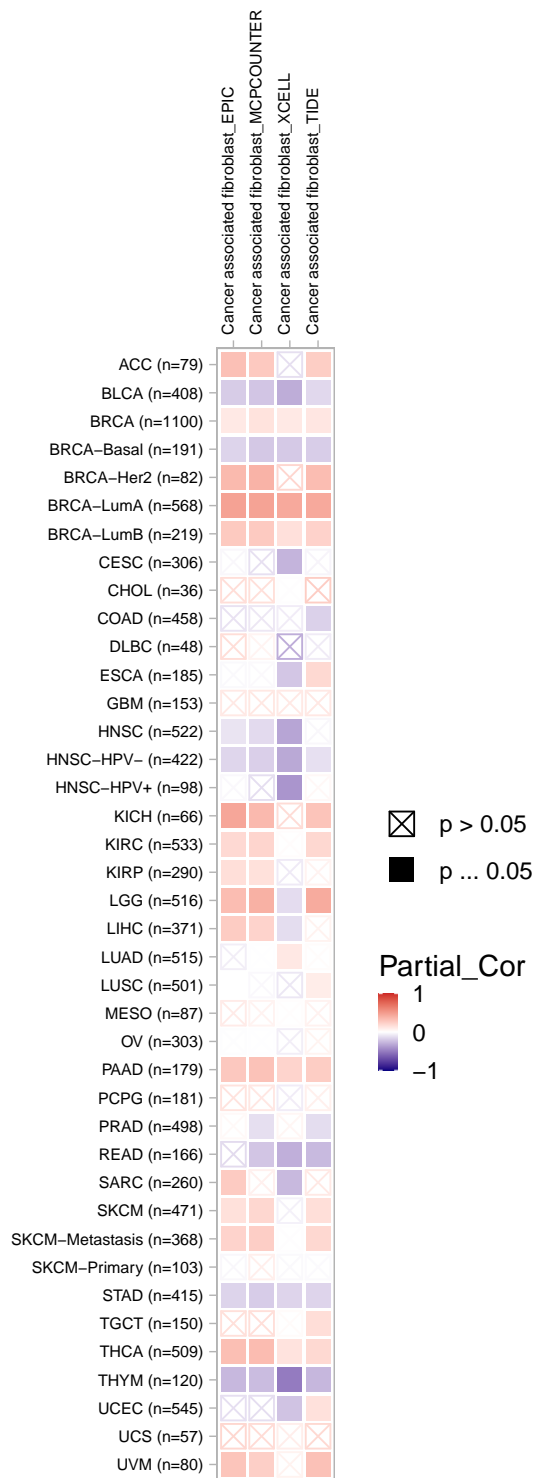

Supplement: Supplementary file 1 — Supplementary Information. [file 41598_2023_30695_MOESM1_ESM.zip › Supplementary Data/╩2╛▌/Immune/CAFs.pdf]

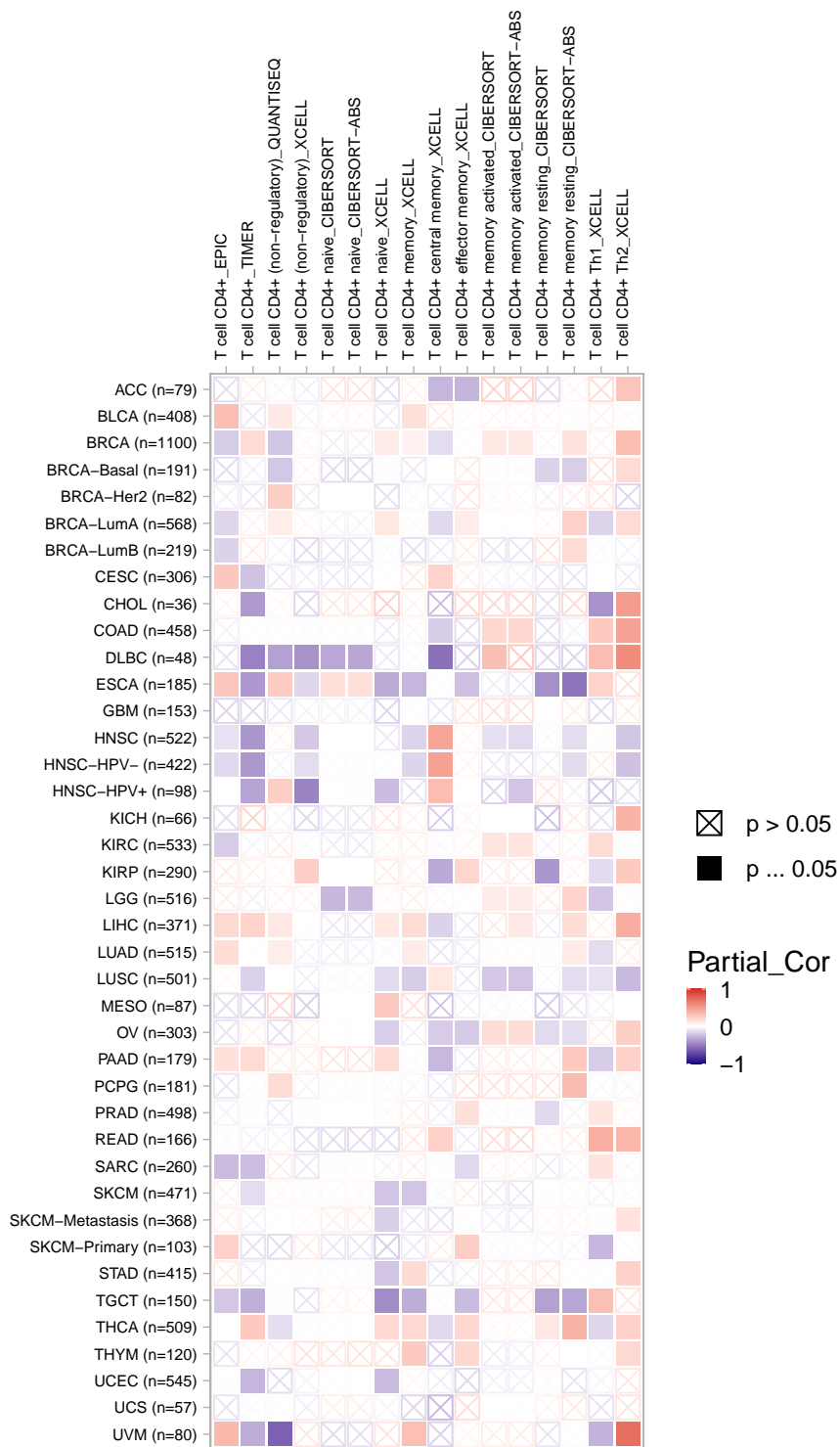

Supplement: Supplementary file 1 — Supplementary Information. [file 41598_2023_30695_MOESM1_ESM.zip › Supplementary Data/╩2╛▌/Immune/CD4+ T cell.pdf]

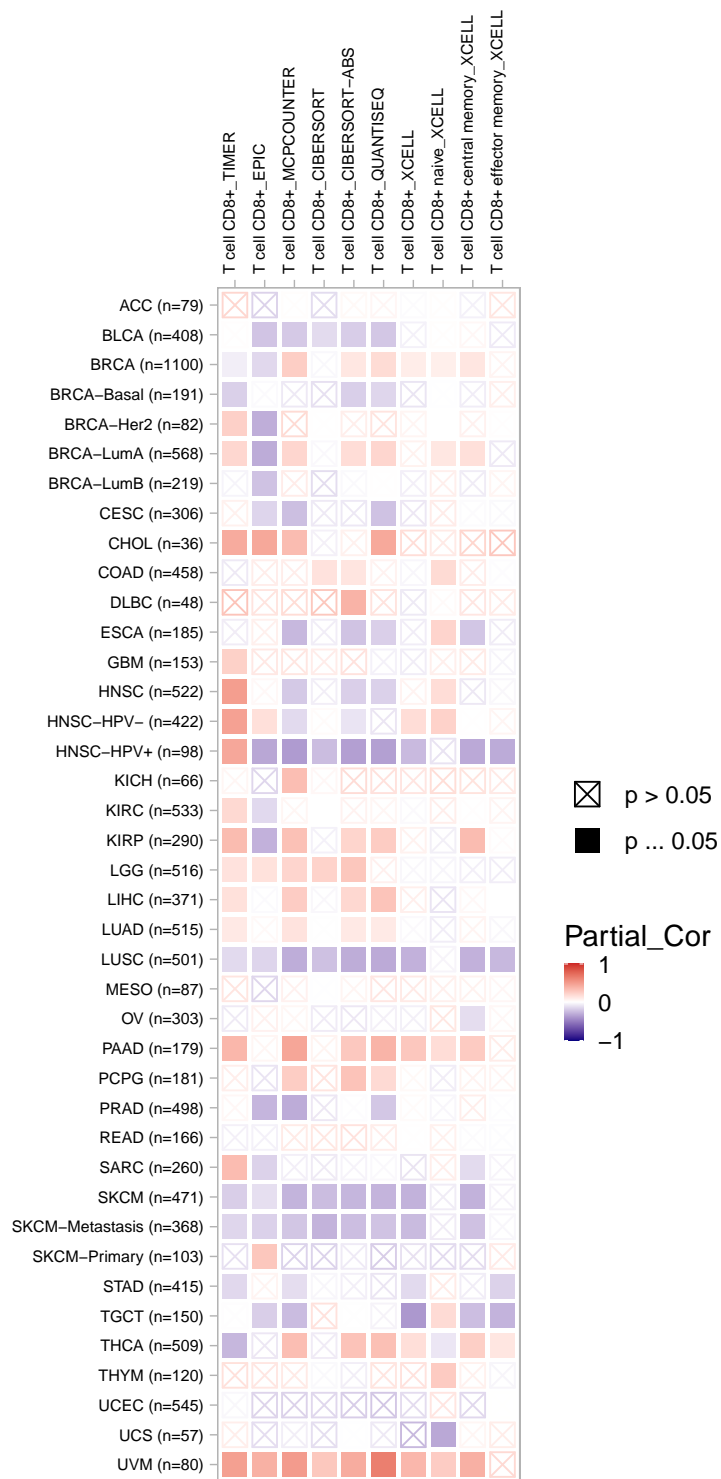

Supplement: Supplementary file 1 — Supplementary Information. [file 41598_2023_30695_MOESM1_ESM.zip › Supplementary Data/╩2╛▌/Immune/CD8+ T cell.pdf]

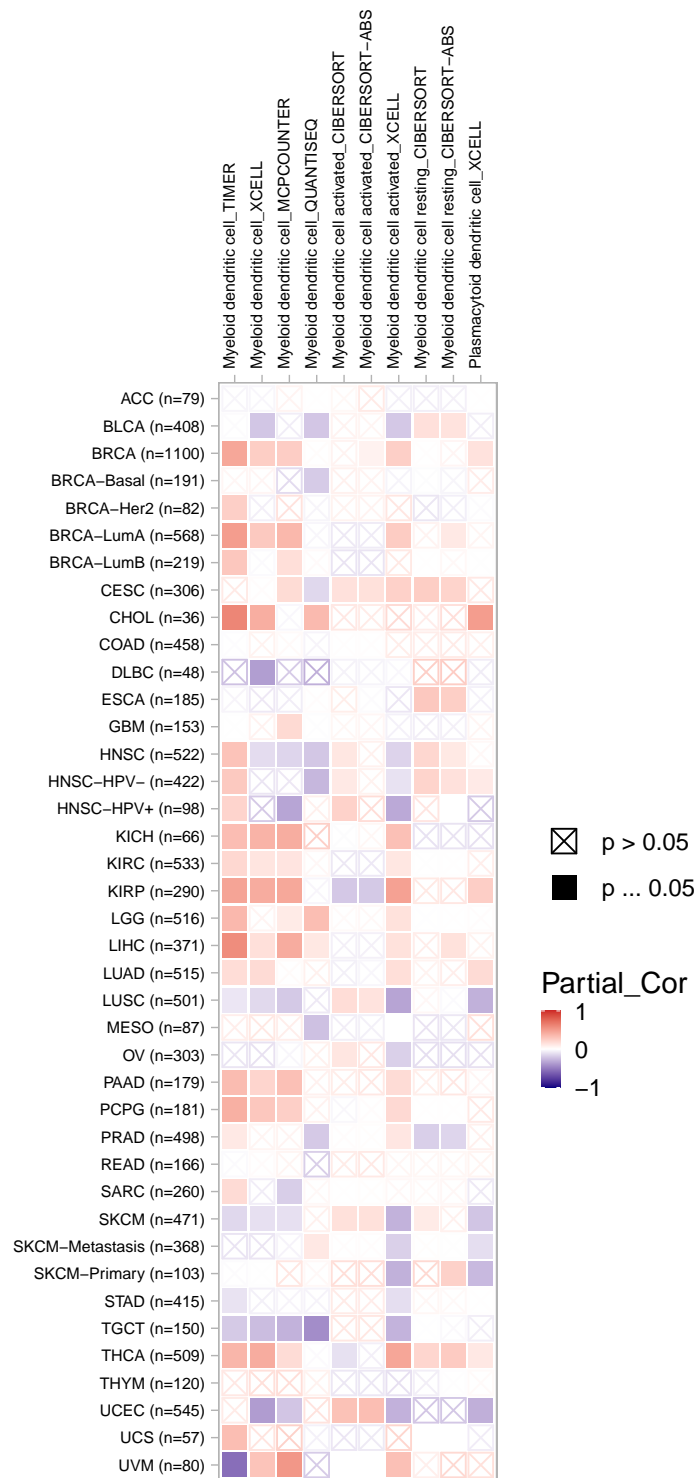

Supplement: Supplementary file 1 — Supplementary Information. [file 41598_2023_30695_MOESM1_ESM.zip › Supplementary Data/╩2╛▌/Immune/DC.pdf]

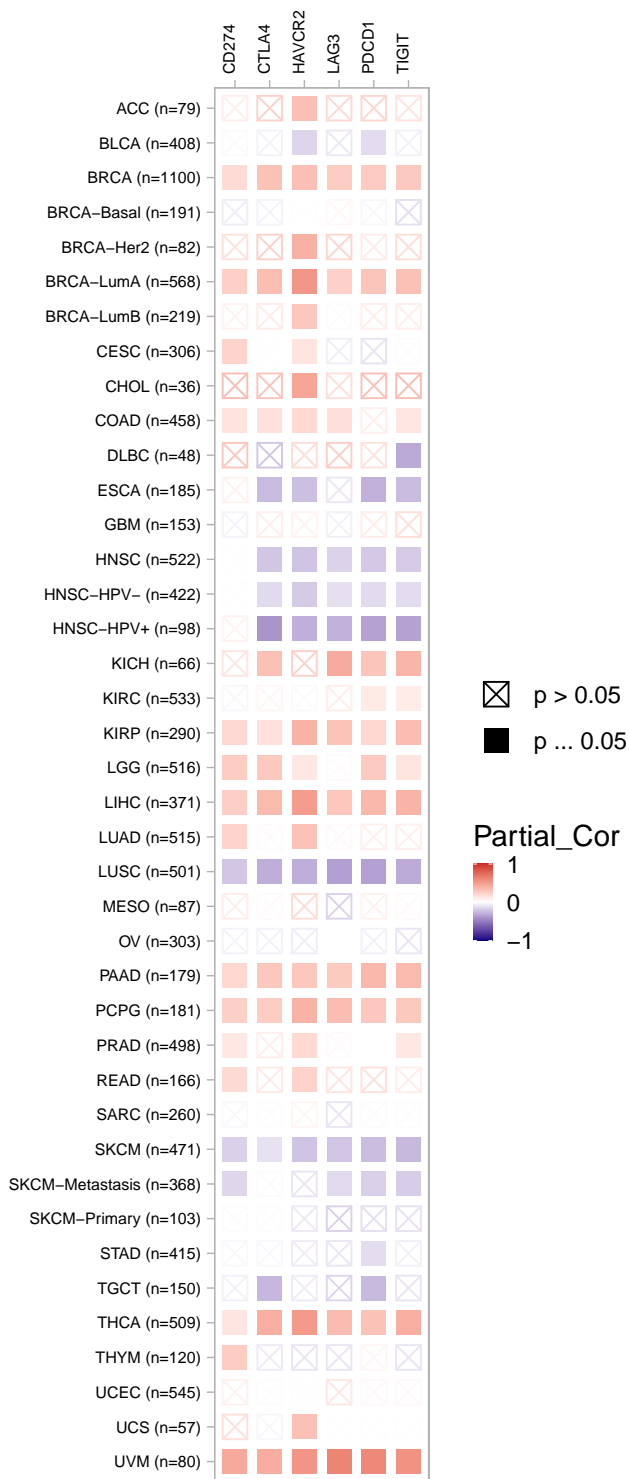

Supplement: Supplementary file 1 — Supplementary Information. [file 41598_2023_30695_MOESM1_ESM.zip › Supplementary Data/╩2╛▌/Immune/Immune checkpoints.pdf]

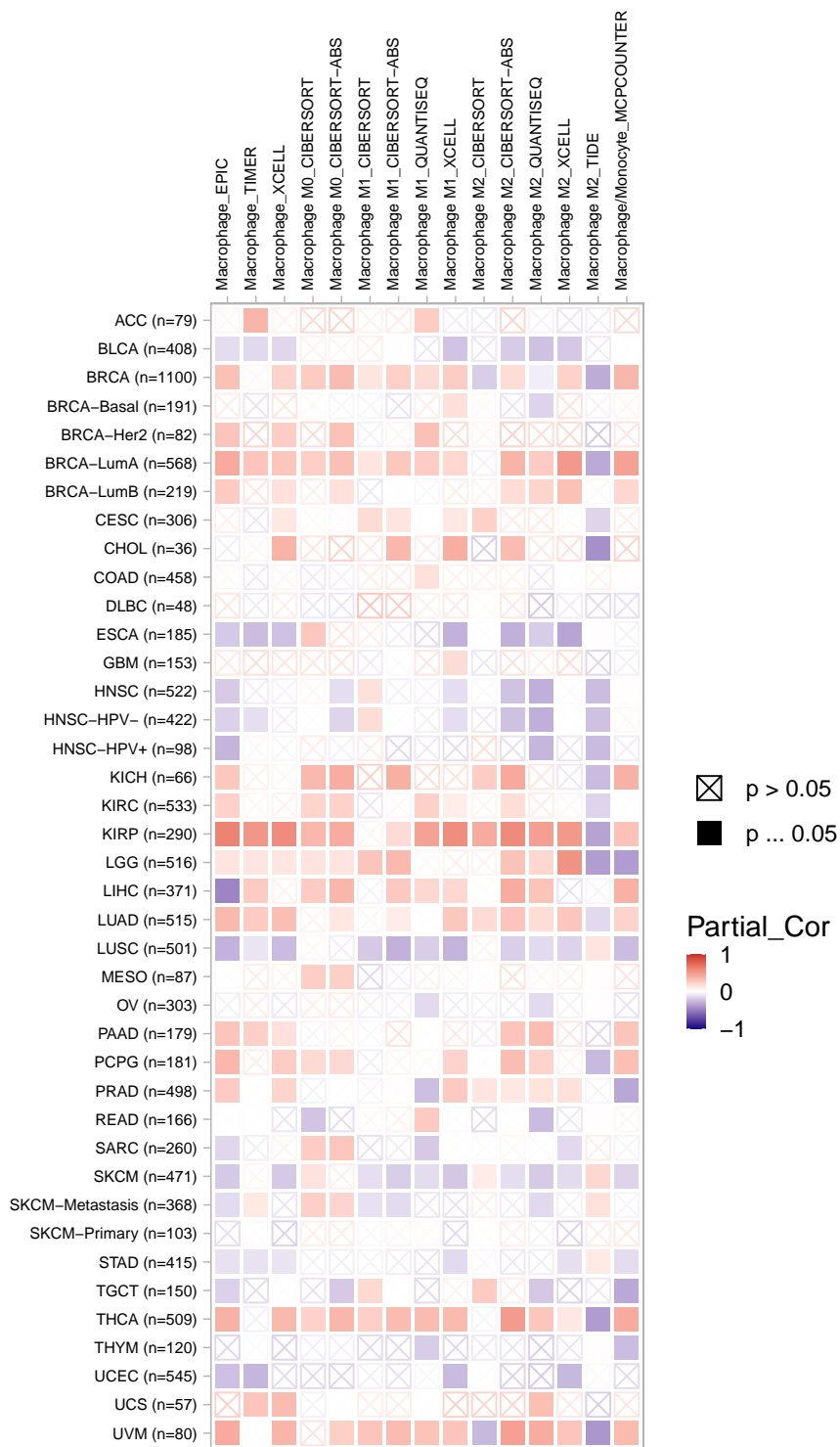

Supplement: Supplementary file 1 — Supplementary Information. [file 41598_2023_30695_MOESM1_ESM.zip › Supplementary Data/╩2╛▌/Immune/Macrophage.pdf]

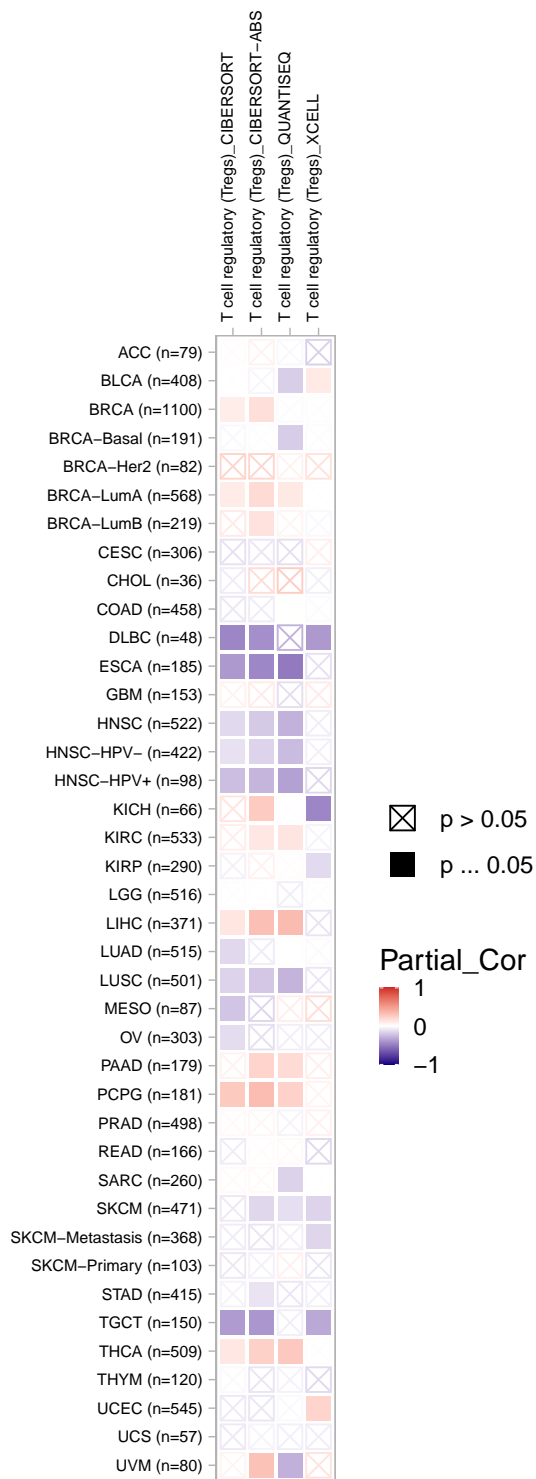

Supplement: Supplementary file 1 — Supplementary Information. [file 41598_2023_30695_MOESM1_ESM.zip › Supplementary Data/╩2╛▌/Immune/Treg.pdf]

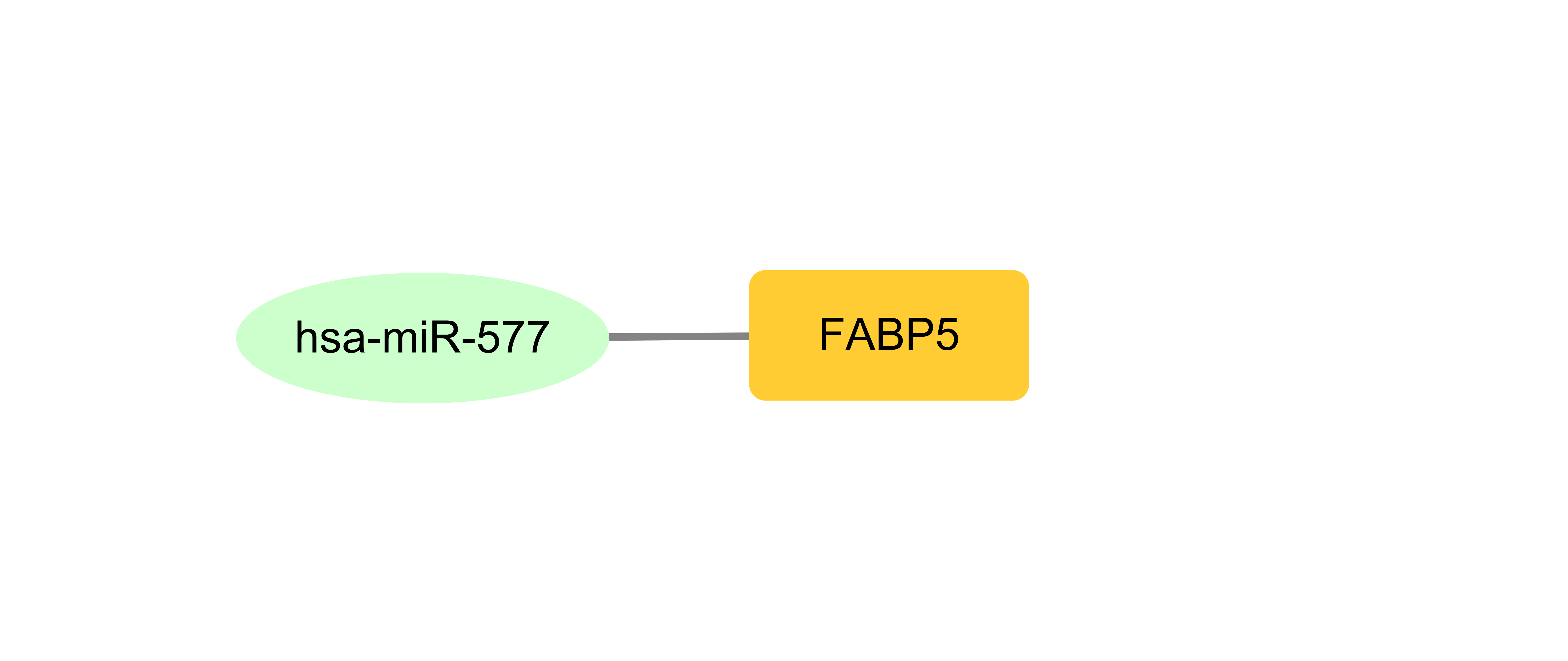

Supplement: Supplementary file 1 — Supplementary Information. [file 41598_2023_30695_MOESM1_ESM.zip › Supplementary Data/╩2╛▌/Network in KIRC/ceRNA.network.txt.png]

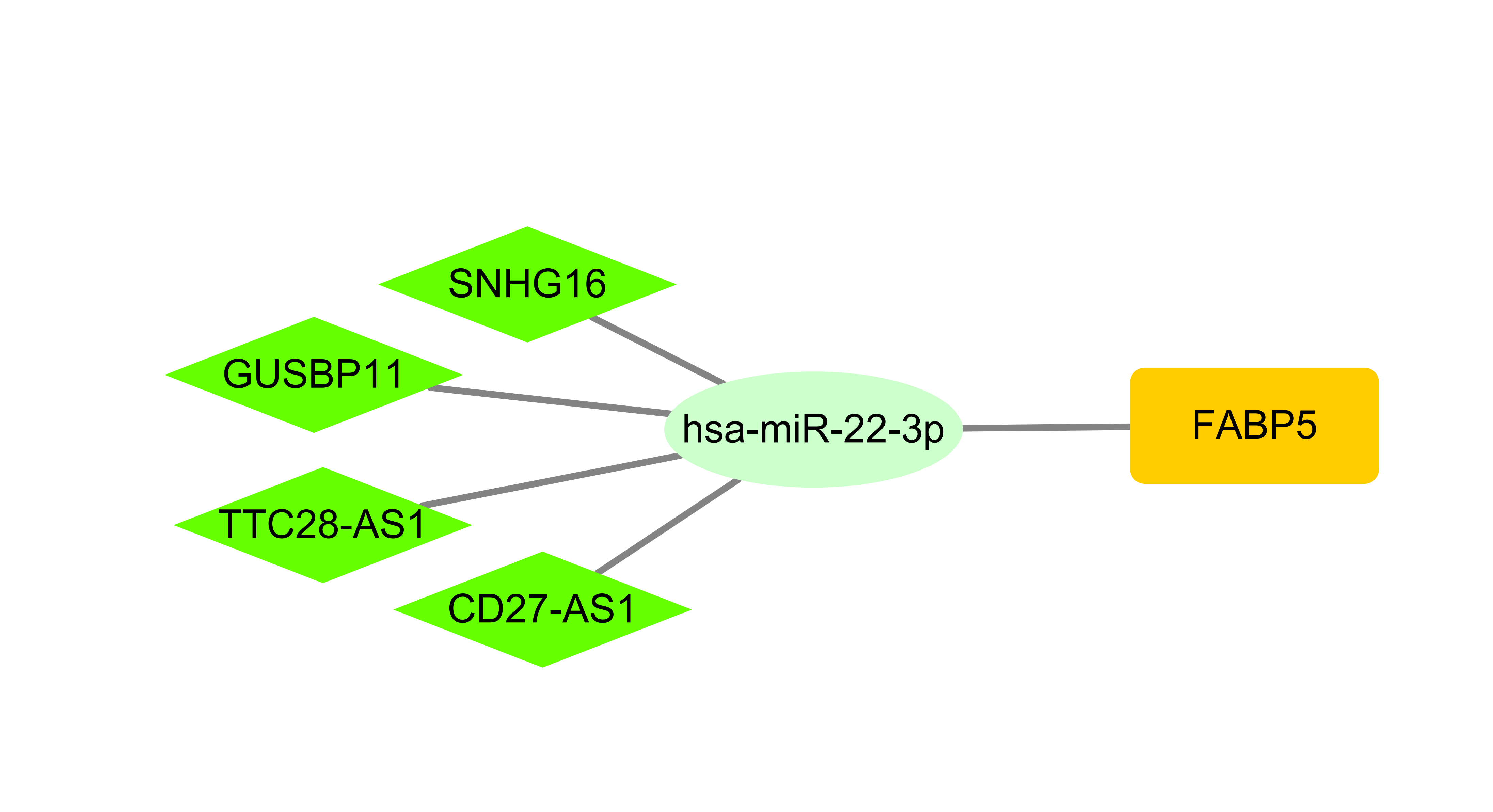

Supplement: Supplementary file 1 — Supplementary Information. [file 41598_2023_30695_MOESM1_ESM.zip › Supplementary Data/╩2╛▌/Network in LIHC/ceRNA.network.txt.png]

# Disease Free Survival

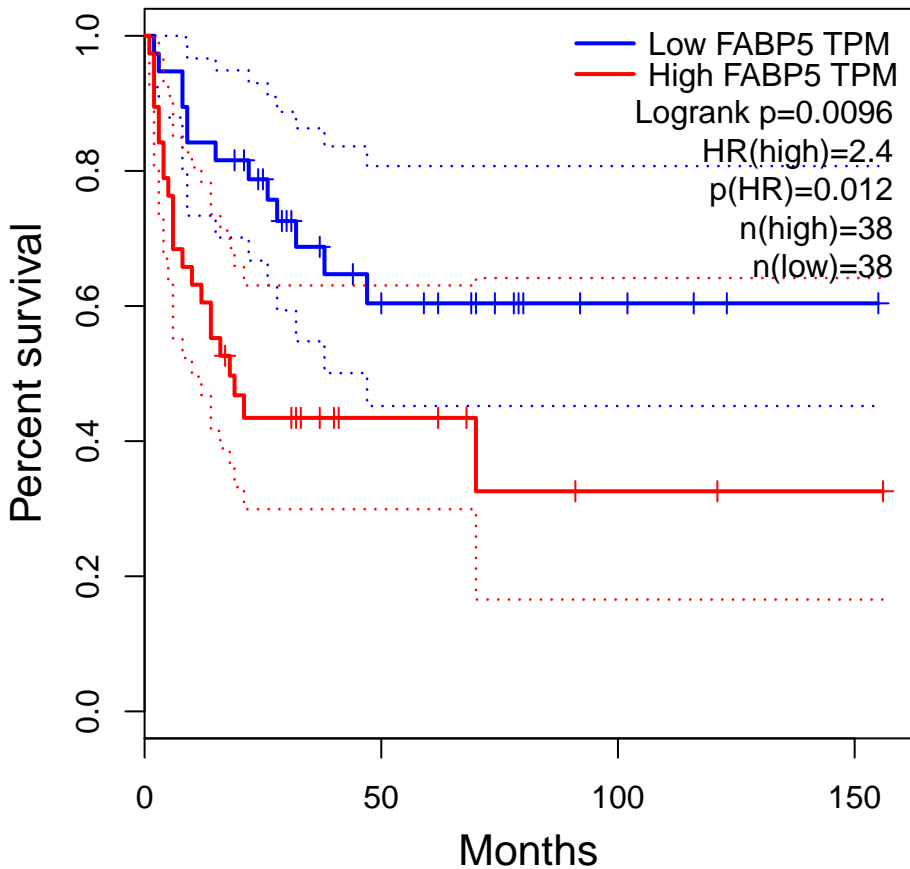

Supplement: Supplementary file 1 — Supplementary Information. [file 41598_2023_30695_MOESM1_ESM.zip › Supplementary Data/╩2╛▌/Survival analysis/DFS/ACC0.012.pdf]

# Disease Free Survival

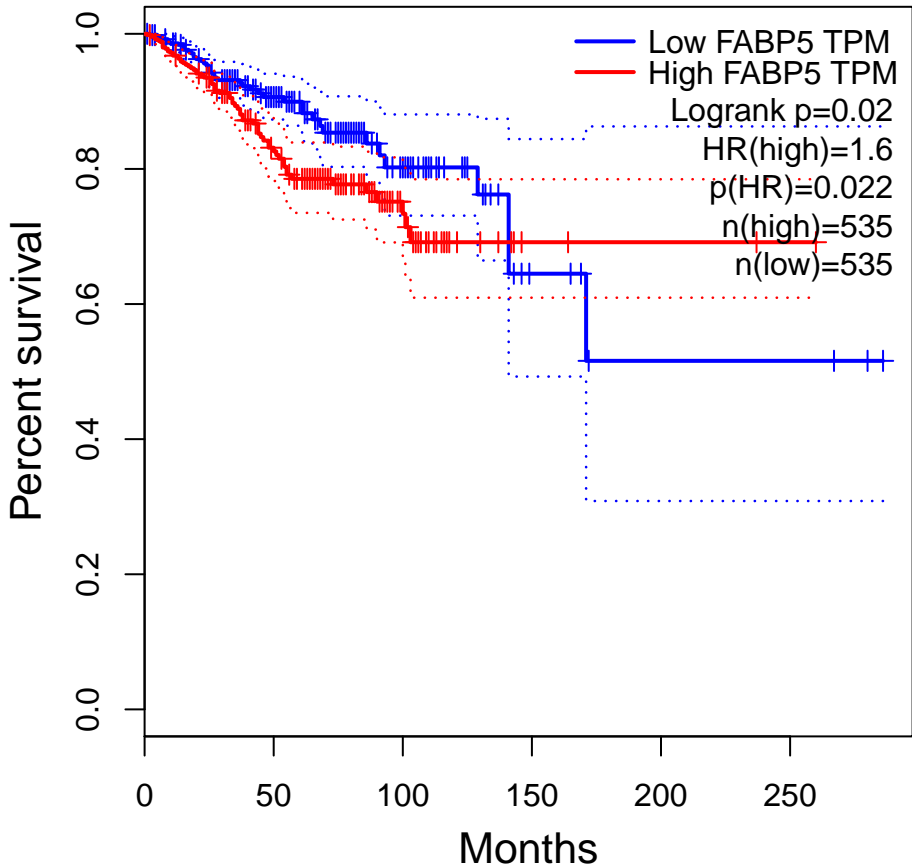

Supplement: Supplementary file 1 — Supplementary Information. [file 41598_2023_30695_MOESM1_ESM.zip › Supplementary Data/╩2╛▌/Survival analysis/DFS/BRCA0.022.pdf]

# Disease Free Survival

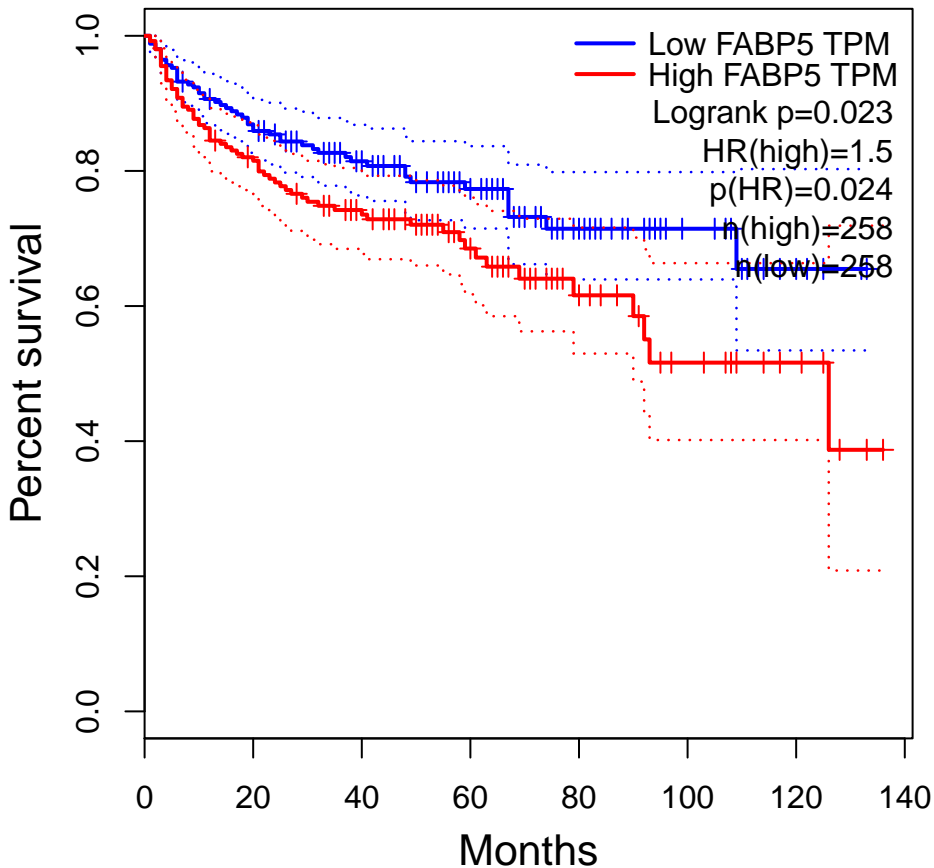

Supplement: Supplementary file 1 — Supplementary Information. [file 41598_2023_30695_MOESM1_ESM.zip › Supplementary Data/╩2╛▌/Survival analysis/DFS/KIRC0.024.pdf]

# Disease Free Survival

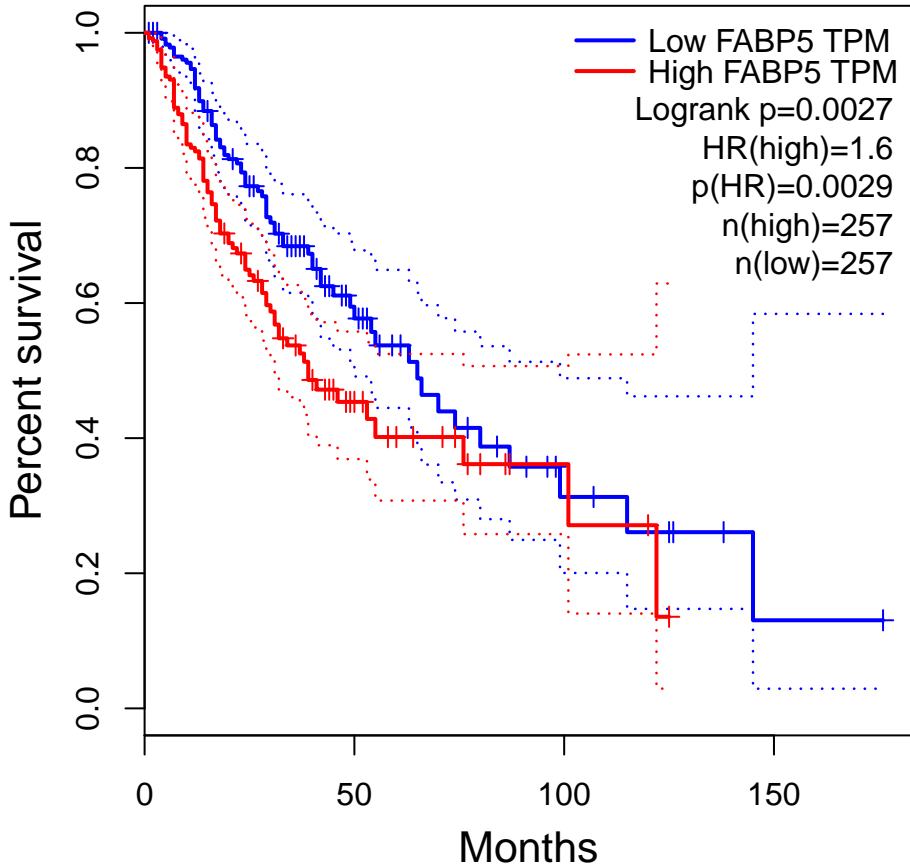

Supplement: Supplementary file 1 — Supplementary Information. [file 41598_2023_30695_MOESM1_ESM.zip › Supplementary Data/╩2╛▌/Survival analysis/DFS/LGG0.003.pdf]

# Disease Free Survival

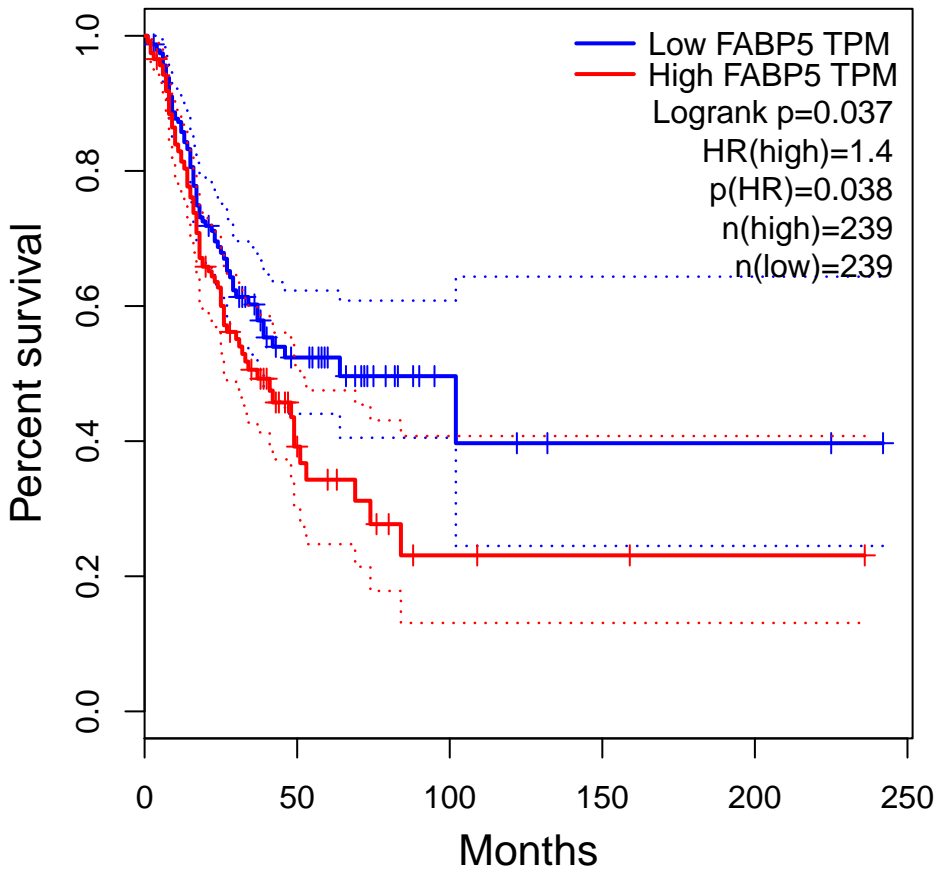

Supplement: Supplementary file 1 — Supplementary Information. [file 41598_2023_30695_MOESM1_ESM.zip › Supplementary Data/╩2╛▌/Survival analysis/DFS/LUAD0.038.pdf]

# Disease Free Survival

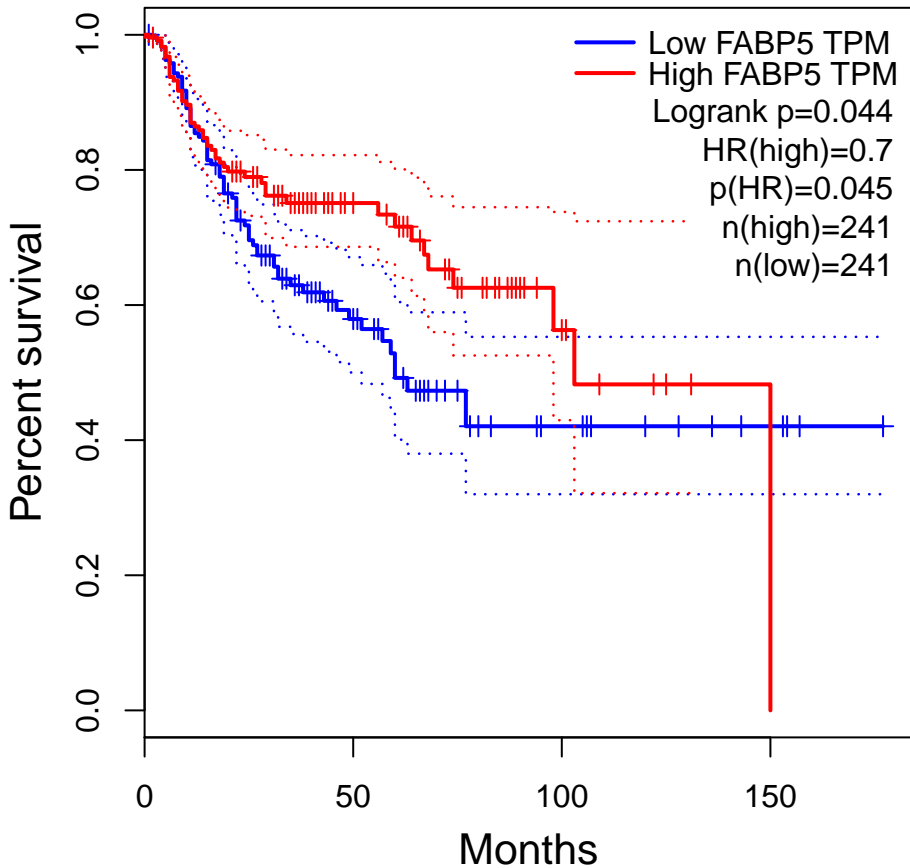

Supplement: Supplementary file 1 — Supplementary Information. [file 41598_2023_30695_MOESM1_ESM.zip › Supplementary Data/╩2╛▌/Survival analysis/DFS/LUSC0.045.pdf]

# Disease Free Survival

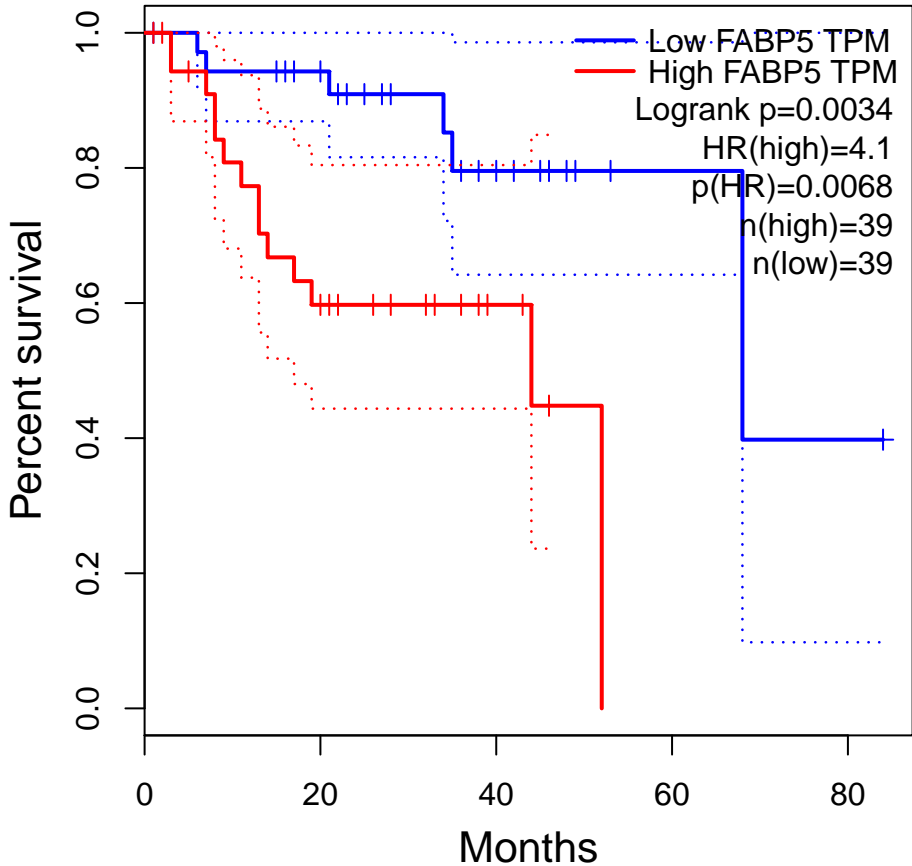

Supplement: Supplementary file 1 — Supplementary Information. [file 41598_2023_30695_MOESM1_ESM.zip › Supplementary Data/╩2╛▌/Survival analysis/DFS/UVM0.007.pdf]

# Overall Survival

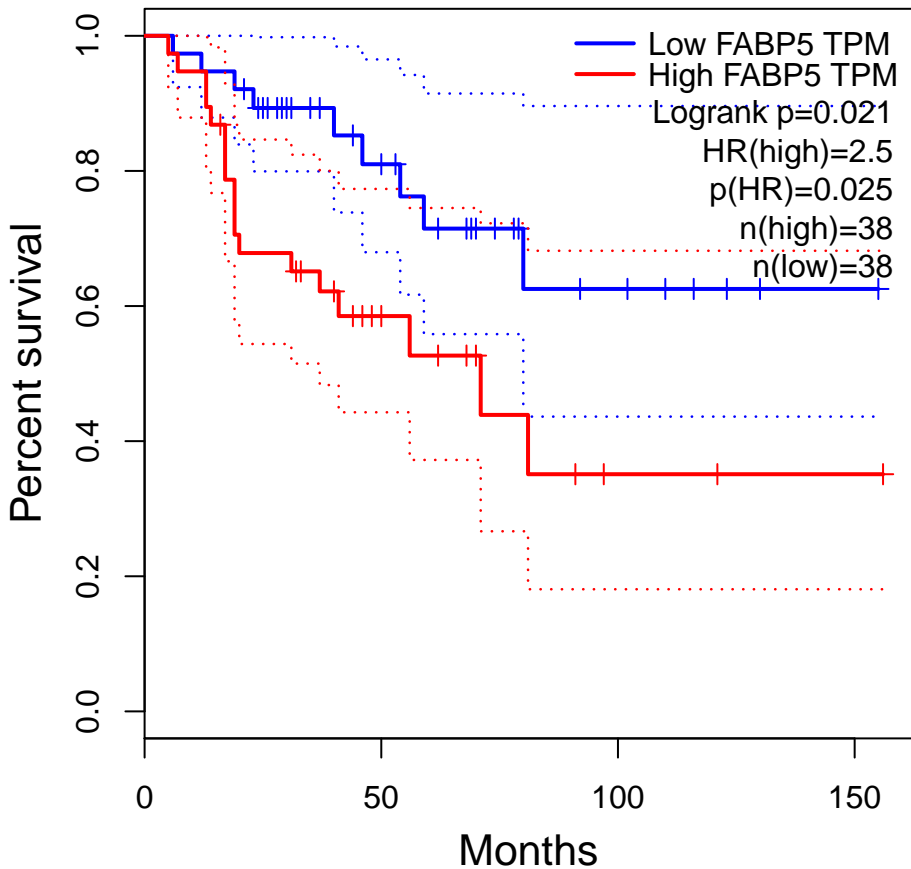

Supplement: Supplementary file 1 — Supplementary Information. [file 41598_2023_30695_MOESM1_ESM.zip › Supplementary Data/╩2╛▌/Survival analysis/OS/ACC0.025.pdf]

# Overall Survival

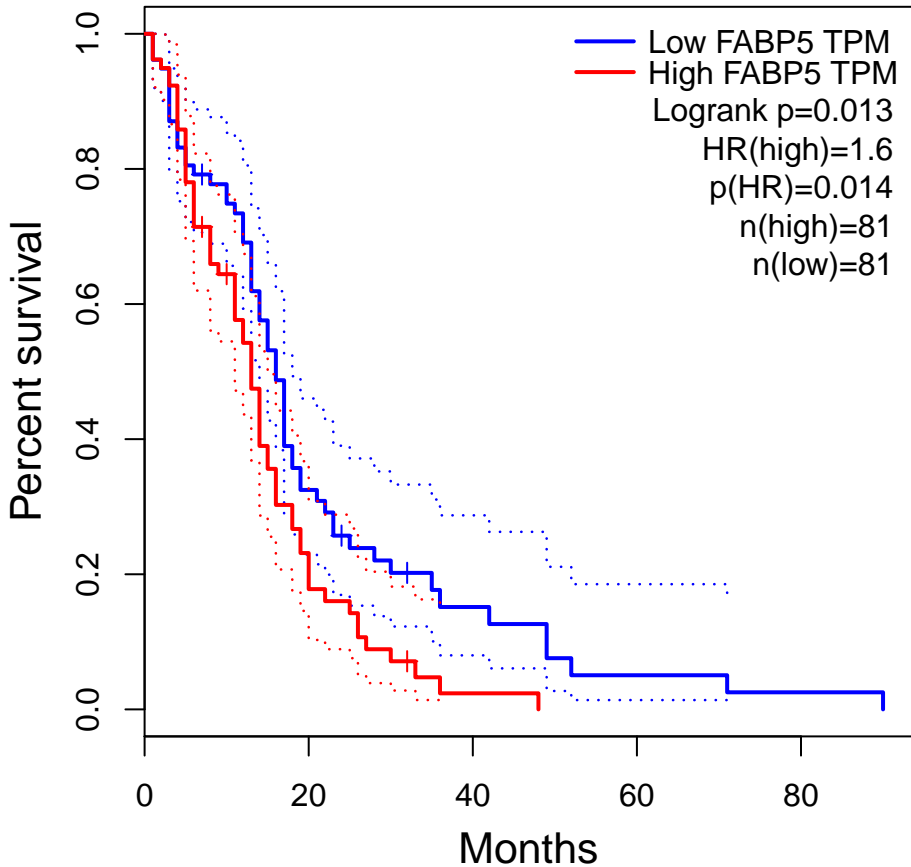

Supplement: Supplementary file 1 — Supplementary Information. [file 41598_2023_30695_MOESM1_ESM.zip › Supplementary Data/╩2╛▌/Survival analysis/OS/GBM0.014.pdf]

# Overall Survival

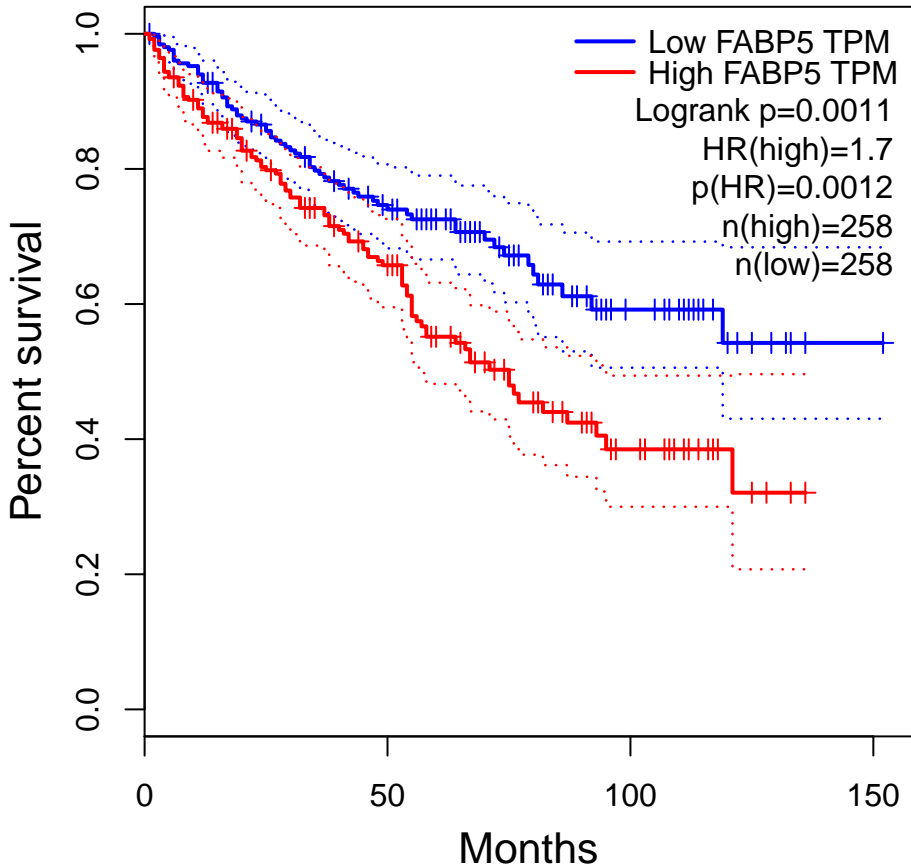

Supplement: Supplementary file 1 — Supplementary Information. [file 41598_2023_30695_MOESM1_ESM.zip › Supplementary Data/╩2╛▌/Survival analysis/OS/KIRC0.001.pdf]

# Overall Survival

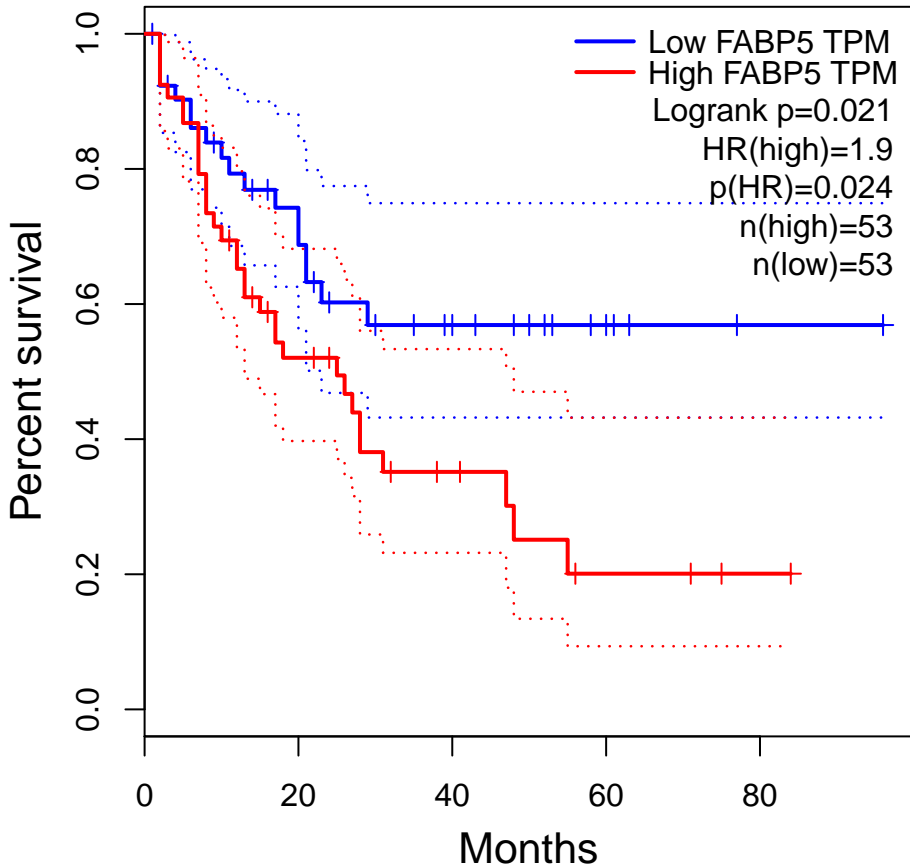

Supplement: Supplementary file 1 — Supplementary Information. [file 41598_2023_30695_MOESM1_ESM.zip › Supplementary Data/╩2╛▌/Survival analysis/OS/LAML0.024.pdf]

# Overall Survival

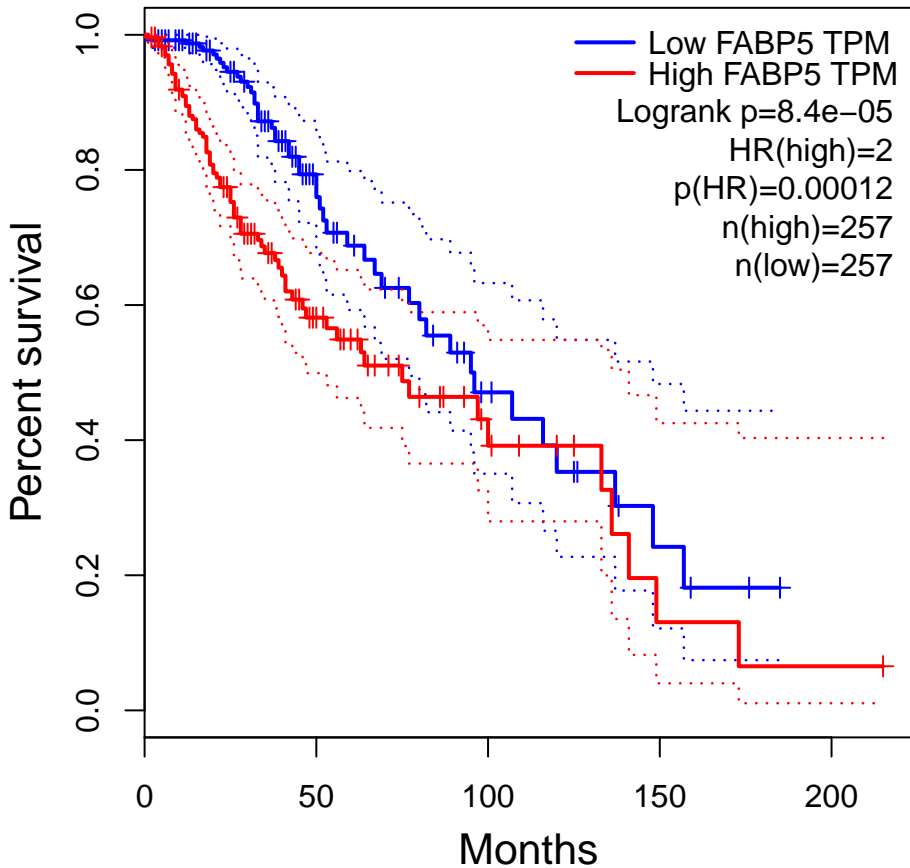

Supplement: Supplementary file 1 — Supplementary Information. [file 41598_2023_30695_MOESM1_ESM.zip › Supplementary Data/╩2╛▌/Survival analysis/OS/LGG╨í╙┌0.001.pdf]

# Overall Survival

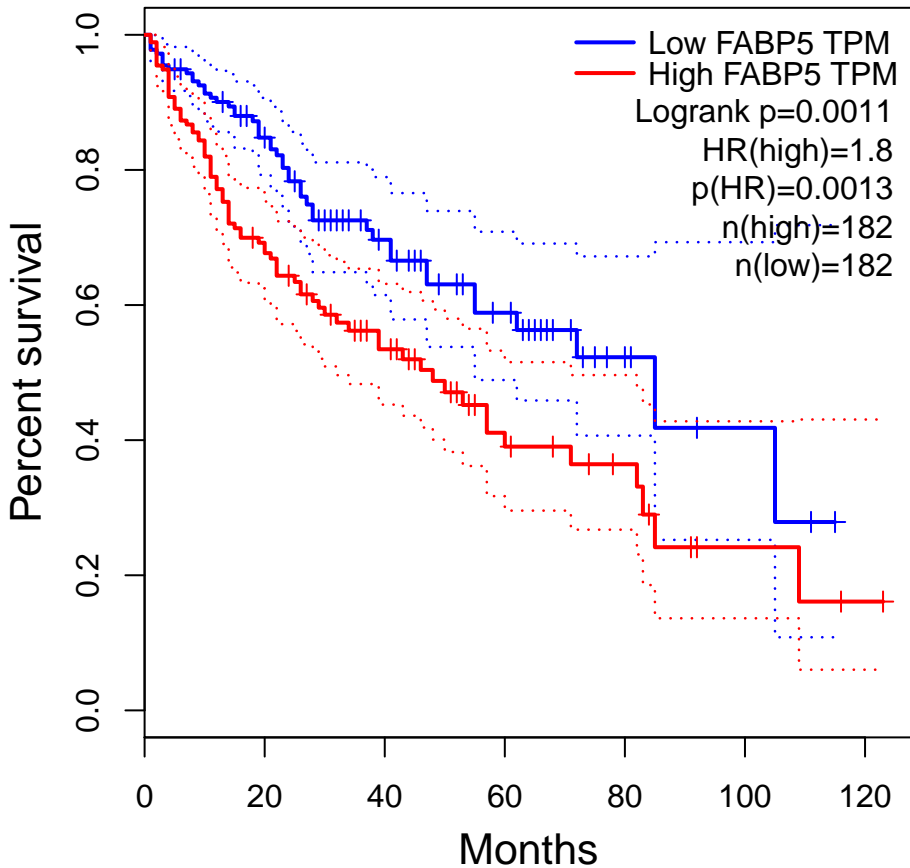

Supplement: Supplementary file 1 — Supplementary Information. [file 41598_2023_30695_MOESM1_ESM.zip › Supplementary Data/╩2╛▌/Survival analysis/OS/LIHC0.001.pdf]

# Overall Survival

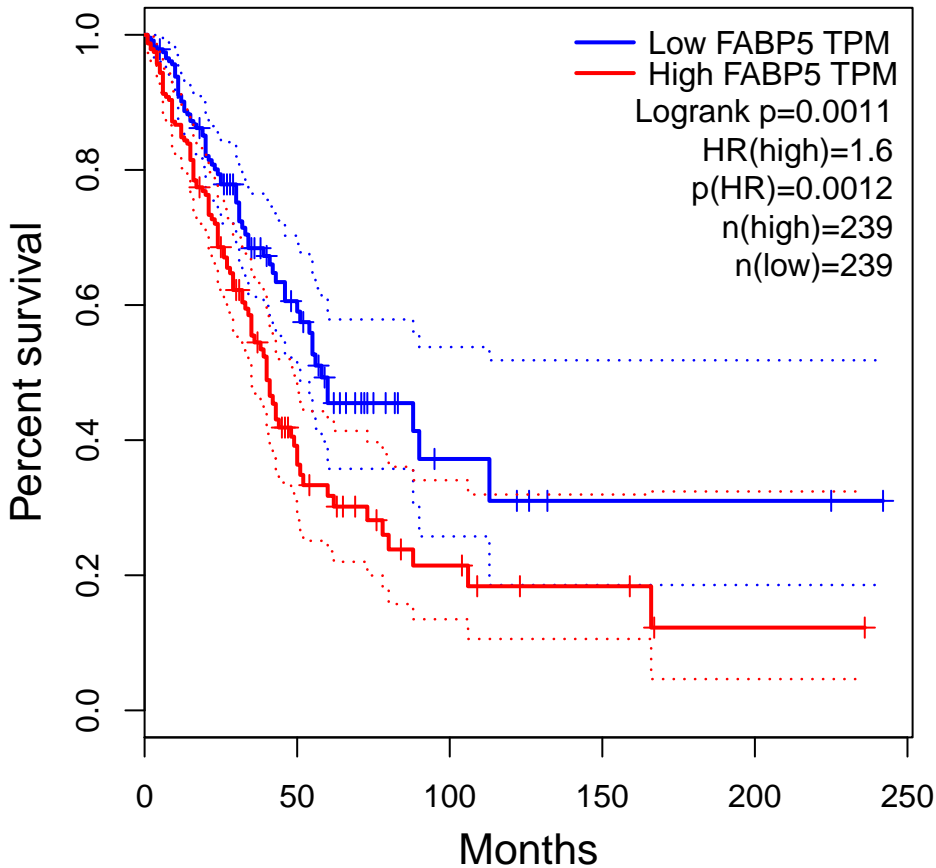

Supplement: Supplementary file 1 — Supplementary Information. [file 41598_2023_30695_MOESM1_ESM.zip › Supplementary Data/╩2╛▌/Survival analysis/OS/LUAD0.001.pdf]

# Overall Survival

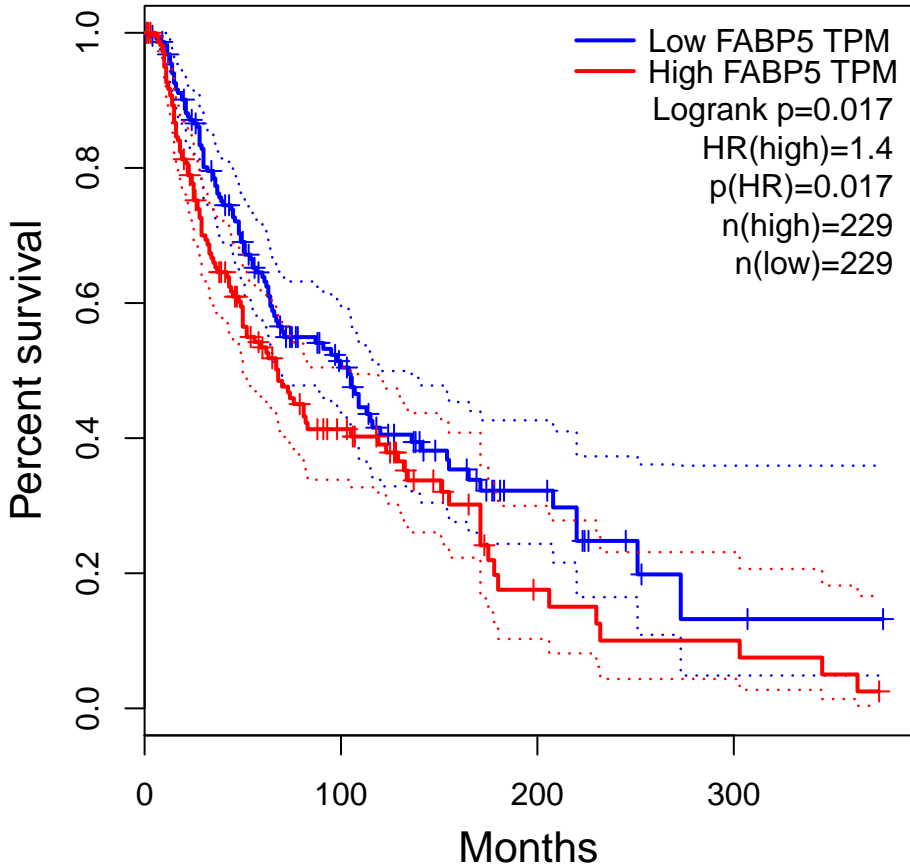

Supplement: Supplementary file 1 — Supplementary Information. [file 41598_2023_30695_MOESM1_ESM.zip › Supplementary Data/╩2╛▌/Survival analysis/OS/SKCM0.017.pdf]

# Overall Survival

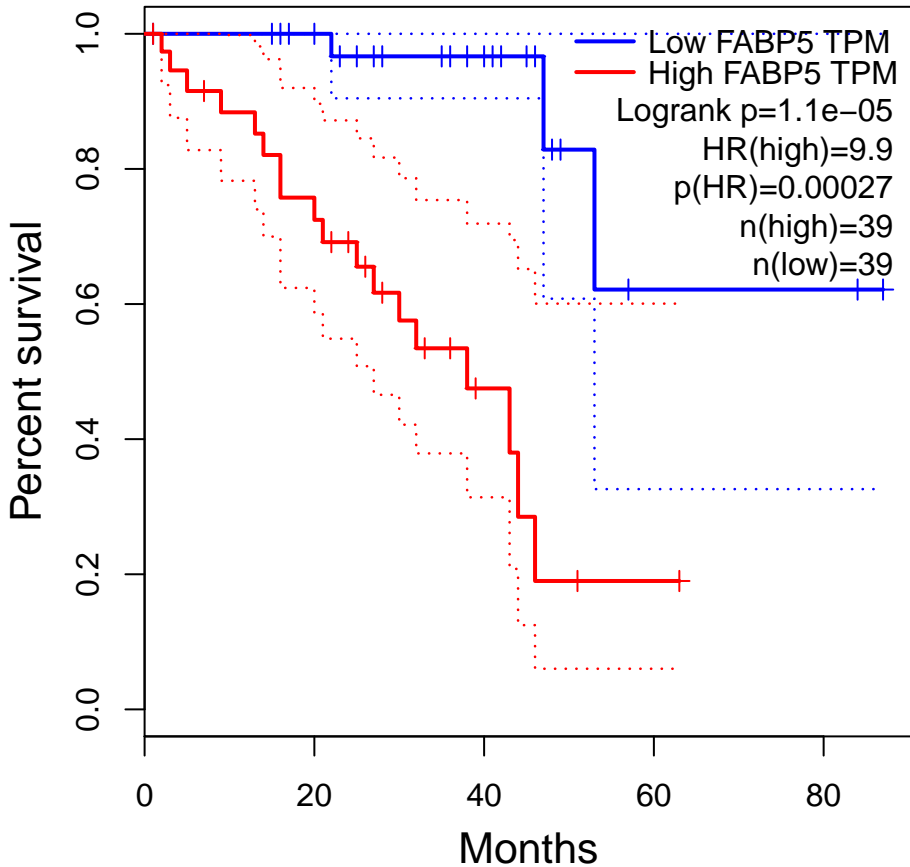

Supplement: Supplementary file 1 — Supplementary Information. [file 41598_2023_30695_MOESM1_ESM.zip › Supplementary Data/╩2╛▌/Survival analysis/OS/UVM╨í╙┌0.001.pdf]
